# Aprepitant and fosaprepitant as a prophylactic antiemetic for preventing postoperative nausea and vomiting after general anaesthesia: a systematic review and meta-analysis

**DOI:** 10.1016/j.clinsp.2025.100783

**Published:** 2025-10-15

**Authors:** Thiago Ramos Grigio, Hans Timmerman, Natanael Pietroski dos Santos, José Eduardo Guimarães Pereira, Angela Maria Sousa, André Paul Wolff

**Affiliations:** a Department of Anesthesiology, Pain Centre, University of Groningen, University Medical Centre Groningen, Groningen, the Netherlands; b Postgraduate Program of Anaesthesiology, Surgical Sciences and Perioperative Medicine, Faculdade de Medicina da Universidade de São Paulo (USP), São Paulo, SP, Brazil; c Centro Universitário Faculdade de Medicina do ABC, Santo André, SP, Brazil; d Department of Anesthesiology, Hospital Central do Exército, Rio de Janeiro, RJ, Brazil

**Keywords:** Aprepitant, Evidence-based medicine, Fosaprepitant, Perioperative care, Postoperative emesis, Postoperative vomiting, Postoperative nausea

## Abstract

**Background:**

Postoperative Nausea and Vomiting (PONV) is one of the patient-important outcomes, as its management reduces morbidity associated with surgery. This systematic review with meta-analysis aims to evaluate the efficacy and safety of prophylactic aprepitant and fosaprepitant within the first 24 hours after surgery in adult patients who underwent general anaesthesia. The authors The authors combined the outcomes only with identical evaluation times.

**Methods:**

The authors The authors searched six databases (MEDLINE, EMBASE, CENTRAL, WHO, ICTRP, SCIELO) for randomized controlled trials that compared aprepitant or fosaprepitant for PONV against control in different time assessments within the first 24 hours after surgery in adult patients who underwent general anaesthesia.

**Results:**

This systematic review includes 35 articles with 6241 participants in total. The authors The authors observed that aprepitant, from 0h to 24 h after surgery, significantly reduces the incidence of nausea (RR = 0.80, 95 % CI 0.67–0.97, I^2^ = 42 %, *p* = 0.07), vomiting (RR = 0.41, 95 % CI: 0.31–0.55, I^2^ = 51 %, *p* = 0.008), the use of rescue antiemetics (RR = 0.79, 95 % CI 0.66–0.95, I^2^ = 54 %, *p* = 0.009), and increases the complete response rate (RR = 1.19, 95 % CI 1.04–1.37, I^2^ = 51 %, *p* = 0.04). On the other hand, fosaprepitant, from 0 to 24 hours after surgery, showed a reduction only in the incidence of vomiting (RR = 0.35, 95 % CI 0.19–0.64, I^2^ = 33 %, *p* = 0.20).

**Conclusion:**

Aprepitant reduces the incidence of postoperative nausea, vomiting and the use of rescue antiemetics and increases the complete response rate among adult patients from 0 to 24 hours after surgery. Fosaprepitant reduces the incidence of vomiting from 0 to 24 hours after surgery. Findings primarily reflect female patients; male applicability requires further study.

**PROSPERO registry:**

CRD42023427076.

## Highlights

• Aprepitant reduces nausea, vomiting, and rescue antiemetics post-surgery.
• Aprepitant increases the complete response rate within 24 hours post-surgery.
• Fosaprepitant reduces vomiting but not nausea or rescue antiemetic use.
• The review included 35 studies with 6241 participants under general anesthesia.
• Identical time points were analyzed to assess the efficacy and safety of both drugs.

## Introduction

Postoperative Nausea and Vomiting (PONV) is one of the patient-important outcomes as its management reduces morbidity associated with surgery.^1^^,^^2^ Despite adequate prophylaxis, the incidence of current PONV can reach up to 63 %^3^ in high-risk patients, according to the Apfel score.^4^ Nausea and vomiting are the most feared symptoms after surgery,^5^ and they are related to poorer patient satisfaction, discomfort, and unexpected results like dehydration, altered electrolytes, and pulmonary aspiration of gastric contents.^2^ Studies about PONV gained increased attention in the 1950s and 1960s^6^ and, to the present day, contribute to improving health care by helping patients, physicians, and policymakers make cost-effective decisions.

Forty-four single drugs with antiemetic properties are currently available for perioperative use.^7^ These medications are divided into six subcategories: NK_1_ receptor antagonist, 5-HT_3_ receptor antagonist, D_2_ receptor antagonist, corticosteroid, antihistamine and anticholinergic.^7^ Each drug group acts on receptors responsible for the physiology of nausea and vomiting. Among the NK_1_ receptor antagonists, aprepitant and fosaprepitant play an essential role in multimodal prophylaxis involving the different types of receptors. Aprepitant is originally taken orally in capsule form (40 mg, 80 mg and 125 mg), and from 2022 also been released for intravenous use^8^ (32 mg). The prodrug fosaprepitant (115 mg), on the other hand, is infused intravenously and is rapidly converted to active aprepitant, which produces similar systemic exposure.^9^ Fosaprepitant 115 mg achieves a bioequivalent area under the curve to aprepitant at a dose of 125 mg.^10^

Combining antiemetics often results in better prevention of nausea and vomiting than using a single medication alone. Nonetheless, the use of individual NK_1_ receptor antagonists displayed efficacy comparable to that of many combined drug prophylaxis.^7^ Aprepitant, with a high level of evidence, is the most effective antiemetic for vomiting in the first 24 hours after surgery (Risk Ratio [RR] 0.26 (0.18‒0.38), and fosaprepitant, although potentially presenting excellent results (RR = 0.06 [0.02‒0.21]), has moderate evidence for its use as antiemetic.^7^ The published meta-analyses^7^^,^^11^^,^^12^ combined the outcomes of nausea and/ or vomiting with different evaluation times in just one period, 0 and 24 hours. No systematic review evaluates outcomes at various times within the first 24 hours after surgery. Moreover, despite the absence of serious adverse effects with the use of NK_1_ receptor antagonists, there is still little information on the side effects of aprepitant and especially fosaprepitant.^7^

The objective of this systematic review with meta-analysis of randomised controlled trials is to evaluate the efficacy and safety of prophylactic aprepitant and fosaprepitant according to different time assessments within the first 24 hours after surgery in adult patients who underwent general anaesthesia.

## Methods

This systematic review and meta-analysis of Randomised Controlled Trials (RCTs) was conducted following the guidelines of the Preferred Reporting Items for Systematic Reviews and Meta-analysis (PRISMA).^13^ The study protocol was registered on May 27, 2023 and updated on January 7, 2025 in the International Prospective Register of Systematic Reviews (PROSPERO 2023 CRD42023427076). No adjustments to the protocol were made during the execution of this review.

### Eligibility criteria

Only Randomised Controlled Trials (RCTs) that compared aprepitant or fosaprepitant alone, combined or not with other antiemetics for PONV against an inactive control, another antiemetic drug, or a combination of other antiemetic drugs were eligible. Studies with prophylactic aprepitant or fosaprepitant administered preoperatively or intraoperatively with all routes of administration and all doses were included. Adult participants (at least 18-years-old) who underwent any surgical procedure under general anaesthesia and participants who were evaluated at least any time assessment within 24 hours after surgery were included in this study. There were no language restrictions, and the date of publication was recorded. The authors The authors excluded: 1) non-RCTs; 2) quasi-RCTs; 3) Retracted studies; 4) Animal studies; 5) Not full-text journal publications; 6) Participants who stayed less than 24 hours in the hospital; 7) Surgical procedures limited to regional anaesthesia or sedation; 8) None of prespecified outcomes reported; and 9) Non-prophylactic use of aprepitant or fosaprepitant.

The endpoints of this study were to evaluate the efficacy and safety of aprepitant and fosaprepitant. Therefore, the primary outcomes included the number of participants with postoperative nausea and vomiting (or retching). The secondary outcomes were complete response, the requirement of additional antiemetics in the postoperative period, and any adverse events within 0 to 24 hours postoperative. The outcomes were assessed at any time within 24 hours after surgery, and similar time points were pooled together. In addition, the outcomes collected correspond to the RCTs’ primary and secondary outcomes.

### Information sources

A medical librarian and the first author (TRG) performed a systematic search in MEDLINE (via PubMed); EMBASE (via Elsevier); Web of Science; Cochrane Central Register of Controlled Trials (CENTRAL); World Health Organization (WHO) International Clinical Trials Registry Platform (ICTRP); Clinical Trials Results; SCIELO; and Grey Literature Report. Reference lists in identified studies were also reviewed for additional studies. The bibliographic records retrieved were imported and de-duplicated in EndNote. The initial search was performed in May 2023, and updated in December 2024. The headings used for the search strategy were aprepitant, fosaprepitant, postoperative nausea and vomiting, and all their synonyms. The complete search strategies for all databases are presented in Appendix 1.

### Selection process

**Fig. 1:**
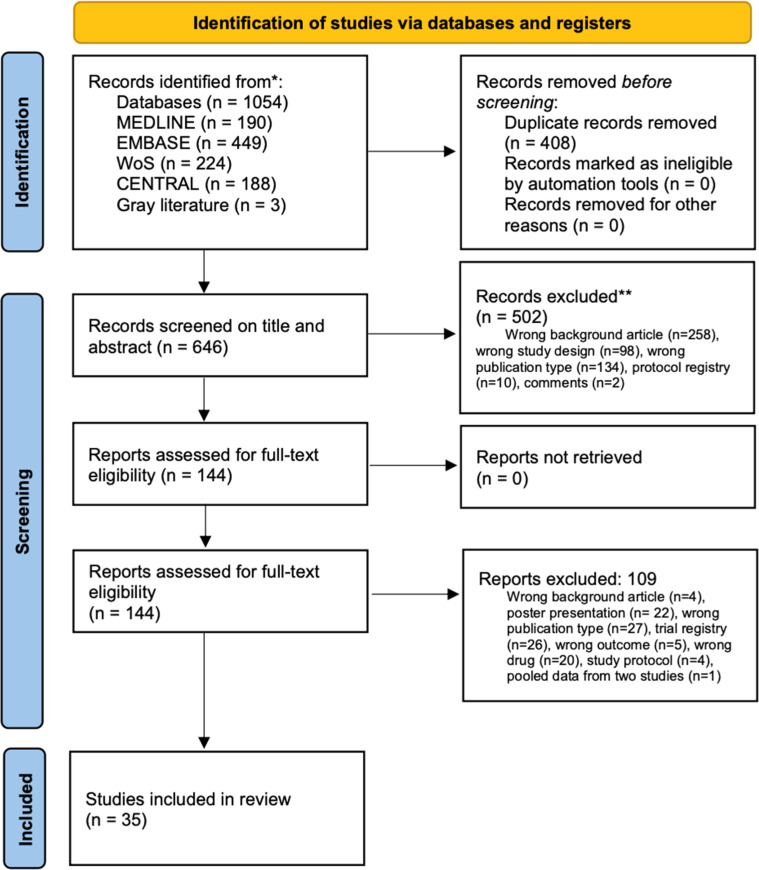
Search strategy according to PRISMA (Preferred Reporting Items for Systematic Review and Meta-Analysis).

Two reviewers (TRG and HT) independently assessed all identified studies for eligibility using the Rayyan platform (Rayyan, Doha, Qatar).^14^ The authors The authors screened all titles and abstracts retrieved, and articles that met the inclusion criteria were marked as ‘potentially eligible’; after that, the authors the authors performed eligibility screening of the full text of the ‘potentially eligible’ articles. Disagreements were resolved by discussing or consulting a third reviewer (APW). The review process results are documented in a PRISMA flow diagram (Fig. 1).

### Data extraction

One author (TRG) used a data-extraction form to extract data from eligible studies. The second reviewer (HT) checked for accuracy. The authors The authors extracted the number of events and participants for both experimental and control groups for all outcomes. The authors The authors contacted authors of studies without available data via email.

### Data items

The following data were collected: bibliographic information (name of the first author, year of publication, country), participants' characteristics (sex), surgical and anaesthesia procedure (type of surgery, anaesthesia maintenance), intervention and comparison characteristics (medicine, dose), outcome measures (nausea, vomiting, retching, complete response or treatment completion, rescue antiemetic use, adverse events, timing of outcome assessment). Table 1 shows the characteristics of included studies.

**Table 1:** Characteristics of included studies. TIVA, total intravenous anaesthesia.

											Time frame (hours)								
	First author, year	Country	N. of participants	Population ( % female)	Type of Surgery	Anesthesia maintenance	N. of groups	Intervention	Comparison	Measured outcomes	0–24	0–2	0–6	2–6	6–24	2–24	12–24	Other	Adverse events reported
Aprepitant	Alam, 2023[18]	Iran	80	Adults (50)	Orthognathic surgery (Lefort I maxillary advancement osteotomy and bilateral sagittal split osteotomy mandibular setback surgery with the Dalpont method)	Inhalational anesthesia (sevoflurane)	2	Aprepitant 80mg	Ondansetron 4mg	Nausea		★		★			★	6–12h	Vertigo, blurred vision, headache, drowsiness
										Vomiting	★								
										Rescue antiemetic	★								
										Complete response (no postoperative vomiting and no rescue antiemetic use)	★								
	Alonso-Damián, 2012[19]	Mexico	60	Adults (80)	Open colecystectomy	Inhalational anesthesia (sevoflurane)	2	Aprepitant 80mg	Ondansetron 4mg	Nausea								At 6 and 24	Constipation
										Vomiting								At 6 and 24	
	Ashoor, 2022[20]	Egypt	86	Adults (40)	Laparoscopic sleeg gastrectomy	Inhalational anesthesia (sevoflurane)	3	Aprepitant 80 mg + dexamethasone 8mg	Mirtazapine 30 mg + dexamethasone or dexamethasone 8gm only	Nausea	★	★				★			Headache, dizziness, dry mouth, diarrhea
										Vomiting	★	★				★			
										Nausea and vomiting	★	★				★			
										Complete response (defined as patients experiencing VAS nausea score ≤4 and no use of rescue therapy 0–24 h after surgery)	★	★				★			
	Bergese, 2016[22]	USA	95	Adults (54)	Craniotomy	Inhalational anesthesia (desflurane, sevoflurane or isoflurane)	2	Apreprepitant 40 mg + dexamethasone 10 mg + prometazine 25mg	Ondansetron 4 mg + dexamethasone 10 mg + prometazine 25mg	Nausea	★	★							No adverse events
										Vomiting	★	★							
										Nausea and/or vomiting	★	★							
										Rescue antiemtic	★	★							
	Bilgen, 2018[23]	Turkey	67	Adults (81)	Laparoscopic gynecological surgery or laparoscopic cholecystectomy	Inhalational anesthesia (sevoflurane)	2	Apreprepitant 40 mg + dexamethasone 8mg	Dexamethasone 8mg	Vomiting or Retching	★	★				★			Not reported
										Rescue antiemtic	★								
										Complete response (no nausea (VRS<4), no retching, no vomiting and no rescue therapy)	★								
	de Morais, 2018[25]	Brazil	66	Adults (100)	Laparoscopic intermediate procedures to abdominal or pelvic cancer	TIVA	2	Aprepitant 80 mg + dexamethasone 4–8 mg + ondansetron 4–8 mg	Dexamethasone 4–8 mg + ondansetron 4–8 mg	Nausea and/or vomiting	★	★				★			Hypotension, pruritus
										Nausea	★	★							
										Vomiting	★	★							
										Rescue antiemtic	★								
	Diemunsch, 2007[26]	USA, Canada, South America, Europa, Australia, Asia	892	Adults (91)	Open abdominal surgery gynecologic, prostatectomy, intestinal resection, hernia repair, bladder surgery, cholecystectomy or nephrectomy.	Inhalational anesthesia + nitrous oxide	3	Aprepitant 40 mg or apreprepitant 125mg	Ondansetron 4mg	Vomiting	★								Constipation, headache, pyrexia, bradycardia
										Rescue antiemtic	★								
										Complete response (no vomiting and no use of rescue therapy)	★								
	Gan, 2007[27]	USA	766	Adults (94)	Open abdominal surgery gynecologic, prostatectomy, intestinal resection, hernia repair, bladder surgery, cholecystectomy or nephrectomy.	Inhalational anesthesia + nitrous oxide	3	Aprepitant 40 mg or aprepitant 125mg	Ondansetron 4mg	Vomiting	★								Constipation, headache, pyrexia, bradycardia
										Rescue antiemtic	★								
										Complete response (no vomiting and no use of rescue therapy)	★								
	Gokdemir 2024[52]	Turkey	61	Adults (64)	Laparoscopic cholecystectomy	Inhalational anesthesia (sevoflurane)	2	Aprepitant 40mg	Granisetron 3mg	Nausea and vomiting			★				★	6–12h	Arrhythmia, hypotension, hypertension,
										Rescue antiemtic			★				★	6–12h	
	Grigio, 2020[28]	Brazil	91	Adults (100)	Mastectomy	TIVA	2	Aprepitant 80 mg + palonosetron 0.075 mg + dexamethasone 4mg	Palonosetron 0.075 mg + dexamethasone 4mg	Nausea	★	★		★					Not reported
										Vomiting	★	★		★					
										Nausea and vomiting	★	★		★					
										Rescue antiemtic	★								
	Habib, 2011[29]	USA	104	Adults (56)	Craniotomy	Inhalational anesthesia (isoflurane)	2	Aprepitant 40 mg + dexamethasone 10mg	Ondansetron 4 mg + dexamethasone 10 mg	Nausea	★	★							Headache, sedation
										Vomiting	★	★							
										Rescue antiemtic	★	★							
										Complete response (no PONV and no need for rescue antiemetics)	★	★							
	Ham, 2016[30]	South Korea	110	Adults (100)	Laparoscopic gynecologic surgery (total hysterectomy, ovarian cystectomy, ovarian cyst enucleation, myomectomy, salpingo-oophorectomy)	Inhalational anesthesia (sevoflurane)	2	Aprepitant 80 mg + ondansetron 4 mg	Ondansetron 4mg	Nausea	★		★						Headache, dizziness, sedation, delayed passage of flatus, pruritus
										Vomiting	★		★						
										Rescue antiemtic								0–48h	
										Complete response (no PONV and no rescue antiemetics)	★		★						
	Jayabalan, 2019[32]	India	120	Adults (100)	Simple mastectomy, modified radical mastectomy, total thyroidectomy, hemithyroidectomy	Inhalational anesthesia (isoflurane) (answer from author via email)	2	Aprepitant 40mg	Ondansetron 8mg	Nausea		★					★	2–12 h	Not reported
										Vomiting	★	★					★	2–12 h	
	Jung, 2013[33]	Republic of Korea	120	Adults (100)	Laparoscopic total hysterectomy	Inhalational anesthesia (isoflurane)	3	Aprepitant 80 mg or aprep 125mg	No antiemetic	Nausea		★				★			Dizziness, headache, dyspepsia, abdominal distension
										Vomiting or Retching		★				★			
										Rescue antiemtic								0–48h	
										Complete response (defined as no nausea, retching, or vomiting and no need for rescue therapy)		★				★			
	Kakuta, 2011[34]	Japan	60	Adults (100)	Laparoscopic gynecological surgery (ovarian systectomy/ tumorectomy, adhesioloysis, myomectomy, vaginal hysterectomy, salpingostomy)	Inhalational anesthesia (sevoflurane)	2	Aprepitant 80mg	No antiemetic	Nausea		★				★			Not reported
										Vomiting		★				★			
										Nausea and/or vomiting		★				★			
										Rescue antiemtic		★				★			
	Kawano, 2015[36]	Japan	60	Adults (100)	High tibial osteotomy or total knee arthroplasty	Inhalational anesthesia (sevoflurane)	2	Aprepitant 80 mg + dexamethasone 8mg	Dexamethasone 8mg	Nausea	★	★				★			Headache, dizziness
										Vomiting	★	★				★			
										Nausea and/or vomiting	★	★				★			
										Rescue antiemtic	★	★				★			
										Complete response (defined as no PONV and no rescue antiemetic use)	★	★				★			
	Lee, 2012[37]	Republic of Korea	84	Adults (100)	Gynecological surgery	Inhalational anesthesia (desflurane)	2	Aprepitant 80 mg + ramosetron 0.3mg	Ramosetron 0.3mg	Nausea	★		★		★				Dizziness, headache, sedation
										Vomiting	★		★		★				
										Nausea and vomiting	★								
										Rescue antiemetic	★		★		★				
	Lim, 2013[38]	Republic of Korea	52	Adults (42)	Rhinolaryngological surgery (tonsillectomy, throidectomy, endoscopi sinus surgery or laryngomicrosurgery)	Inhalational anesthesia (desflurane)	3	Aprepitant 80 mg + ondansetron 4 mg or aprepitant 125 mg + ondansetron 4mg	Ondansetron 4mg	Nausea			★						No adverse events
										Rescue antiemetic			★						
										Nausea and vomiting			★		★				
	Long, 2014[39]	USA	94	Adults (100)	Hysterectomy (open, robotic, vaginal)	Inhalational anesthesia (sevoflurane)	2	Aprepitant 40 mg + dexamethasone 8 mg + ondansetron 4mg	Dexamethasone 8 mg + ondansetron 4mg	Nausea	★								Wound dehiscence, paresthesia, wound infection, blood clot in leg, bradycardia, chylous ascites, hospital-aquired pneumonia, hot flashes, stomach pain, valley fever, anemia, back pain, clostridium difficile, constipation, gas pain, urinary retention, headache
										Vomiting	★								
										Rescue antiemetic	★								
	Moon, 2014[49]	Republic of Korea	93	Adults (100)	Laparoscopic gynecological surgery	Inhalational anesthesia (desflurane) + nitrous oxide	2	Aprepitant 40mg	Palonosetron 0.075mg	Rescue antiemetic		★		★	★				Not reported
										Complete response (no nausea (VRS<4), and no rescue therapy)								0–48h	
	Ortiz, 2024[50]	Mexico	400	Adults (94)	Laparoscopic sleeve gastrectomy	Inhalational anesthesia (sevoflurane)	2	Aprepitant 80 mg + dexamethasone 10 mg + ondansetron 4 mg + metoclopramide 10mg	Dexamethasone 10 mg + ondansetron 4 mg + metoclopramide 10mg	Nausea								At 6, 12, and 24h	
										Vomiting									
										Retching									
										Rescue antiemetic									
	Patro, 2022[40]	India	70	Adults (100)	Laparoscopic surgery	Inhalational anesthesia (sevoflurane) + nitrous oxide	2	Aprepitant 40mg	Palonosetron 0.075mg	Nausea and vomiting		★		★			★	6–12h	Not reported
										Rescue antiemtic								0–48h	
										Treatment completion (no postoperative nausea and vomiting)								0–48h	
	Sinha, 2014[41]	USA	124	Adults (65)	Laparoscopic bariatric surgery	Inhalational anesthesia (desflurane or sevoflurane)	2	Aprepitant 80 mg + ondansetron 4 mg	Ondansetron 4mg	Complete response (no nausea or vomiting without rescue antiemetics)								0–72h	Not reported
	Shivakarmar 2024[51]	India	80	Adults (55)	Elective surgeries	Inhalational anesthesia (isoflurane)	2	Aprepitant 80mg	Ondansetron 4mg	Nausea	★								
										Vomiting	★								
										Rescue antiemetic	★								
	Thanuja, 2021[43]	India	96	Adults (100)	Laparoscopic cystectomy, diagnostic hysterolaparoscopy, or laparoscopic sterilisation	Inhalational anesthesia (sevoflurane)	3	Aprepitant 80 mg alone or aprepitant 80 mg + ondansetron 4mg	Dexamethasone 8 mg + ondansetron 4 mg	Nausea								0–4h	
										Vomiting								0–4h	
										Nausea and vomiting								0–4h	
										Rescue antiemtic								0–4h	
	Vallejo, 2012[45]	USA	150	Adults (93)	Ambulatory plastic surgery (Breast, abdominal, eye, melanoma, nose, other)	Inhalational anesthesia (sevoflurane)	2	Aprepitant 40 mg + ondansetron 4mg	Ondansetron 4mg	Vomiting	★						★	0–12h	Not reported
										Rescue antiemtic								0–48h	
										Complete response (absence of vomiting and no need of any rescue antiemetics)								0–48h	
	Wajid, 2022[46]	Pakistan	314	Adults (50)	Laparoscopic cholecystectomy	Inhalational anesthesia (isoflurane)	2	Aprepitant 80mg	Ondansetron 8mg	Nausea and vomiting	Not clear								NA
	Yeo, 2018[47]	Republic of Korea	187	Adults (65)	Elective surgeries	Inhalational anesthesia + nitrous oxide	2	Aprepitant 80mg	No antiemetic	Nausea and vomiting								0–48h	No serious complications, including asthenia, fatigue, hiccups, constipation, diarrhea and anorexia
										Vomiting or Retching								0–48h	
	Yoo, 2018[48]	Republic of Korea	85	Adults (100)	Major orthopedic operation, thyroidectomy, laparoscopic hysterectomy, laparoscopic cholecystectomy	Inhalational anesthesia (desflurane or sevoflurane)	2	Aprepitant 80 mg + palonosetron 0.075mg	Palonosetron 0.075mg	Nausea	★		★		★				Dizziness, headache
										Vomiting	★		★		★				
										Nausea and vomiting	★		★		★				
										Rescue antiemtic	★		★		★				
Fosaprepitant	Atsuta, 2017[21]	Japan	186	Adults (58)	Craniotomy	TIVA	2	Fosaprepitant 150 mg + dexamethasone 9.9 mg (except patients with DM)	Droperidol 1.25 mg + dexamethasone 9.9 mg (except patients with DM)	Vomiting		★		★	★				Sedation
										Rescue antiemtic		★		★	★				
										Complete response (no postoperative nausea and vomiting and no rescue antiemetic)		★		★	★				
	Braga, 2022[24]	Brazil	88	Adults (100)	Laparoscopic cholecystectomy	Inhalational anesthesia (sevoflurane)	2	Fosaprepitant 150mg	Palonosetron 0.075mg	Nausea	★	★		★	★				Headache, dizziness, sleepness, weakness
										Vomiting	★	★		★	★				
										Rescue antiemtic								0–48h	
										Complete response (no postoperative nausea, vomiting and no rescue antiemetic) (author replied via email)								0–48h	
	Huang, 2023[31]	China	1154	Adults (97)	Laparoscopic gastrointestinal surgery (gastrectomy or small intestinal resection, colon resection, rectum resection or surgery on another site)	TIVA	2	Fosaprepitant 150 mg + dexamethasone 5 mg + palonosetron 0.075mg	Dexamethasone 5 mg + palonosetron 0.075mg	Nausea and vomiting	★								Intraoperative hypotension, hypertension
										Vomiting	★								
										Nausea	★								
										Rescue antiemtic	★								
	Kakuta, 2015[35]	Japan	38	Adults (63)	Lower limb surgery (total hip arthroplasty, total knee arthroplasty, and rorational acetabular osteotomy)	Inhalational anesthesia (desflurane or sevoflurane)	2	Fosaprepitant 150mg	Ondansetron 4mg	Nausea		★				★			Not reported
										Vomiting	★	★							
										Nausea and vomiting	★	★							
										Rescue antiemtic	★	★							
										Complete response (no vomiting and no rescue antiemetic use)	★	★							
	Soga, 2015[42]	Japan	44	Adults (100)	Abdominal total hysterectomy, or bilateral salpingo-oophorectomy	Inhalational anesthesia (sevoflurane)	2	Fosaprepitant 150mg	Ondansetron 4mg	Nausea	★	★							Not reported
										Vomiting	★	★							
										Nausea and vomiting	★	★							
										Complete response (no vomiting and no rescue antiemetic use)	★	★							
	Tsutsumi, 2014[44]	Japan	64	Adults (59)	Craniotomy	TIVA	2	Fosaprepitant 150mg	Ondansetron 4mg	Vomiting	★	★							Not reported
										Nausea and vomiting	★	★							
										Rescue antiemtic	★	★							
										Complete response (no postoperative vomiting and no rescue antiemetic use)	★	★							
											**Time frame (hours)**								

Nausea was defined as an unpleasant sensation of having the urge to vomit. Vomit was described as a physical event as a forceful expulsion of gastric contents through the mouth. Retching was considered when the content of the gastrointestinal tract was forced into the oesophagus without expulsion of the vomitus.^15^ Complete response or treatment completion were considered synonyms and evaluated regardless of the specific definition, since there was variability among the included studies. Rescue antiemetic use was a postoperative rescue antiemetic at any time on the patient request. All these variables were dichotomous (yes/no).

### Study risk of bias assessment

The methodological quality of selected studies was evaluated using the tool suggested by the National Heart, Lung, and Blood Institute (NHLBI).^16^ The tool (Appendix) is composed of 14 questions of quality assessment. It includes questions about description as randomized, allocation concealment (two items), blinding, the similarity of groups at baseline, dropout (two items), adherence, avoidance of other interventions outcome measures assessment, power calculation, prespecified outcomes, and Intention-To-Treat analysis (ITT).

Before using this tool, two articles on a different topic were randomly selected to assess the level of understanding of the questions. Each question was discussed, and it was verified that both authors (TRG and HT) had the same understanding of the meaning of the questions as stated in this scale.

Two review authors (TRG and HT) independently evaluated the risk of bias, and disagreements were solved by a third reviewer (APW). Each question was graded as ‘yes’, ‘no,’ or ‘unclear’ / ‘not reported’ / ‘not applicable’. These answers reflect a low, high, and uncertain risk of bias, respectively. A low risk of bias translates to a rating of good quality, and a high risk of bias translates to a rating of poor quality. The authors The authors considered a ‘fatal flaw’ as any of the following: 1) Dropout rates > 20 %, 2) Differential dropout > 15 %, 3) Absence of intention-to-treat analysis, or 4) Use com completers-only analysis without justification. This definition follows guidance from the NHLBI quality assessment tool.^16^ The authors The authors created a table with the reviewers’ answers for each of the 14 questions (Table S1 ‒ Quality assessment of controlled intervention studies). For each risk of bias item, the authors the authors reported in detail the results of bias in each study. The publication bias was visually evaluated by funnel plots, contour enhanced, and statistically by Egger’s test. Meta-analyses with fewer than ten studies only underwent a visual analysis of the risk of publication bias.

### Data analysis

The statistical analyses were conducted using the Review Manager 5.4.1 software (The Cochrane Collaboration, London, United Kingdom).^17^ Meta-analyses were performed for each outcome with at least five studies available per intervention drug. The authors The authors divided the results of the meta-analyses into aprepitant and fosaprepitant. Risk ratios were used for dichotomous variables (nausea, vomiting, complete response or treatment completion, rescue antiemetic use) to compare the likelihood of an event occurring in the intervention group versus the control group. A 95 % Confidence Interval was used to indicate the range of values that the authors the authors can be 95 % confident that the true effect would lie within the lower and upper limits of the confidence interval.

The authors used a random-effects model to combine results from studies, accounting for both within-study and between-study variability. The level of heterogeneity between studies was assessed by I^2^. I^2^ test > 50 % represents substantial inconsistency among RCTs. Statistical heterogeneity was considered using the Chi-Square test (*p* < 0.05 as a statistically significant cut-off value).

Studies with two or more intervention or control arms were added and placed in a single intervention or control group, except in the subgroup analysis.

### Subgroup analysis and meta-regression

The authors performed separate subgroup analyses because clinical heterogeneity across studies was anticipated. Predefined subgroups analyses were: 1) Doses of aprepitant (40 mg, 80 mg and 125 mg), 2) Type of anaesthesia (Total Intravenous Anaesthesia [TIVA] and inhalational anaesthesia), 3) Type of surgery (high-risk surgeries (laparoscopic, bariatric, gynecological surgery, and cholecystectomy) versus low-risk surgeries for PONV (surgeries other than laparoscopic, bariatric, gynecological surgery, and cholecystectomy), 4) Mono or combination prophylaxis (aprepitant/fosaprepitant as single antiemetic or aprepitant/fosaprepitant plus other antiemetics), 5) “Pure effect of aprepitant” or aprepitant versus other antiemetics, and 6) Sex.

When at least ten studies were available for each outcome, the authors conducted a mixed-effects meta-regression analysis using the metafor package (version 4.6–0) in *R* (version 2024.12.1 + 563). This analysis aimed to investigate potential sources of heterogeneity by including the following moderators: 1) Type of surgery, categorized as high-risk, low-risk, or mixed/unknown when both or neither were specified, 2) Type of anesthesia, classified as Total Intravenous Anesthesia (TIVA) or inhalational anesthesia, coded as 0 and 1, respectively, 3) Dose of aprepitant or fosaprepitant in milligrams, and 4) Prophylactic strategy, defined as monotherapy (coded as 1) or combination therapy (coded as 0). In cases where multiple doses were reported for the same outcome, the arithmetic mean of the doses was used in the analysis.

### Sensitivity analysis

The authors performed sensitivity analysis to assess the present findings and explain study heterogeneity. The authors excluded RCTs with a high risk of bias and included only RCTs with low risk of bias (studies without fatal flaws according to the tool suggested by the National Heart, Lung, and Blood Institute (NHLBI). The authors also performed trim-and-fill analysis for the funnel plots with more than ten studies.

## Results

### Study results

The search strategy (Appendix A) identified 1054 manuscripts, and 408 duplicates were removed, leaving 646 studies to be screened on title and abstract (search date on May 27, 2023; updated on December 19, 2024). Agreement on screening abstract was 95 % between authors (TRG and HT). Of these, 502 studies were excluded. One hundred forty-four studies were assessed for full-text eligibility, and 109 studies were excluded. There was no disagreement among the authors regarding the included studies. The authors included 35 peer-reviewed RCTs^18^, ^19^, ^20^, ^21^, ^22^, ^23^, ^24^, ^25^, ^26^, ^27^, ^28^, ^29^, ^30^, ^31^, ^32^, ^33^, ^34^, ^35^, ^36^, ^37^, ^38^, ^39^, ^40^, ^41^, ^42^, ^43^, ^44^, ^45^, ^46^, ^47^, ^48^, ^49^, ^50^, ^51^, ^52^ in this systematic review and meta-analysis. The study selection procedure flow diagram is presented in Fig. 1. The authors were contacted via e-mail when necessary to clarify any questions regarding the published results.

### Study characteristics

Table 1 lists the characteristics of the 35 included studies. All the studies were published in English, except one^19^ in Spanish. The included trials were published between April 2007 and December 2024. In total, 6241 participants were included. Eighty-six percent of the participants were female, all were 18 years old, and had ASA I to III.

All the studies evaluated the participants within a period (in different time intervals) in the first 24 hours after surgery, except for two studies^19^^,^^50^ which evaluated the outcomes only at the exact moment of the evaluation (at 6 hours, 12 hours, and 24 hours after surgery). The time intervals taken to assess the patient outcomes of each study varied and are stated in Table 1.

Twenty-nine studies^18^, ^19^, ^20^^,^^22^^,^^23^^,^^25^, ^26^, ^27^, ^28^, ^29^, ^30^^,^^32^, ^33^, ^34^^,^^36^, ^37^, ^38^, ^39^, ^40^, ^41^^,^^43^^,^^45^, ^46^, ^47^, ^48^, ^49^, ^50^, ^51^, ^52^ investigated oral aprepitant and consisted of 4667 participants (aprepitant group: 2647 participants; control group: 2018 participants), and six studies^21^^,^^24^^,^^31^^,^^35^^,^^42^^,^^44^ investigated intravenous fosaprepitant and consisted of 1574 participants (fosaprepitant group: 790 participants; control group: 784 participants). Thirty studies^18^, ^19^, ^20^, ^21^, ^22^, ^23^, ^24^, ^25^^,^^28^, ^29^, ^30^^,^^32^, ^33^, ^34^, ^35^, ^36^, ^37^, ^38^, ^39^, ^40^, ^41^, ^42^, ^43^, ^44^, ^45^^,^^47^, ^48^, ^49^^,^^51^^,^^52^ included fewer than 200 participants, and four aprepitant studies evaluated a total of respectively 314,^46^ 766,^27^ 892,^26^ and 40,0^50^ participants, and one fosaprepitant study included 115,4^31^ participants.

Three different oral doses of aprepitant were used: 40 mg, 80 mg and 125 mg, and only one dose of intravenous fosaprepitant (150 mg). No studies evaluated intravenous aprepitant.

The studies evaluated different types of surgery, with the majority (13 aprepitant studies,^20^^,^^23^^,^^25^^,^^30^^,^^33^^,^^34^^,^^40^^,^^41^^,^^43^^,^^46^^,^^49^^,^^50^^,^^52^ and two fosaprepitant studies)^24^^,^^31^ using laparoscopic surgery as the technique of choice. In 13 aprepitant studies,^18^^,^^19^^,^^26^^,^^27^^,^^32^, ^33^, ^34^^,^^40^^,^^46^^,^^47^^,^^49^^,^^51^^,^^52^ aprepitant was the only antiemetic used for PONV prevention, while in 15 aprepitant studies,^20^^,^^22^^,^^23^^,^^25^^,^^28^, ^29^, ^30^^,^^36^, ^37^, ^38^, ^39^^,^^41^^,^^45^^,^^48^^,^^50^ a combination of aprepitant and other antiemetics was used. One study^43^ evaluated aprepitant alone and in combination with other antiemetics. On the other hand, four fosaprepitant studies^24^^,^^35^^,^^42^^,^^44^ evaluated fosaprepitant as a single prophylactic antiemetic, and two^21^^,^^31^ studies combined fosaprepitant with other antiemetics for PONV prophylaxis.

Inhalation anaesthesia was the only maintenance anaesthetic technique in 27 aprepitant studies^18^, ^19^, ^20^^,^^22^^,^^23^^,^^26^^,^^27^^,^^29^^,^^30^^,^^32^, ^33^, ^34^^,^^36^, ^37^, ^38^, ^39^, ^40^, ^41^^,^^43^^,^^45^, ^46^, ^47^, ^48^, ^49^, ^50^, ^51^, ^52^ and three fosaprepitant studies.^24^^,^^35^^,^^42^ The remaining two aprepitant^25^^,^^28^ and three fosaprepitant^21^^,^^31^^,^^44^ studies used total intravenous anesthesia.

Across the trials, “complete response” was variously defined as 1) No postoperative vomiting and no rescue antiemetic use, 2) No postoperative nausea and vomiting and no rescue antiemetic use, 3) No postoperative nausea or vomiting and no rescue antiemetic use 4) No postoperative nausea, vomiting or retching and no rescue antiemetic use, 5) Visual Analogue Scale (VAS) nausea score < 4 and no use of rescue antiemetic therapy, 6) No nausea (Verbal Rating Scale [VRS < 4]) no retching, no vomiting and no rescue therapy, 7) No nausea (Verbal Rating Scale [VRS < 4]) and no rescue therapy, 8) No retching, no vomiting and no rescue therapy, no vomiting and no use of rescue therapy, no PONV and no need for rescue antiemetics, 9) No postoperative nausea and vomiting.

No standardization of the adverse effects was reported. The studies reported a wide variety of adverse effects. The most common side effects reported were headache, dizziness, sedation, and constipation.

### Risk of bias in studies

The raters agreed on 90 % of the items scored, and disagreements between assessors were resolved by discussion. The risk of bias is summarised in the supplementary online material (Table S1 ‒ Quality assessment of controlled intervention studies).

All studies were described as randomized clinical trials. Regarding allocation concealment, the method of randomization was adequate in most of the articles, except two,^19^^,^^34^ which did not report how the randomization was done. In addition, the treatment allocation concealment was not reported in seven studies^19^^,^^34^^,^^39^^,^^41^^,^^46^^,^^48^^,^^51^ and could not be determined in six studies.^23^^,^^35^^,^^37^^,^^38^^,^^50^^,^^52^ Participants and providers were blinded in 23 studies,^18^^,^^20^, ^21^, ^22^, ^23^, ^24^, ^25^, ^26^, ^27^, ^28^, ^29^, ^30^, ^31^, ^32^, ^33^^,^^35^^,^^40^, ^41^, ^42^, ^43^, ^44^, ^45^^,^^47^ and outcome assessors were blinded in 24.^18^^,^^20^, ^21^, ^22^, ^23^, ^24^, ^25^, ^26^^,^^28^, ^29^, ^30^, ^31^, ^32^, ^33^^,^^35^^,^^36^^,^^40^, ^41^, ^42^, ^43^, ^44^, ^45^^,^^47^^,^^49^ In two studies,^37^^,^^51^ the participants and providers were not blinded, and in one,^37^ the outcome assessors were not blinded. In addition, in 12 studies,^19^^,^^27^^,^^34^^,^^36^^,^^38^^,^^39^^,^^46^^,^^48^, ^49^, ^50^, ^51^, ^52^ it was not possible to determine, or the authors did not report whether the participants, providers, or the outcome assessors were blinded. In four studies,^25^^,^^39^^,^^43^^,^^46^ baseline characteristics were not reported, or it was not possible to determine if the groups were similar, and in one study,^19^ the groups were not similar at baseline on important characteristics. In one study,^48^ participants did not adhere to the protocols for assigned interventions, and in four studies^19^^,^^33^^,^^39^^,^^46^ it was not possible to determine, or it was not reported whether the participants in each treatment group adhered to the protocols. In all other 30 studies, there was high adherence to the protocol design. In only one study,^39^ whether the researchers avoided other interventions that were not part of the study protocol and could affect the outcomes being assessed was not reported. In one study,^34^ the outcomes assessed did not use valid and reliable measures, and two studies^19^^,^^46^ did not report the methods used to measure the outcomes. In three studies,^28^^,^^34^^,^^39^ it was not reported whether the sample size was sufficiently large to detect a difference in the primary outcome between groups with at least 80 % power using a two-sided alpha of 0.05. In one study,^19^ the power was not reported. All studies prespecified the outcomes, except one.^19^ Fourteen studies^18^^,^^20^^,^^21^^,^^23^^,^^28^^,^^29^^,^^32^^,^^34^^,^^35^^,^^38^^,^^43^^,^^47^, ^48^, ^49^ did not use an intention-to-treat analysis. In two studies,^39^^,^^46^ it was not possible to determine if an ITT analysis had been executed. In two studies,^25^^,^^39^ the overall drop-out rate from the study at endpoint was higher than 20 % and/or the differential drop-out rate between treatment groups at endpoint was 15 % higher. Four studies^22^^,^^33^^,^^36^^,^^46^ did not report the drop-out rate. Studies with high dropout rates, high differential dropout rates, or no ITT analysis are considered to have a significant risk of bias. Consequently, 16^18^^,^^20^^,^^21^^,^^23^^,^^25^^,^^28^^,^^29^^,^^32^^,^^34^^,^^35^^,^^38^^,^^39^^,^^43^^,^^47^, ^48^, ^49^ of the total 32 included studies were of poor quality.

### Outcomes

The meta-analyses were divided into the aprepitant/fosaprepitant and the control group. The authors only carried out meta-analyses of the separate incidence of symptoms because some authors reported the combined incidence of nausea and vomiting, and there was no uniformity in how it was reported (nausea and vomiting; nausea and/or vomiting; nausea or vomiting). The authors therefore analyzed the outcomes as a single outcome. Thirty studies reported at least the incidence of nausea or vomiting (or retching) separately in the first 24 hours after surgery. Authors from one study^40^ made the data available.

The outcomes were all reported when assessed in the first 24 hours after surgery. However, the time assessment varied between the studies. Twenty-three studies^18^^,^^20^^,^^22^, ^23^, ^24^, ^25^, ^26^, ^27^, ^28^, ^29^, ^30^, ^31^, ^32^^,^^35^, ^36^, ^37^^,^^39^^,^^42^^,^^44^^,^^45^^,^^48^^,^^51^^,^^52^ evaluated the incidence of at least one outcome between 0 and 24 hours, and 17 studies^18^^,^^20^, ^21^, ^22^, ^23^, ^24^, ^25^^,^^28^^,^^29^^,^^32^, ^33^, ^34^, ^35^, ^36^^,^^42^^,^^44^^,^^49^ evaluated between 0 and 2 hours after surgery. The other evaluation times had fewer than 5 studies, so no meta-analysis was carried out.

The main manuscript includes only forest plots of the results from 0 to 24 hours after surgery. The supplementary material contains meta-analyses with at least 5 included studies that evaluated other times between 0 and 24 hours after surgery.

Nausea

**Fig. 2:**
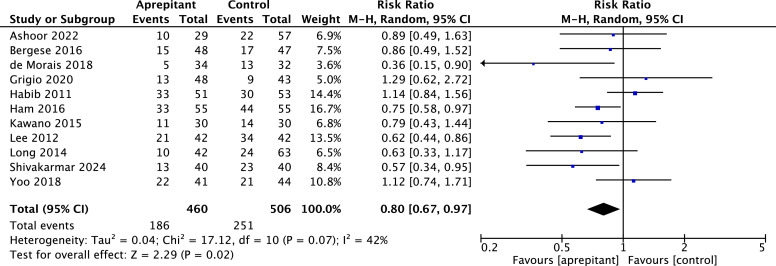
Forest plot showing pooled risk ratio for the incidence of nausea between 0‒24 hours after surgery. Comparison between aprepitant and control; 95 % CI, 95 % Confidence Interval; df, Degrees of freedom; I^2^, Heterogeneity; M-H, random, Mantel-Haenszel random-effects model.

Results from 11 RCTs^20^^,^^22^^,^^25^^,^^28^, ^29^, ^30^^,^^36^^,^^37^^,^^39^^,^^48^^,^^51^ using aprepitant (Fig. 2) yield a statistically significant difference in nausea reduction between the aprepitant and control group from 0 to 24 hours after surgery.

In the period from 0 to 2 hours post-surgery, findings from 10 aprepitant RCTs^18^^,^^20^^,^^22^^,^^25^^,^^28^^,^^29^^,^^32^, ^33^, ^34^^,^^36^ demonstrate a statistically significant decrease in nausea incidence among those in the aprepitant group (RR = 0.70, 95 % CI 0.53 to 0.93; the incidence in the aprepitant group is 102/451, and in the control group is 133/431; I^2^ = 37 %; *p* = 0.12) (Fig. S1).

The funnel plot showed a symmetrical distribution of studies evaluating aprepitant as an intervention drug between 0‒24 hours (Egger test *p* = 0.4862) (Fig. S2), indicating no risk of publication bias. The trim-and-fill analysis sustained the result (Fig. S2.1).

Vomiting (or retching)

**Fig. 3:**
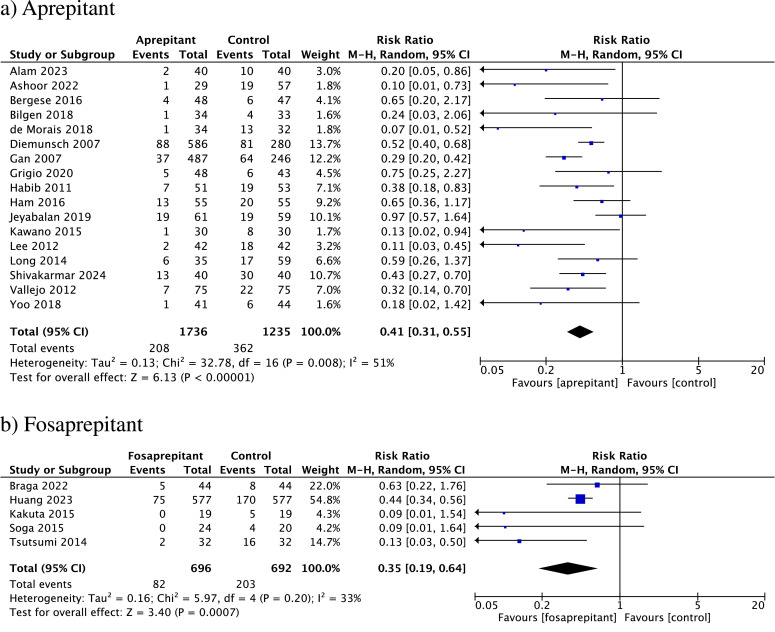
Forest plot showing pooled risk ratio for the incidence of vomiting/retching between 0‒24 hours after surgery; (a) Comparison between aprepitant and control; (b) Comparison between fosaprepitant to control. 95 % CI, 95 % Confidence Interval; df, Degrees of freedom; I^2^, Heterogeneity; M-H, random, Mantel-Haenszel random-effects model.

Meta-analysis revealed a statistically significant reduction in postoperative vomiting (or retching) from 0 to 24 hours and from 0 to 2 hours after surgery when comparing aprepitant or fosaprepitant groups and control group (from 0 to 24 hours after surgery: aprepitant: RR = 0.41, 95 % CI 0.31 to 0.55; I^2^ = 51 %; *p* = 0.008; Fosaprepitant: RR = 0.35, 95 % CI 0.19 to 0.64; I^2^ = 33 %; *p* = 0.20; from 0 to 2 hours after surgery: aprepitant: RR = 0.41, 95 % CI 0.24 to 0.69; I^2^ = 6 %; *p* = 0.39; Fosaprepitant: RR = 0.20, 95 % CI 0.08 to 0.48; I^2^ = 0 %; *p* = 0.98) (Figs. 3a and b and S4).

The authors found asymmetrical distribution representing individual aprepitant studies in the funnel plot from 0‒24 hours (Egger test *p* = 0.0054), suggesting publication bias (Fig. S6). This finding was further supported by the trim-and-fill analysis, which also suggested the presence of missing studies.

Incidence of complete response

**Fig. 4:**
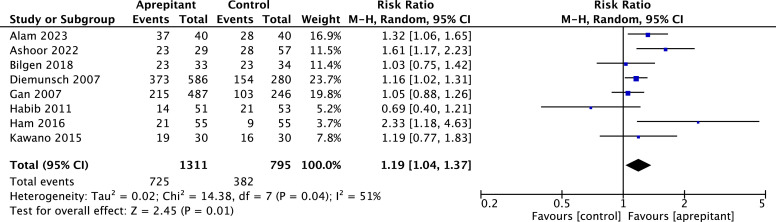
Forest plot showing pooled risk ratio for the incidence of complete response between 0‒24 hours after surgery. Comparison between aprepitant and control; 95 % CI, 95 % Confidence Interval; df, Degrees of freedom; I^2^, Heterogeneity; M-H, Random, Mantel-Haenszel random-effects model.

Results from 8 aprepitant RCTs,^18^^,^^20^^,^^23^^,^^26^^,^^27^^,^^29^^,^^30^^,^^36^ including 2106 participants, yielded a statistically significant difference between aprepitant and control on complete response rate between 0 and 24 hours after surgery (RR = 1.19, 95 % CI 1.04 to 1.37; I^2^ = 51 %; *p* = 0.04) (Fig. 4a).

Postoperative rescue antiemetic use

**Fig. 5:**
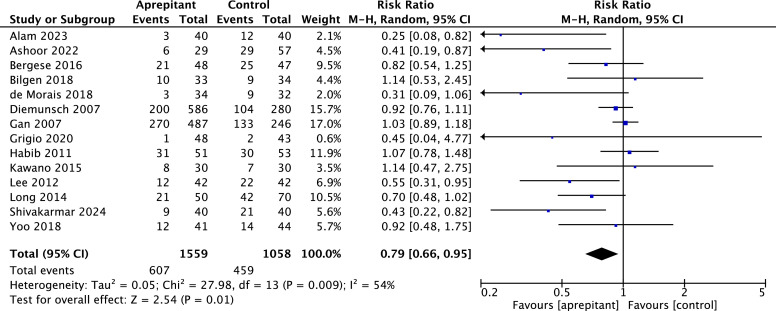
Forest plot showing pooled risk ratio for the incidence of rescue antiemetic use between 0‒24 h after surgery. Comparison between aprepitant and control; 95 % CI, 95 % confidence interval; df, degrees of freedom; I^2^, heterogeneity; M-H, random, Mantel-Haenszel random-effects model.

Results from aprepitant 14 RCTs yielded a statistically significant difference between aprepitant and control in the reduction of postoperative rescue antiemetic use between 0 and 24 hours after surgery (RR = 0.79, 95 % CI 0.66 to 0.95; I^2^ = 54 %; *p* = 0.009; (Fig. 5). However, there were no statistically significant differences from 0 to 2 hours after surgery (Fig. S8).

A funnel plot was made to look for publication bias. An asymmetrical distribution of aprepitant studies was observed for the outcome of rescue antiemetic use between 0‒24 hours, with the Egger test indicating significant asymmetry (*p* = 0.0004; Fig. S9). This suggests a risk of publication bias, which was further supported by the trim-and-fill analysis (Fig. S9.1).

Adverse effects

Due to the high variability in adverse effects, meta-analysis was possible for only three outcomes, each with five or fewer studies. No significant differences were found in overall adverse effects, headache, or dizziness between the aprepitant and control groups within 24 hours post-surgery. Specifically, headache was reported in 41.8 % of patients receiving aprepitant and in 40.5 % of the control group, while dizziness occurred in 21.7 % of patients in the aprepitant group and in 20.7 % of the control group. A post-hoc analysis also showed no significant difference in headache between groups at 48 hours.

### Subgroup analysis

In the subgroup analysis for the type of anaesthesia, participants who received aprepitant and inhalational anaesthesia technique also showed a reduction in the incidence of nausea and vomiting (or retching) from 0 to 24 hours and from 0 to 2 hours after surgery. Additionally, from 0 to 24 hours after surgery, there was also a statistically significant reduction in the use of rescue antiemetics and a higher complete response rate.

In the aprepitant RCTs, multitherapy (aprepitant plus a combination of other antiemetics) showed a reduction in the incidence of nausea and vomiting (or retching) from 0 and 24 hours, and from 0 to 2 hours after surgery. Moreover, patients who received monotherapy (aprepitant as solely antiemetic) only showed a statistically significant reduction in the incidence of vomiting (or retching) from 0 to 24 hours after surgery.

In the subgroup analysis for different doses of aprepitant, aprepitant 80 mg showed a reduction in the incidence of nausea and vomiting (or retching) from 0 to 24 hours and from 0 to 2 hours after surgery. There was also a statistically significant reduction in the use of postoperative rescue antiemetics from 0 to 24 hours after surgery. On the other hand, aprepitant 40 mg reduced only the incidence of vomiting from 0 to 24 hours after surgery.

The results showed that aprepitant reduces the incidence of nausea and vomiting from 0 to 24 hours and from 0 to 2 hours after surgery in studies evaluating the pure effect (studies that compared aprepitant versus placebo, or aprepitant plus standard antiemetics versus standard antiemetics) (Figs. S30‒33) In addition, the “pure effect” of aprepitant showed a statistically significant reduction in the use of postoperative rescue antiemetic from 0 to 24 hours after surgery (Fig. S37).

In studies comparing aprepitant with other antiemetics, there was a statistically significant difference only in the reduction of the incidence of vomiting from 0 to 24 hours and from 0 to 2 hours after surgery (Figs. S34 and 35).

Women who received aprepitant had a statistically significantly lower incidence of nausea (RR = 0.76, 95 % CI 0.61 to 0.96; I^2^ = 38 %, *p* = 0.14), and vomiting (RR = 0.41, 95 % CI 0.22 to 0.77; I^2^ = 41 %, *p* = 0.007) within both 0 to 24 hours and 0 to 2 hours after surgery. Additionally, women who received aprepitant also had a statistically significant lower need for rescue antiemetic medication (RR = 0.69, 95 % CI 0.53 to 0.90; I^2^ = 0 %, *p* = 0.48) during the 0 to 24-hour postoperative period.

The results of subgroup meta-analysis of pooled studies evaluating the use of aprepitant in high-risk surgeries for PONV showed that there was a benefit from aprepitant in reducing the incidence of nausea, vomiting, and the consumption of postoperative rescue antiemetics from 0 to 24 hours after surgery.

On the other hand, in the results subgroup analysis of low-risk surgeries for PONV, aprepitant only proved beneficial in reducing the incidence of vomiting (RR = 0.48, 95 % CI 0.28 to 0.80; I^2^ = 50 %, *p* = 0.06) from 0 to 24 hours after surgery.

No subgroup meta-analysis was carried out on fosaprepitant studies because all subgroups contained fewer than 5 studies. The meta-analyses summary of the subgroups can be seen in the Supplementary Online Material (Tables S10‒52).

### Meta-regression analysis

Three outcomes met the criteria for meta-regression, each including more than ten trials. In the analysis of nausea incidence within 0–24 h (Box S1), residual heterogeneity remained moderate after meta-regression, which I^2^ changed only slightly from 42.15 % to 39.72 %. Additionally, the test of moderators was not statistically significant (QM(5) = 4.95; *p* = 0.42), indicating that the included covariates did not explain a significant portion of the between-study variability. Despite this, the moderator ‘high-risk surgery’ was significantly associated with a lower risk of nausea compared to ‘low-risk surgery’ (RR = 0.59; *p* = 0.034).

For the outcome of vomiting within 0‒24 hours (Box S2), the meta-regression model was not statistically significant (QM(5) = 4.16; *p* = 0.53), and none of the included moderators demonstrated a significant effect.

In contrast, the meta-regression analysis for rescue antiemetic use within 0–24 h (Box S3) yielded a significant model (QM(5) = 14.32; *p* = 0.014), which accounted for approximately 93.2 % of the variance across studies. Among the moderators, only drug dose emerged as a statistically significant predictor (estimate = −0.019; *p* = 0.028), indicating that higher doses were associated with reduced rescue antiemetic use. Residual heterogeneity was low (I^2^ = 8.93 %; *p* = 0.19), suggesting that the model effectively captured key sources of variability.

### Sensitivity analysis

Sensitivity analyses were performed, including only studies with a low risk of bias. In analysis with aprepitant studies, the incidence of nausea (five studies)^22^^,^^30^^,^^36^^,^^37^^,^^51^ and vomiting (eight studies)^22^^,^^26^^,^^27^^,^^30^^,^^36^^,^^37^^,^^45^^,^^51^ reduced statistically significantly from 0 to 24 hours after surgery (Figs. S53‒57).

## Discussion

The main findings of this systematic review are that aprepitant significantly reduces the incidence of nausea, vomiting from 0 to 24 hours and from 0 to 2 hours after surgery. In addition, aprepitant reduces the use of postoperative rescue antiemetics and increases the complete response rate from 0 to 24 hours after surgery. Aprepitant has been shown to be an effective antiemetic in patients receiving inhalational anaesthesia, in women, and in high-risk surgeries for PONV, reducing the incidence of nausea, vomiting and the use of rescue antiemetics. Sole use of aprepitant and combination of aprepitant plus other antiemetics are effective for the prevention of PONV. Aprepitant 80 mg showed to be more effective than 40 mg as it reduces the incidence of nausea, vomiting and the use of postoperative antiemetics from 0 to 24 hours after surgery, while aprepitant 40 mg only reduces the incidence of vomiting. The present study and one other review^53^ included a few studies that evaluated doses of 125 mg of aprepitant. Therefore, it is not possible to know whether there are more benefits with doses of 125 mg of aprepitant. Fosaprepitant showed a reduction in the incidence of vomiting from 0 to 24 hours and from 0 to 2 hours after surgery. Although in the meta-regression analysis of nausea incidence 0‒24 hours in high-risk procedures were related to reduced PONV, given the lack of a significant overall model and the limited reduction in heterogeneity, this isolated finding should be interpreted with caution. A possible explanation for the counterintuitive direction of effect is the use of more antiemetics in these cases, following PONV prevention protocols. Additionally, unmeasured confounders could influence this result.

The result of this meta-analysis is in accordance with previous systematic reviews^11^^,^^54^^,^^55^ that showed aprepitant as an effective antiemetic to reduce the incidence of nausea, vomiting and rescue antiemetic use from 0 to 24 hours after surgery.^11^^,^^55^ Only one previous systematic review showed the benefit of aprepitant in reducing the incidence of nausea and vomiting from 0 to 2 hours^54^ after surgery. In addition, like other systematic review,^7^^,^^11^^,^^12^^,^^53^, ^54^, ^55^, ^56^ this study showed that aprepitant 80 mg significantly reduced the incidence of nausea and vomiting (or retching) from 0 and 24 hours, and from 0 to 2 hours after surgery.

The difference in the results of the meta-analysis with previous systematic reviews is due to differences in evaluation times,^11^^,^^12^^,^^53^^,^^55^ doses,^54^^,^^55^ definition of the outcome,^7^ type of surgery,^54^ the number of studies included,^11^^,^^12^^,^^53^^,^^55^^,^^56^ and avoiding repetition of studies within the same meta-analysis.^53^

Regarding fosaprepitant, the meta-analysis showed a statistically significant reduction only in the incidence of postoperative vomiting from 0 to 24 hours after surgery. Fosaprepitant 115 mg is considered bioequivalent to oral aprepitant 125 mg.^10^ Despite this equivalence, only vomiting – not nausea – was significantly reduced. This suggests that route of administration or pharmacokinetic differences, such as timing of peak concentration, may influence clinical efficacy.

Publication bias could have influenced the outcome of rescue antiemetic use and vomiting from 0 to 24 hours after surgery by skewing the results. In the aprepitant studies, there is a higher chance that studies with positive results were more likely to be published because all published systematic reviews report benefit from aprepitant in reducing the incidence of vomiting, and studies with negative results were given less importance.

No serious adverse events were linked to aprepitant in the Cochrane review,^7^ and this finding was consistent with our own results when examining any side effects. The slight differences in the incidence of headache and dizziness between the aprepitant and control groups further support the comparable safety profile of aprepitant. Other reviews^7^^,^^11^^,^^12^^,^^54^ did not gather estimates and reported no consistent side effects from using aprepitant.

In the sensitivity analysis, the authors excluded studies of poor quality to improve the validity of the present study, providing a more accurate assessment of the effectiveness of aprepitant and fosaprepitant for PONV. No previous review has conducted a sensitivity analysis evaluating only studies with a low risk of bias. The authors confirmed these results in the aprepitant studies with respect to a significant reduction in the incidence of nausea and vomiting from 0 to 24 hours after surgery. In addition, the included studies with fosaprepitant also showed a decrease in the incidence of vomiting and rescue antiemetic use from 0 to 24 hours after surgery.

In addition to clinical efficacy, cost-effectiveness is a key factor in the adoption of antiemetic strategies. Aprepitant and fosaprepitant are generally more expensive than commonly used agents such as dexamethasone or ondansetron. While acquisition costs are higher, aprepitant’s superior vomiting reduction (RR = 0.41) could offset costs through reduced rescue medication use and shorter recovery, though formal cost-utility analyses are lacking. Additionally, previous meta-analyses have shown that NK1 receptor antagonists, such as aprepitant, achieve significantly greater reductions in postoperative vomiting compared to other antiemetics, with risk ratios as low as 0.26 when compared to other antiemetics.^7^

### Strengths and limitations

The authors did an extensive literature search, and the pooled number of participants was larger than in other systematic reviews. The present study included 18 more RCTs (with 2942 extra participants) in comparison with the last published.^11^ In addition, the authors did not group the evaluation times of the studies within the first 24 hours after surgery, but rather grouped the evaluation times of the study considering the evaluation time actually studied. The symptoms assessed in this study were isolated symptoms, and were not grouped into nausea and vomiting, since previous studies differed in the way the symptoms nausea and vomiting were presented, such as: nausea or vomiting, nausea and vomiting, nausea and/or vomiting. Lastly, the authors performed sensitivity analyses to explore the impact and influence of trials with a high risk of bias on the overall results, ensuring that the conclusions are reliable and grounded in critically evaluating the evidence.

This study has some limitations. The authors have combined any definition of complete response into just one outcome, and comparability between the different definitions can lead to misinterpretations. Moreover, the authors could not generalize the results to males as the majority of participants were female.

### Future research

The present review has several implications for future research. First, although most included participants were female, future RCTs should aim to recruit a more balanced population with adequate male representation to enhance the generalizability of findings across sexes. Second, the authors endorse the call for standardized reporting of adverse effects in future RCTs, which would enable more reliable safety assessments and facilitate future meta-analysis focused specifically on adverse events. Third, given the limited number of studies evaluating fosaprepitant, the authors emphasize the need for more robust, well-designed RCTs assessing its efficacy and safety for PONV prophylaxis. Finally, further studies are warranted to assess their cost-effectiveness in a variety of surgical settings and patient populations.

## Conclusion

The present systematic review showed that aprepitant reduces the incidence of postoperative nausea, vomiting, and the use of rescue antiemetics and increases the complete response rate among adult participants from 0 to 24 hours after surgery. Fosaprepitant reduces the incidence of vomiting from 0 to 24 hours after surgery.

The authors recommend prophylactic aprepitant or fosaprepitant to reduce vomiting in the first 24 hours after surgery. Moreover, aprepitant is also recommended to reduce the use of rescue antiemetics and increase the cases of complete response in the first 24 hours after surgery.

Future studies should aim to include more male participants, standardize the reporting of adverse effects, and further investigate the role of fosaprepitant through additional high-quality RCTs.

## Funding

This research did not receive any specific grant from funding agencies in the public, commercial, or not-for-profit sectors.

## Authors’ contributions

Thiago Ramos Grigio: Conceptualization; methodology; search strategy; formal analysis; data curation; interpretation of data; writing-original draft; visualization.

Hans Timmerman: Conceptualization; methodology; formal analysis; data curation, interpretation of data; writing-review and editing; supervision; visualization.

Natanael Pietroski dos Santos: Formal analysis; data curation; interpretation of data.

José Eduardo Guimarães Pereira: Interpretation of data; writing, review, and editing.

Angela Maria Sousa: Interpretation of data; writing-review and editing; supervision. andré paul wolff: conceptualization; methodology; interpretation of data; writing-review and editing; supervision.

## Declaration of competing interest

The authors declare no conflicts of interest.

## Appendix Supplementary materials

Image, application 1
