# Supplementary material for: Aprepitant and fosaprepitant as a prophylactic antiemetic for preventing postoperative nausea and vomiting after general anaesthesia: a systematic review and meta-analysis
Source: Clinics (Sao Paulo). 2025 Oct 15;80:100783. doi: 10.1016/j.clinsp.2025.100783 (PMC12550584; doi:10.1016/j.clinsp.2025.100783)
Supplement: Supplementary file 1 [file mmc1.docx]

CLINICS-D-25-00124_Supplementary Material

**Appendix 1_SEARCH STRATEGY**

| **PUBMED** |
| --- |
| ("Aprepitant"[Mesh] OR "fosaprepitant" [Supplementary Concept] OR emend[tiab] OR fosaprepitant[tiab] OR 758298[tiab] ORl758298[tiab] OR “mk 0517” [tiab] OR mk0517[tiab] OR “ono 7847”[tiab] OR ono7847[tiab] OR proemend[tiab] OR  “L 785,298”[tiab] OR L785,298 OR "1-(3,5-bis(trifluoromethyl)phenyl)ethanol" [Supplementary Concept] OR 3,5-BTPE[tiab]) OR aprepitant[tiab] OR “MK 869”[tiab] OR “MK 0869”[tiab] OR MK0869[tiab] OR MK869[tiab] OR “methyl-morpholine*”[tiab] OR methylmorpholine*[tiab] OR “MK 0517”[tiab] OR MK0517[tiab] OR Emend[tiab] OR “L 754030”[tiab] OR L754030[tiab] OR “L 754,030”[tiab] OR aponvie[tiab] OR aprepilor[tiab] OR atanto[tiab] OR cinvanti[tiab] OR inepitant[tiab] OR “ht 001”[tiab] OR ht001[tiab] OR “htx 019”[tiab] OR htx019[tiab] OR lorpitan[tiab] OR ono7436[tiab] OR weg232[tiab] OR "Neurokinin-1 Receptor Antagonists"[Mesh] OR ((“neurokinin-1 receptor*”[tiab] OR “tachykinin Receptor 1”[tiab] OR “Substance P Receptor*”[tiab] OR NK1R*[tiab] OR NK1[tiab]) AND (antagonist*[tiab] OR blocker*[tiab])) OR "Neurokinin-1 Receptor Antagonists" [Pharmacological Action] |
| AND |
| ("Postoperative Nausea and Vomiting"[Mesh] OR ponv[tiab] OR  ((postoperat*[tiab] OR “post-operat*”[tiab] OR “post-procedur*”[tiab] OR postprocedur*[tiab] OR postsurg*[tiab] OR postdischarge[tiab] OR “post-discharge”[tiab] OR anesth*[tiab] OR anaesth*[tiab] OR postanesth*[tiab] OR postanaesth*[tiab] OR surg*[tiab]) AND ("Nausea"[Mesh] OR "Vomiting"[Mesh] OR vomit*[tiab] OR nause*[tiab] OR sick*[tiab] OR retch*[tiab] OR emes*[tiab]))) |
|  |
| **EMBASE** |
| ('aprepitant'/exp OR 'fosaprepitant'/exp OR emend:ti,ab,kw OR fosaprepitant:ti,ab,kw,tn OR ivemendl:ti,ab,kw OR 758298:ti,ab,kw OR l758298:ti,ab,kw OR 'mk 0517':ti,ab,kw OR mk0517:ti,ab,kw OR mk517:ti,ab,kw OR nvk016:ti,ab,kw OR 'ono 7847':ti,ab,kw OR ono7847:ti,ab,kw OR proemend:ti,ab,kw OR 'L 785,298':ti,ab,kw OR ‘L785,298’:ti,ab,kw OR ‘1-(3,5-bis(trifluoromethyl)phenyl)ethanol’:tn,ti,ab,kw OR 3,5-BTPE:ti,ab,kw OR aprepitant:ti,ab,kw,tn OR 'MK 869':ti,ab,kw OR 'MK 0869':ti,ab,kw OR MK0869:ti,ab,kw OR MK869:ti,ab,kw OR ‘methyl-morpholine*’:ti,ab,kw OR methylmorpholine*:ti,ab,kw OR 'MK 0517':ti,ab,kw OR MK0517:ti,ab,kw OR Emend:ti,ab,kw OR 'L 754030':ti,ab,kw OR L754030:ti,ab,kw OR 'L 754,030':ti,ab,kw OR aponvie:ti,ab,kw OR aprepilor:ti,ab,kw OR atanto:ti,ab,kw OR cinvanti:ti,ab,kw OR inepitant:ti,ab,kw OR 'ht 001':ti,ab,kw OR ht001:ti,ab,kw OR 'htx 019':ti,ab,kw OR htx019:ti,ab,kw OR lorpitan:ti,ab,kw OR ono7436:ti,ab,kw OR weg232:ti,ab,kw OR 'neurokinin 1 receptor antagonist'/exp OR (('neurokinin-1 receptor*':ti,ab,kw OR 'tachykinin Receptor 1':ti,ab,kw OR 'Substance P Receptor*':ti,ab,kw OR NK1R*:ti,ab,kw OR NK1:ti,ab,kw) AND (antagonist*:ti,ab,kw OR blocker*:ti,ab))) |
| AND |
| ('postoperative nausea and vomiting'/exp OR ponv:ti,ab,kw OR |
| ((postoperat*:ti,ab,kw OR post-operat*:ti,ab,kw OR post-procedur*:ti,ab,kw OR postprocedur*:ti,ab,kw OR postsurg*:ti,ab,kw OR postdischarge:ti,ab,kw OR post-discharge:ti,ab,kw OR anesth*:ti,ab,kw OR anaesth*:ti,ab,kw OR postanesth*:ti,ab,kw OR postanaesth*:ti,ab,kw OR surg*:ti,ab,kw) AND ('nausea'/exp OR 'vomiting'/exp OR vomit*:ti,ab,kw OR nause*:ti,ab,kw OR sick*:ti,ab,kw OR retch*:ti,ab,kw OR emes*:ti,ab,kw))) |
| NOT [conference abstract]/lim |
|  |
| **WoS** |
| TS=(aprepitant OR fosaprepitant OR emend OR ivemendl OR 758298 OR l758298 OR "mk 0517" OR mk0517 OR mk517 OR nvk016 OR "ono 7847" OR ono7847 OR proemend OR "L 785,298" OR L785,298 OR "1-(3,5-bis(trifluoromethyl)phenyl)ethanol" OR 3,5-BTPE OR “MK 869” OR “MK 0869” OR MK0869 OR MK869 OR “methyl-morpholine*” OR methylmorpholine* OR “MK 0517” OR MK0517 OR Emend OR “L 754030” OR L754030 OR “L 54,030” OR aponvie OR aprepilor OR atanto OR cinvanti OR inepitant OR “ht 001” OR ht001 OR “htx 019” OR htx019 OR lorpitan OR ono7436 OR weg232 OR ((“neurokinin-1 receptor*” OR “tachykinin receptor 1” OR “Substance P Receptor*” OR NK1R* OR NK1) AND (antagonist* OR blocker*))) |
| AND |
| TS=("Postoperative Nausea and Vomiting" OR ponv OR |
| ((postoperat* OR “post-operat*” OR “post-procedur*” OR postprocedur* OR postsurg* OR postdischarge OR “post-discharge” OR anesth* OR anaesth* OR postanesth* OR postanaesth* OR surg*) AND (Nausea OR Vomiting OR vomit* OR nause* OR sick* OR retch* OR emes*))) |
|  |
| **COCHRANE CENTRAL REGISTER OF CONTROLLED TRIALS (CENTRAL)** |
| ([mh Aprepitant] OR [mh "Fosaprepitant"] OR fosaprepitant:ti,ab,kw OR emend:ti,ab,kw OR ivemendl:ti,ab,kw OR 758298:ti,ab,kw OR l758298:ti,ab,kw OR "mk 0517":ti,ab,kw OR mk0517:ti,ab,kw OR mk517:ti,ab,kw OR nvk016:ti,ab,kw OR "ono 7847":ti,ab,kw OR ono7847:ti,ab,kw OR proemend:ti,ab,kw OR "L 785,298":ti,ab,kw OR L785,298:ti,ab,kw OR aprepitant:ti,ab,kw OR "MK 869":ti,ab,kw OR "MK 0869":ti,ab,kw OR MK0869:ti,ab,kw OR MK869:ti,ab,kw OR "methyl-morpholine*":ti,ab,kw OR methylmorpholine*:ti,ab,kw OR "MK 0517":ti,ab,kw OR MK0517:ti,ab,kw OR Emend:ti,ab,kw OR "L 754030":ti,ab,kw OR L754030:ti,ab,kw OR "L 754,030":ti,ab,kw OR aponvie:ti,ab,kw OR aprepilor:ti,ab,kw OR atanto:ti,ab,kw OR cinvanti:ti,ab,kw OR inepitant:ti,ab,kw OR "ht 001":ti,ab,kw OR ht001:ti,ab,kw OR "htx 019":ti,ab,kw OR htx019:ti,ab,kw OR lorpitan:ti,ab,kw OR ono7436:ti,ab,kw OR weg232:ti,ab,kw OR [mh "Neurokinin-1 Receptor Antagonists"] OR ((("neurokinin-1" NEXT receptor*):ti,ab,kw OR "tachykinin Receptor 1":ti,ab,kw OR ("Substance P" NEXT Receptor*):ti,ab,kw OR NK1R*:ti,ab,kw OR NK1:ti,ab,kw) AND (antagonist*:ti,ab,kw OR blocker*:ti,ab,kw))) |
| AND |
| ([mh "Postoperative Nausea and Vomiting"] OR ponv:ti,ab,kw OR ((postoperat*:ti,ab,kw OR "post-operat*":ti,ab,kw OR "post-procedur*":ti,ab,kw OR postprocedur*:ti,ab,kw OR postsurg*:ti,ab,kw OR postdischarge:ti,ab,kw OR "post-discharge":ti,ab,kw OR anesth*:ti,ab,kw OR anaesth*:ti,ab,kw OR postanesth*:ti,ab,kw OR postanaesth*:ti,ab,kw OR surg*:ti,ab,kw) AND ([mh Nausea] OR [mh Vomiting] OR vomit*:ti,ab,kw OR nause*:ti,ab,kw OR sick*:ti,ab,kw OR retch*:ti,ab,kw OR emes*:ti,ab,kw))) |

**Supplementary Material**

**Table S1** Quality assessment of controlled intervention studies

| **Author, Year** | **Alam M., 2023** | **Alonso-Damián E.R., 2012** | **Ashoor T.M., 2022** | **Atsuta J., 2017** | **Bergese S.D., 2016** | **Bilgen S., 2018** | **Braga E.L.C., 2022** | **de Morais L.C., 2018** | **Diemunsch P., 2007** | **Gan T.J., 2007** | **Gökdemir B.N., 2024** | **Grigio T.R., 2020** | **Habib A.S., 2011** | **Ham S.Y., 2016** | **Huang Q., 2023** | **Jeyabalan S., 2019** | **Jung W.S., 2013** |
| --- | --- | --- | --- | --- | --- | --- | --- | --- | --- | --- | --- | --- | --- | --- | --- | --- | --- |
| 1. Was the study described as a randomized, a randomized trial, a randomized clinical trial, or an RCT? | YES | YES | YES | YES | YES | YES | YES | YES | YES | YES | YES | YES | YES | YES | YES | YES | YES |
| 2. Was the method of randomization adequate (i.e., use of randomly generated assignment)? | YES | NR | YES | YES | YES | YES | YES | YES | YES | YES | YES | YES | YES | YES | YES | YES | YES |
| 3. Was the treatment allocation concealed (so that assignments could not be predicted)? | YES | NR | YES | YES | YES | CD | YES | YES | YES | YES | CD | YES | YES | YES | YES | YES | YES |
| 4. Were study participants and providers blinded to treatment group assignment? | YES | NR | YES | YES | YES | YES | YES | YES | YES | YES | CD | YES | YES | YES | YES | YES | YES |
| 5. Were the people assessing the outcomes blinded to the participants' group assignments? | YES | NR | YES | YES | YES | YES | YES | YES | YES | NR | CD | YES | YES | YES | YES | YES | YES |
| 6. Were the groups similar at baseline on important characteristics that could affect outcomes (e.g., demographics, risk factors, co-morbid conditions)? | YES | NO | YES | YES | YES | YES | YES | CD | YES | YES | YES | YES | YES | YES | YES | YES | YES |
| 7. Was the overall drop-out rate from the study at endpoint 20% or lower of the number allocated to treatment? | YES | YES | YES | YES | YES | YES | YES | NO | YES | YES | YES | YES | YES | YES | YES | YES | NR |
| 8. Was the differential drop-out rate (between treatment groups) at endpoint 15 percentage points or lower? | YES | YES | YES | YES | CD | YES | YES | YES | YES | YES | YES | YES | YES | YES | YES | YES | NR |
| 9. Was there high adherence to the intervention protocols for each treatment group? | YES | NR | YES | YES | YES | YES | YES | YES | YES | YES | YES | YES | YES | YES | YES | YES | NR |
| 10. Were other interventions avoided or similar in the groups (e.g., similar background treatments)? | YES | YES | YES | YES | YES | YES | YES | YES | YES | YES | YES | YES | YES | YES | YES | YES | YES |
| 11. Were outcomes assessed using valid and reliable measures, implemented consistently across all study participants? | YES | CD | YES | YES | YES | YES | YES | YES | YES | YES | YES | YES | YES | YES | YES | YES | YES |
| 12. Did the authors report that the sample size was sufficiently large to be able to detect a difference in the main outcome between groups with at least 80% power? | YES | NO | YES | YES | YES | YES | YES | YES | YES | YES | YES | NO | YES | YES | YES | YES | YES |
| 13. Were outcomes reported or subgroups analyzed prespecified (i.e., identified before analyses were conducted)? | YES | NO | YES | YES | YES | YES | YES | YES | YES | YES | YES | YES | YES | YES | YES | YES | YES |
| 14. Were all randomized participants analyzed in the group to which they were originally assigned, i.e., did they use an intention-to-treat analysis? | NO | YES | NO | NO | YES | NO | YES | YES | YES | YES | YES | NO | NO | YES | YES | NO | YES |
| POOR QUALITY? | YES | NO | YES | YES | NO | YES | NO | YES | NO | NO | NO | YES | YES | NO | NO | YES | NO |

**Table S1** (continued)

| **Author, Year** | **Kakuta N., 2011** | **Kakuta N., 2015** | **Kawano H., 2015** | **Lee SJ, 2012** | **Lim C.S., 2013** | **Long J.B, 2014** | **Moon H.Y., 2014** | **Ortiz E., 2024** | **Patro G.S.P., 2022** | **Shivakarmar G., 2024** | **Sinha A.C., 2014** | **Soga T., 2015** | **Thanuja I.L., 2021** | **Tsutsumi Y.M., 2014** | **Vallejo M.C., 2012** | **Wajid Q.A., 2022** | **Yeo G., 2018** | **Yoo J.H., 2018** |
| --- | --- | --- | --- | --- | --- | --- | --- | --- | --- | --- | --- | --- | --- | --- | --- | --- | --- | --- |
| 1. Was the study described as a randomized, a randomized trial, a randomized clinical trial, or an RCT? | YES | YES | YES | YES | YES | YES | YES | YES | YES | YES | YES | YES | YES | YES | YES | YES | YES | YES |
| 2. Was the method of randomization adequate (i.e., use of randomly generated assignment)? | NR | YES | YES | YES | YES | YES | YES | YES | YES | YES | YES | YES | YES | YES | YES | YES | YES | YES |
| 3. Was the treatment allocation concealed (so that assignments could not be predicted)? | NR | CD | YES | CD | CD | NR | YES | NO | YES | CD | NR | YES | YES | YES | YES | NR | YES | NR |
| 4. Were study participants and providers blinded to treatment group assignment? | NR | YES | NR | NO | NR | CD | CD | CD | YES | NO | YES | YES | YES | YES | YES | NR | YES | CD |
| 5. Were the people assessing the outcomes blinded to the participants' group assignments? | NR | YES | YES | NO | NR | NR | YES | NR | YES | NO | YES | YES | YES | YES | YES | NR | YES | CD |
| 6. Were the groups similar at baseline on important characteristics that could affect outcomes (e.g., demographics, risk factors, co-morbid conditions)? | YES | YES | YES | YES | YES | CD | YES | YES | YES | YES | YES | YES | CD | YES | YES | NR | YES | YES |
| 7. Was the overall drop-out rate from the study at endpoint 20% or lower of the number allocated to treatment? | YES | YES | NR | YES | YES | NO | YES | YES | YES | YES | YES | YES | YES | YES | YES | NR | YES | YES |
| 8. Was the differential drop-out rate (between treatment groups) at endpoint 15 percentage points or lower? | YES | YES | NR | YES | YES | NO | YES | YES | YES | YES | YES | YES | YES | YES | YES | NR | YES | YES |
| 9. Was there high adherence to the intervention protocols for each treatment group? | YES | YES | YES | YES | YES | CD | YES | YES | YES | YES | YES | YES | YES | YES | YES | NR | YES | NO |
| 10. Were other interventions avoided or similar in the groups (e.g., similar background treatments)? | YES | YES | YES | YES | YES | NR | YES | YES | YES | NR | YES | YES | YES | YES | YES | YES | YES | YES |
| 11. Were outcomes assessed using valid and reliable measures, implemented consistently across all study participants? | NO | YES | YES | YES | YES | YES | YES | YES | YES | YES | YES | YES | YES | YES | YES | NR | YES | YES |
| 12. Did the authors report that the sample size was sufficiently large to be able to detect a difference in the main outcome between groups with at least 80% power? | NO | YES | YES | YES | YES | NO | YES | YES | YES | YES | YES | YES | YES | YES | YES | YES | YES | YES |
| 13. Were outcomes reported or subgroups analyzed prespecified (i.e., identified before analyses were conducted)? | NO | YES | YES | YES | YES | YES | YES | YES | YES | YES | YES | YES | YES | YES | YES | YES | YES | YES |
| 14. Were all randomized participants analyzed in the group to which they were originally assigned, i.e., did they use an intention-to-treat analysis? | NO | NO | YES | YES | NO | CD | NO | YES | YES | YES | YES | YES | NO | YES | YES | NR | NO | NO |
| POOR QUALITY? | YES | YES | NO | NO | YES | YES | YES | NO | NO | NO | NO | NO | YES | NO | NO | NO | YES | YES |

CD, cannot determine; NR, not reported.

**NAUSEA**


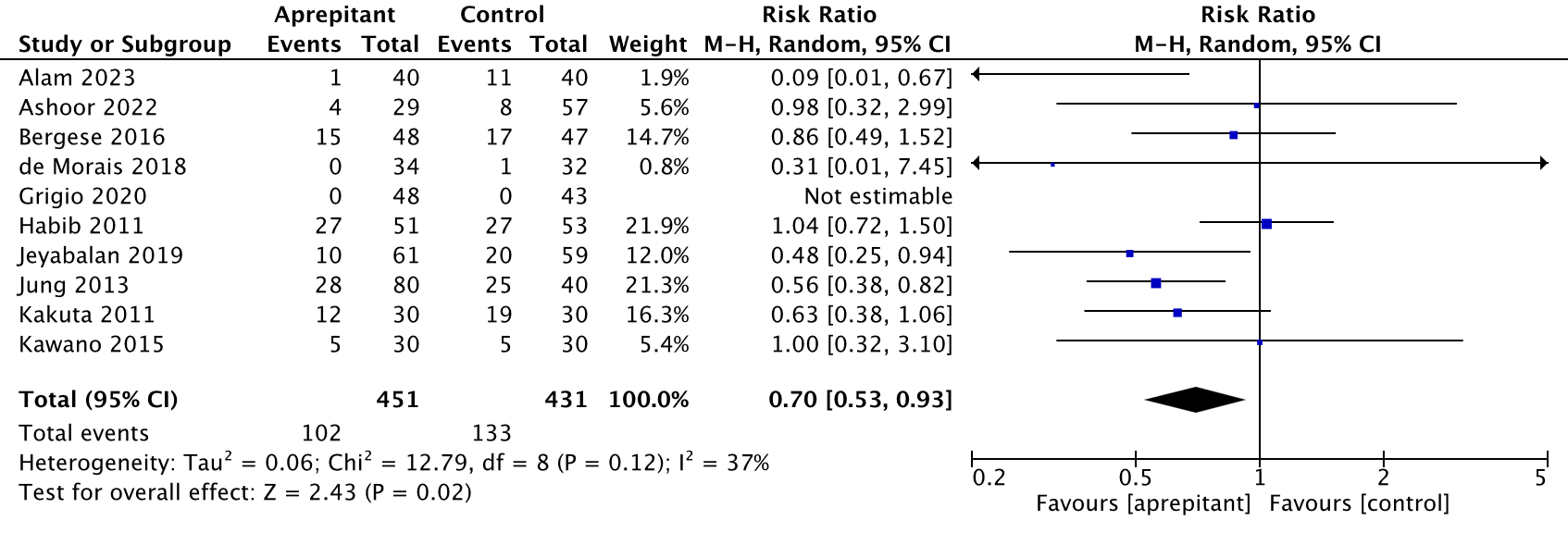


**Figure S1** Forest plot showing pooled risk ratio for the incidence of nausea between 0-2 hours after surgery. Comparison between aprepitant and control; 95%CI, 95% confidence interval; df; degrees of freedom; I^2^, heterogeneity; M-H, random, Mantel-Haenszel random-effects model.

**Box S1** Meta-regression for the incidence of nausea between 0-24 hours after surgery. Comparison between aprepitant and control including the following moderators: type of surgery (cir), anesthesia type (anest), drug dose (dose), and monotherapy (mono).

| Mixed-Effects Model (k = 11; tau^2 estimator: REML)  logLik deviance AIC BIC AICc  -1.5857 3.1714 17.1714 14.4375 129.1714  tau^2 (estimated amount of residual heterogeneity): 0.0349 (SE = 0.0573)  tau (square root of estimated tau^2 value): 0.1869  I^2 (residual heterogeneity / unaccounted variability): 39.72%  H^2 (unaccounted variability / sampling variability): 1.66  R^2 (amount of heterogeneity accounted for): 2.43%  Test for Residual Heterogeneity:  QE(df = 5) = 8.2873, p-val = 0.1411  Test of Moderators (coefficients 2:6):  QM(df = 5) = 4.9526, p-val = 0.4217  Model Results:  estimate se zval pval ci.lb ci.ub  intrcpt -0.6896 0.8279 -0.8329 0.4049 -2.3123 0.9331  cirHigh risk -0.5299 0.2502 -2.1179 0.0342 -1.0204 -0.0395 *  cirMixed/Unknown -0.5376 0.4715 -1.1402 0.2542 -1.4617 0.3865  anest 0.4689 0.5042 0.9300 0.3523 -0.5193 1.4572  dose 0.0080 0.0097 0.8209 0.4117 -0.0111 0.0270  mono -0.1410 0.2664 -0.5290 0.5968 -0.6632 0.3813  ---  Signif. codes: 0 ‘***’ 0.001 ‘**’ 0.01 ‘*’ 0.05 ‘.’ 0.1 ‘ ’ 1 |
| --- |


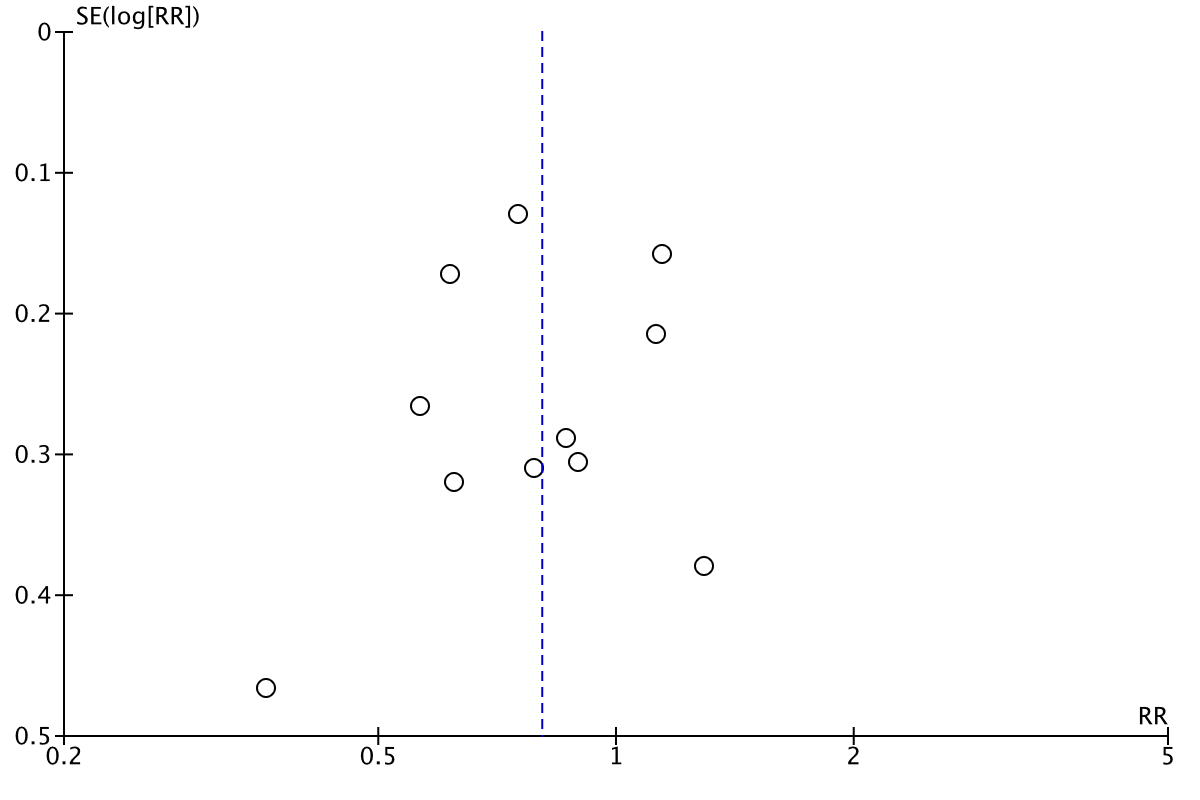


**Figure S2** Funnel plot evaluating publication bias for incidence of nausea between 0-24 hours (aprepitant). Dotted blue line, overall effect; Circles, studies; SE(Log[RR]), standard error (log [risk ratio]); RR, risk ratio.

**
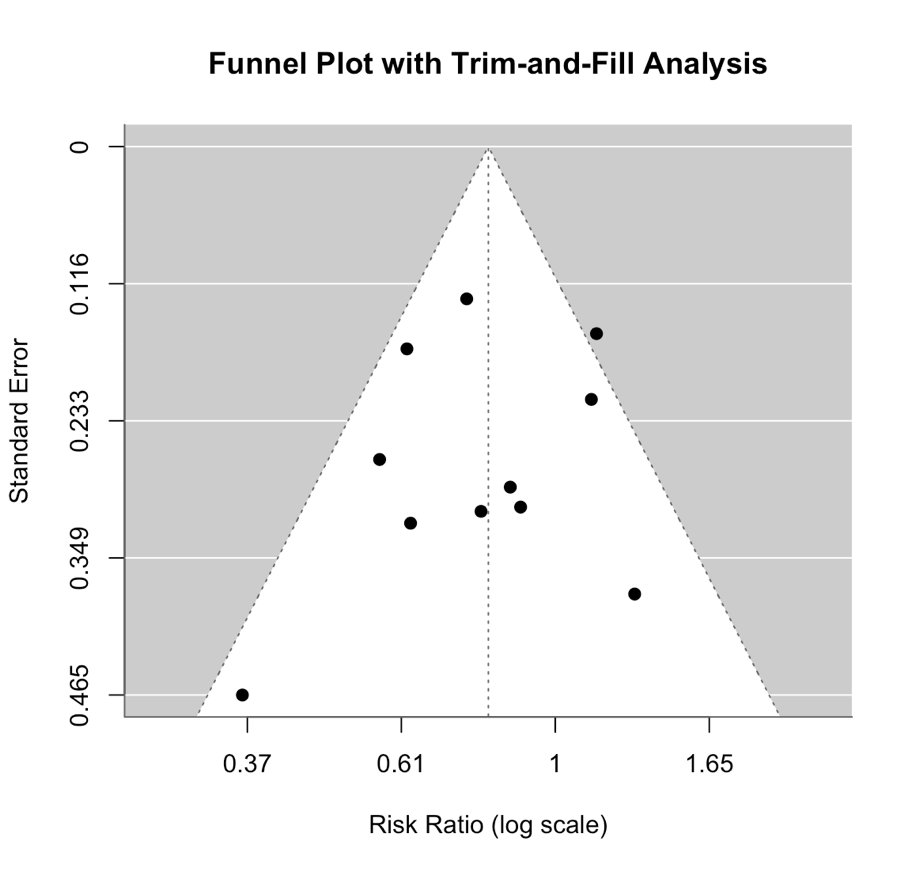
**

**Figure S2.1** Funnel plot with trim-and-fill analysis evaluating publication bias for incidence of nausea between 0-24 hours (aprepitant). Black circles: included studies. There were no estimated missing studies on the right side.


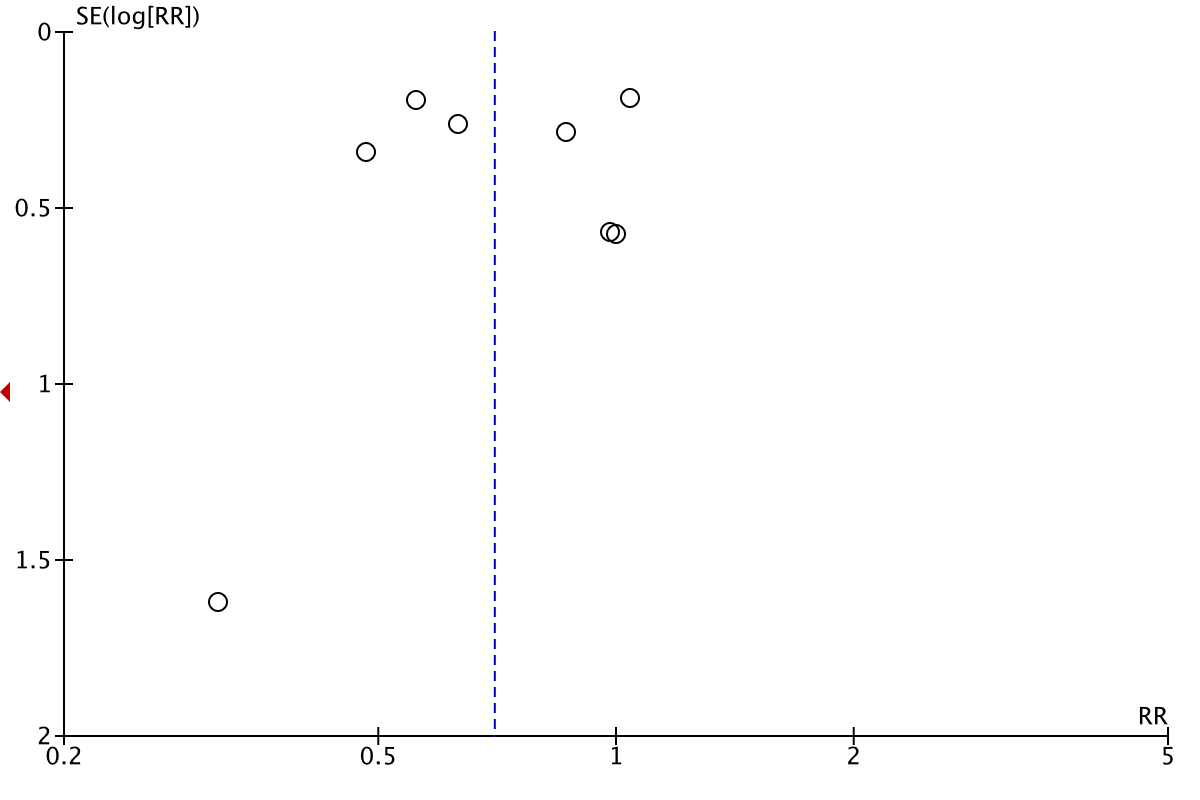


**Figure S3** Funnel plot evaluating publication bias for incidence of nausea between 0-2 hours (aprepitant). Dotted blue line, overall effect; Circles, studies; SE(Log[RR]), standard error (log [risk ratio]); RR, risk ratio.

**VOMITING**

a) Aprepitant


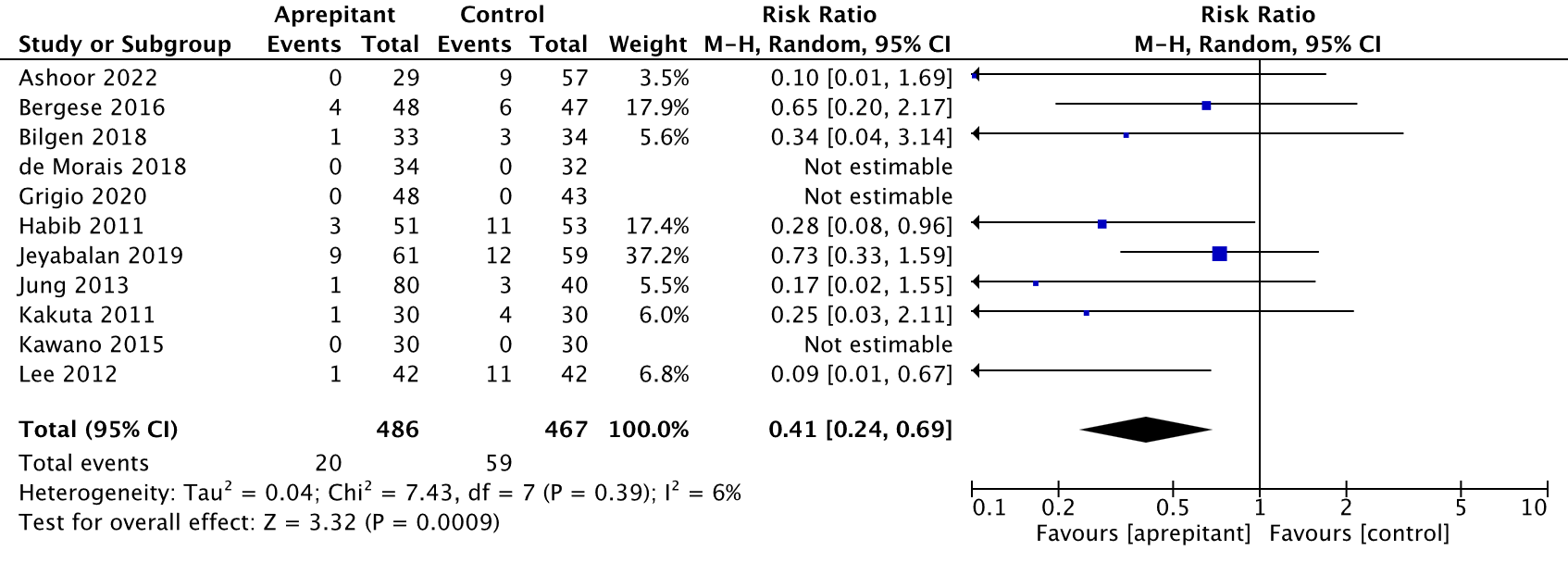


b) Fosaprepitant


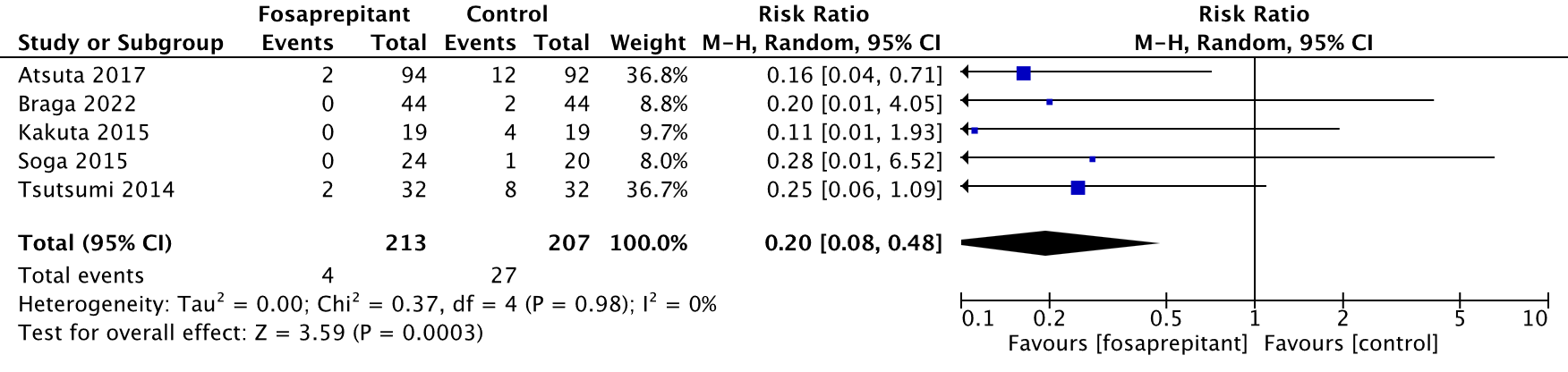


**Figure S4** Forest plot showing pooled risk ratio for the incidence of vomiting/retching between 0-2 hours after surgery; a) Comparison between aprepitant and control; b) Comparison between fosaprepitant to control. 95%CI, 95% confidence interval; df; degrees of freedom; I^2^, heterogeneity; M-H, random, Mantel-Haenszel random-effects model.


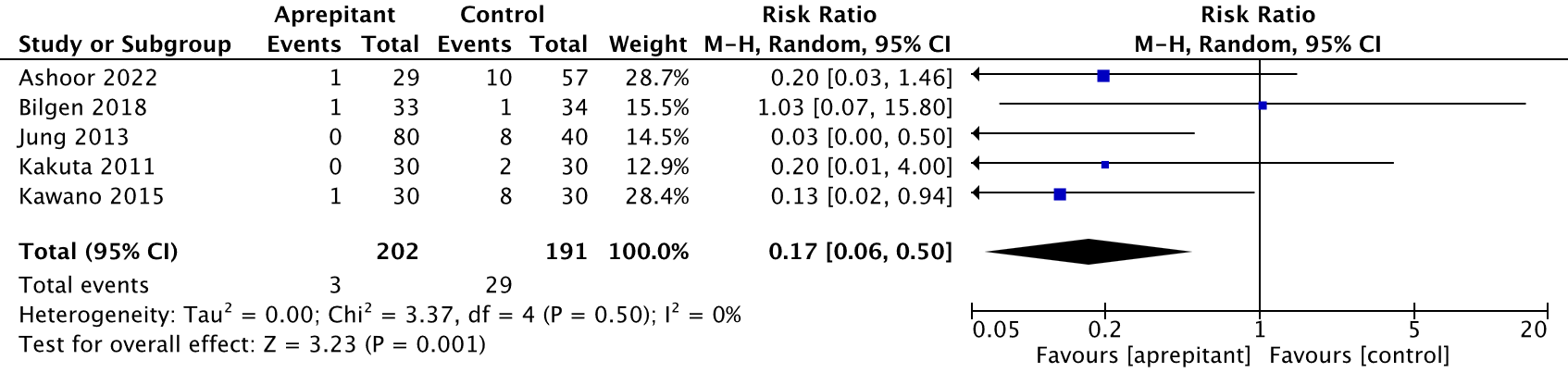


**Figure S5** Forest plot showing pooled risk ratio for the incidence of vomiting/retching between 2-24 hours after surgery. Comparison between aprepitant and control. 95%CI, 95% confidence interval; df; degrees of freedom; I^2^, heterogeneity; M-H, random, Mantel-Haenszel random-effects model.

**Box S2** Meta-regression for the incidence of vomiting between 0-24 hours after surgery. Comparison between aprepitant and control including the following moderators: type of surgery (cir), anesthesia type (anest), drug dose (dose), and monotherapy (mono).

| Mixed-Effects Model (k = 17; tau^2 estimator: REML)  logLik deviance AIC BIC AICc  -12.0248 24.0496 38.0496 40.8348 75.3829  tau^2 (estimated amount of residual heterogeneity): 0.1584 (SE = 0.1448)  tau (square root of estimated tau^2 value): 0.3980  I^2 (residual heterogeneity / unaccounted variability): 56.05%  H^2 (unaccounted variability / sampling variability): 2.28  R^2 (amount of heterogeneity accounted for): 0.00%  Test for Residual Heterogeneity:  QE(df = 11) = 23.5580, p-val = 0.0147  Test of Moderators (coefficients 2:6):  QM(df = 5) = 4.1552, p-val = 0.5273  Model Results:  estimate se zval pval ci.lb ci.ub  intrcpt 1.4891 1.5199 0.9797 0.3272 -1.4899 4.4680  cirHigh risk -0.2065 0.4452 -0.4639 0.6427 -1.0790 0.6660  cirMixed/Unknown 0.7678 0.7491 1.0251 0.3053 -0.7003 2.2360  anest -1.0832 0.9334 -1.1605 0.2459 -2.9127 0.7463  dose -0.0300 0.0176 -1.7069 0.0878 -0.0644 0.0044 .  mono 0.3513 0.4483 0.7837 0.4332 -0.5273 1.2300  ---  Signif. codes: 0 ‘***’ 0.001 ‘**’ 0.01 ‘*’ 0.05 ‘.’ 0.1 ‘ ’ 1 |
| --- |


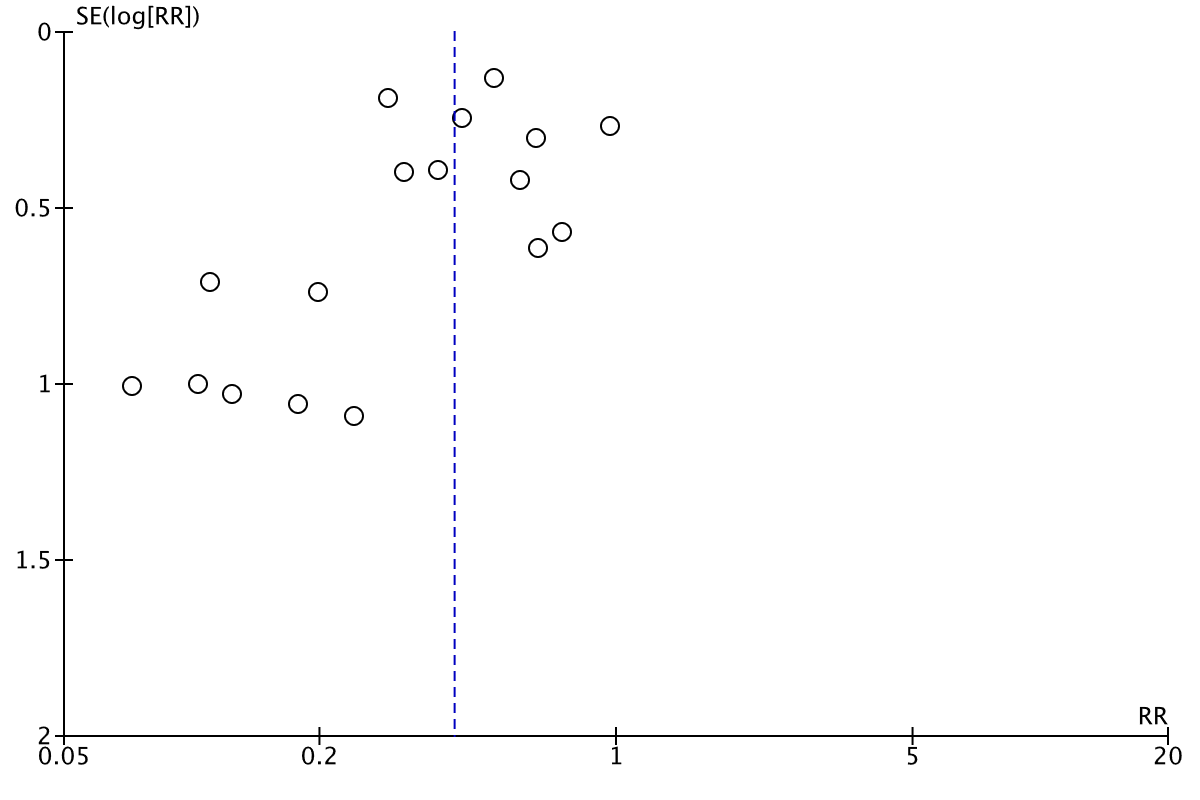


**Figure S6** Funnel plot evaluating publication bias for incidence of vomiting between 0-24h (aprepitant). Dotted blue line, overall effect; Circles, studies; SE(Log[RR]), standard error (log [risk ratio]); RR, risk ratio.

**
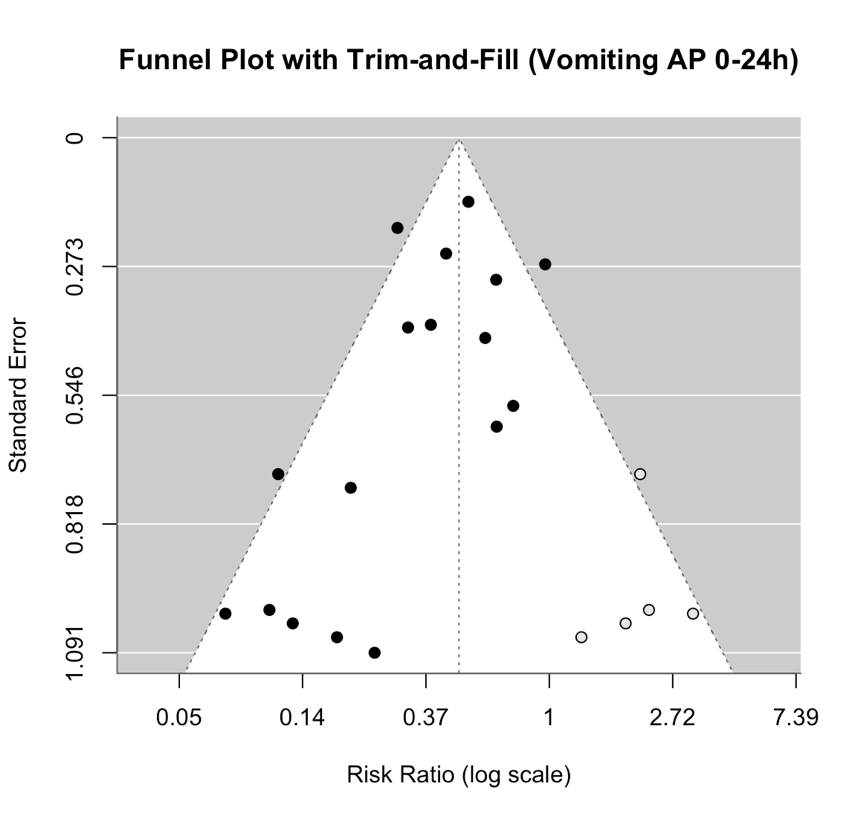
**

**Figure S6.1** Funnel plot with trim-and-fill analysis evaluating publication bias for incidence of vomiting between 0-24h (aprepitant). Black circles: includes studies. Gray circles: estimated missing studies on the right side.


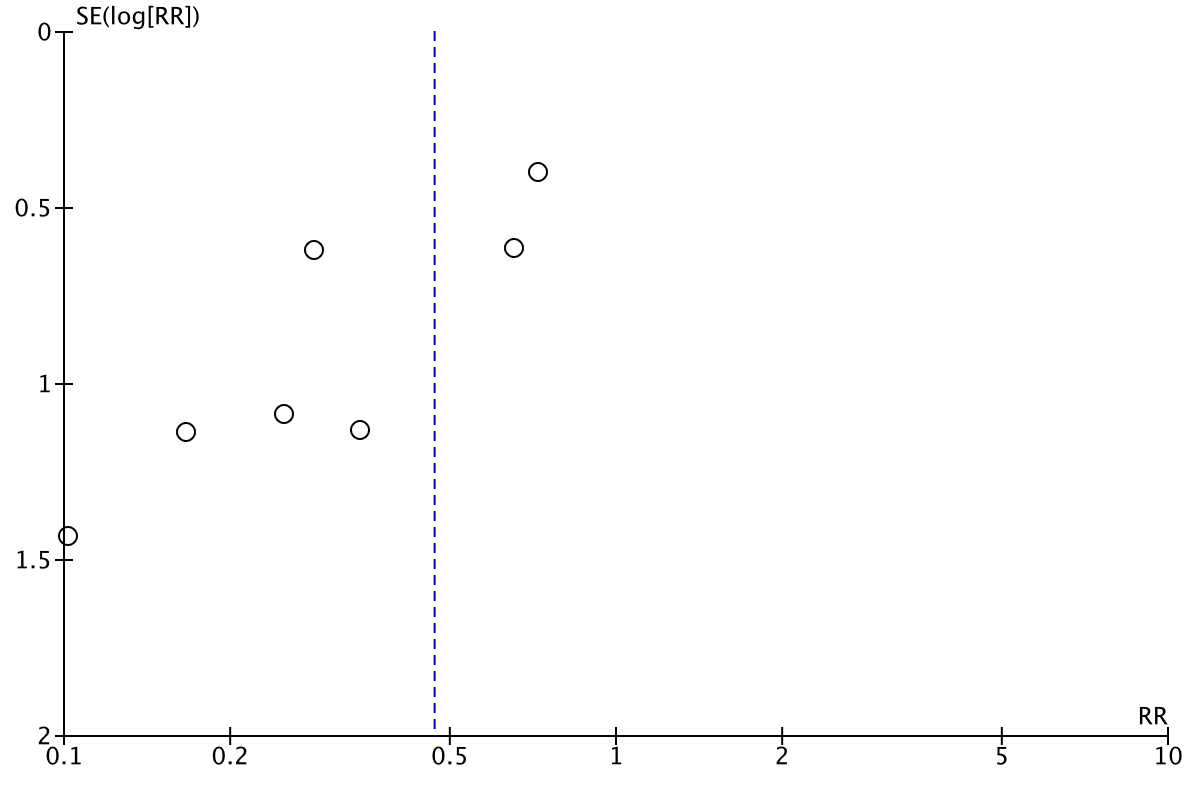


**Figure S7** Funnel plot evaluating publication bias for incidence of vomiting between 0-2h (aprepitant). Dotted blue line, overall effect; Circles, studies; SE(Log[RR]), standard error (log [risk ratio]); RR, risk ratio.

**RESCUE ANTIEMETIC USE**


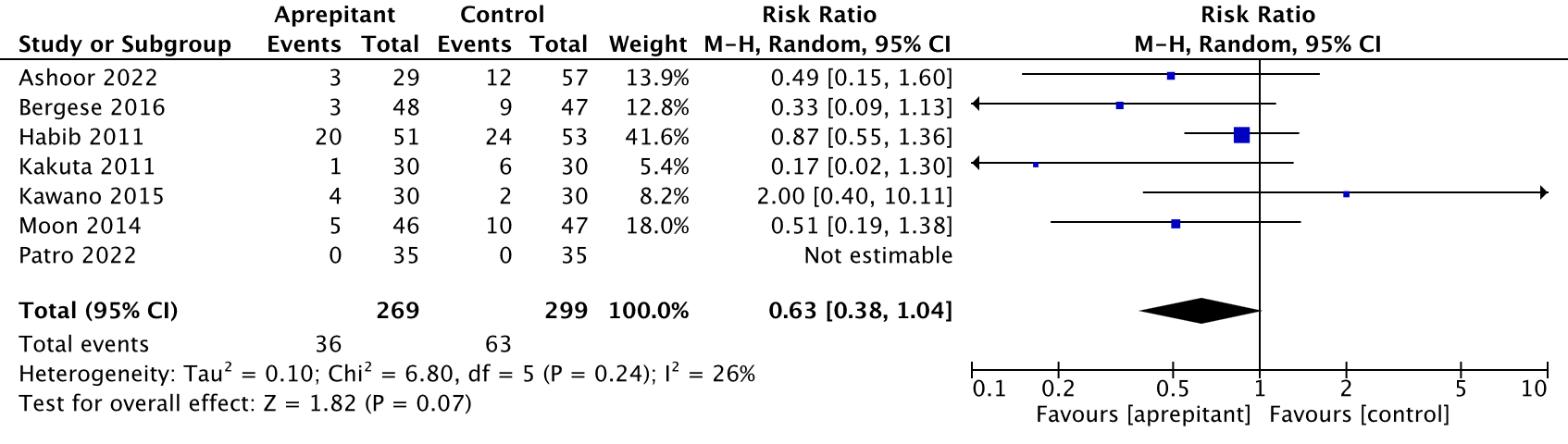


**Figure S8** Forest plot showing pooled risk ratio for the incidence of rescue antiemetic use between 0-2 hours after surgery. Comparison between aprepitant and control; 95%CI, 95% confidence interval, df; degrees of freedom; I^2^, heterogeneity; M-H, random, Mantel-Haenszel random-effects model.

**Box S3** Meta-regression for incidence of rescue antiemetic use between 0-24 hours after surgery. Comparison between aprepitant and control including the following moderators: type of surgery (cir), anesthesia type (anest), drug dose (dose), and monotherapy (mono).

| Mixed-Effects Model (k = 14; tau^2 estimator: REML)  logLik deviance AIC BIC AICc  -3.1612 6.3223 20.3223 20.8784 132.3223  tau^2 (estimated amount of residual heterogeneity): 0.0037 (SE = 0.0145)  tau (square root of estimated tau^2 value): 0.0605  I^2 (residual heterogeneity / unaccounted variability): 8.93%  H^2 (unaccounted variability / sampling variability): 1.10  R^2 (amount of heterogeneity accounted for): 93.24%  Test for Residual Heterogeneity:  QE(df = 8) = 11.2297, p-val = 0.1890  Test of Moderators (coefficients 2:6):  QM(df = 5) = 14.3198, p-val = 0.0137  Model Results:  estimate se zval pval ci.lb ci.ub  intrcpt 0.5908 0.8821 0.6698 0.5030 -1.1381 2.3197  cirHigh risk -0.1919 0.2099 -0.9143 0.3606 -0.6032 0.2194  cirMixed/Unknown 0.7170 0.3834 1.8702 0.0615 -0.0344 1.4684 .  anest 0.0122 0.6459 0.0189 0.9849 -1.2537 1.2781  dose -0.0190 0.0087 -2.1953 0.0281 -0.0361 -0.0020 *  mono 0.1987 0.1859 1.0687 0.2852 -0.1657 0.5631  ---  Signif. codes: 0 ‘***’ 0.001 ‘**’ 0.01 ‘*’ 0.05 ‘.’ 0.1 ‘ ’ 1 |
| --- |


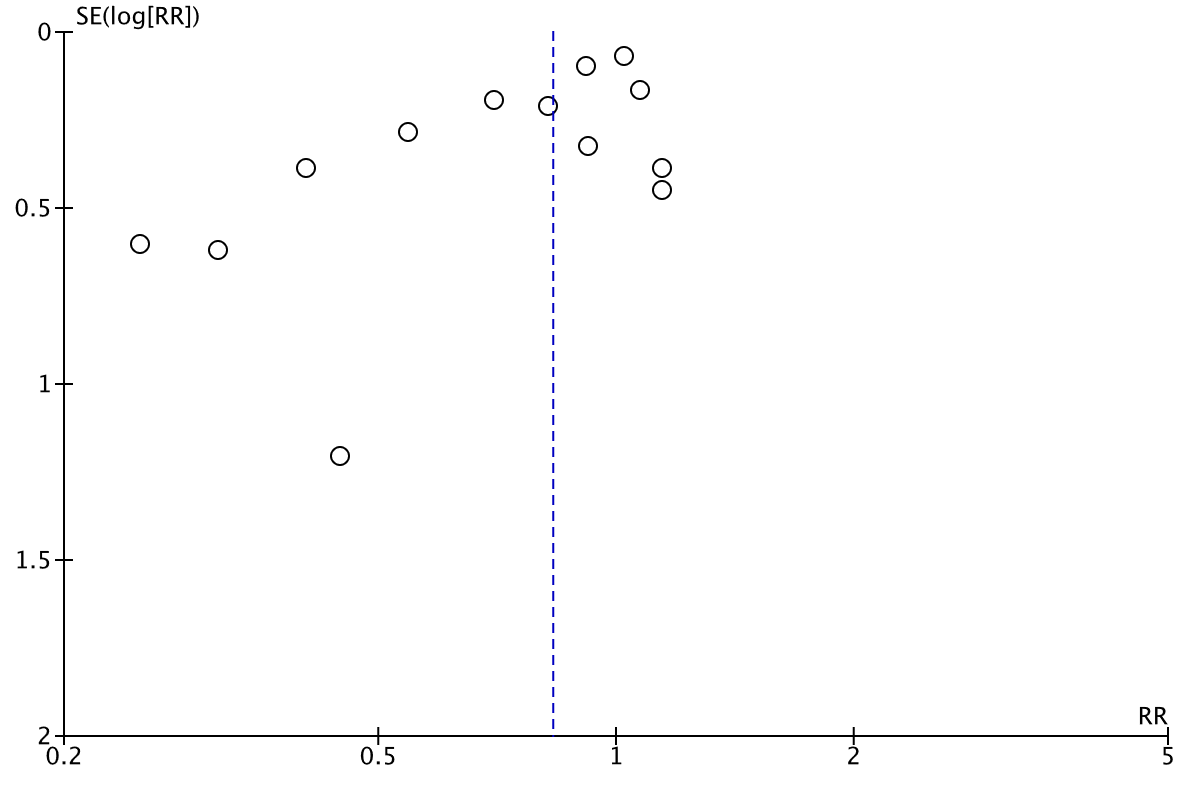


**Figure S9** Funnel plot evaluating publication bias for rescue antiemetic use 0-24h (aprepitant). Dotted blue line, overall effect; Circles, studies; SE(Log[RR]), standard error (log [risk ratio]); RR, risk ratio.

**
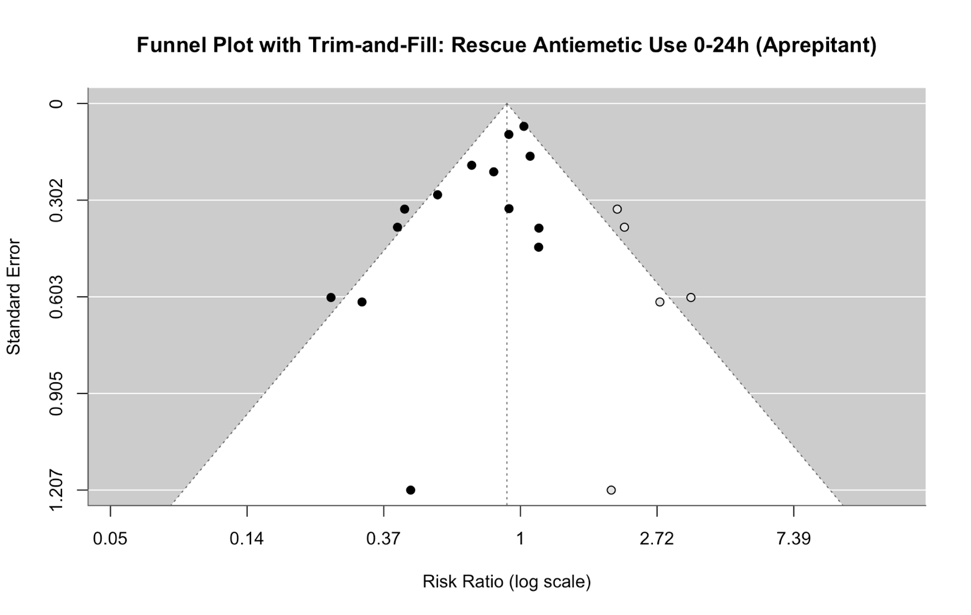
**

**Figure S9.1** Funnel plot with trim-and-fill analysis evaluating publication bias for rescue antiemetic use 0-24h (aprepitant). Black circles: includes studies. Gray circles: estimated missing studies on the right side.

**SUBGROUPS ANALYSIS**

- Type of anaesthesia (inhalational anaesthesia)

**
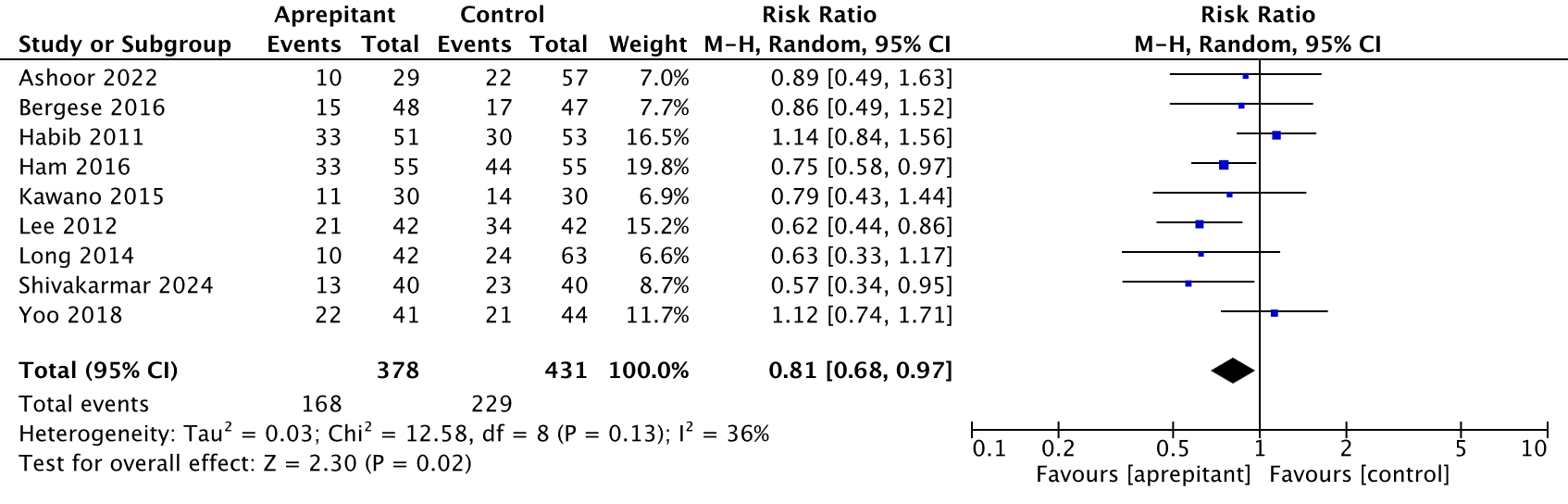
**

**Figure S10 Subgroup meta-analysis** Forest plot showing pooled risk ratio for the incidence of nausea between 0-24 hours after surgery. Comparison between aprepitant and control in patients who received inhalational anaesthesia; 95%CI, 95% confidence interval, df; degrees of freedom; I^2^, heterogeneity; M-H, random, Mantel-Haenszel random-effects model.


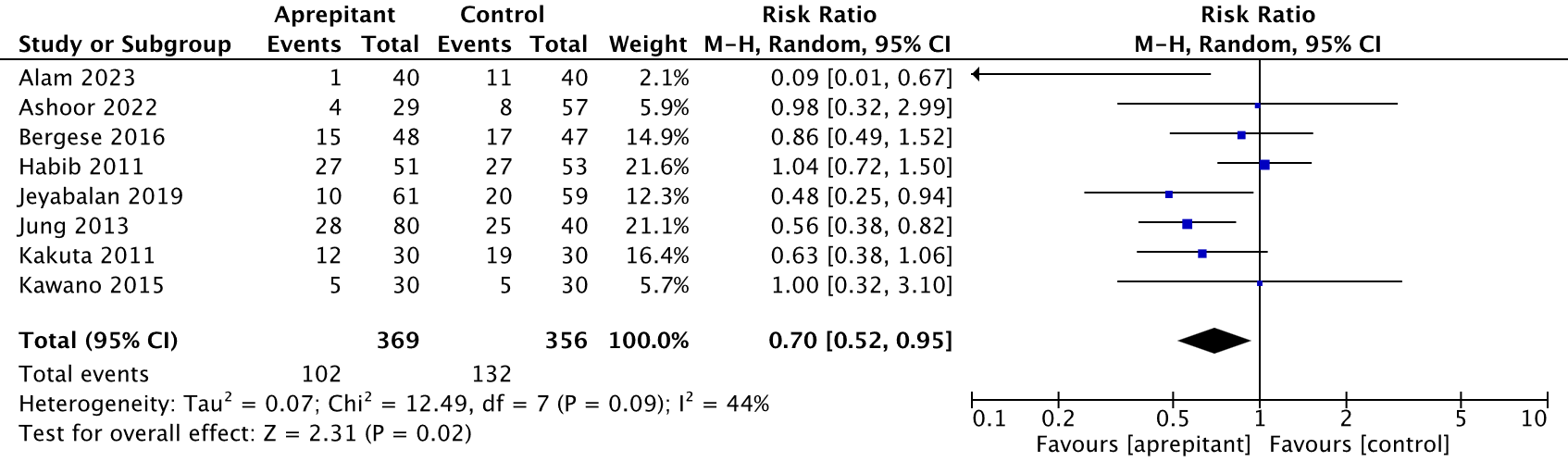


**Figure S11 Subgroup meta-analysis** Forest plot showing pooled risk ratio for the incidence of nausea between 0-2 hours after surgery. Comparison between aprepitant and control in patients who received inhalational anaesthesia; 95%CI, 95% confidence interval, df; degrees of freedom; I^2^, heterogeneity; M-H, random, Mantel-Haenszel random-effects model.


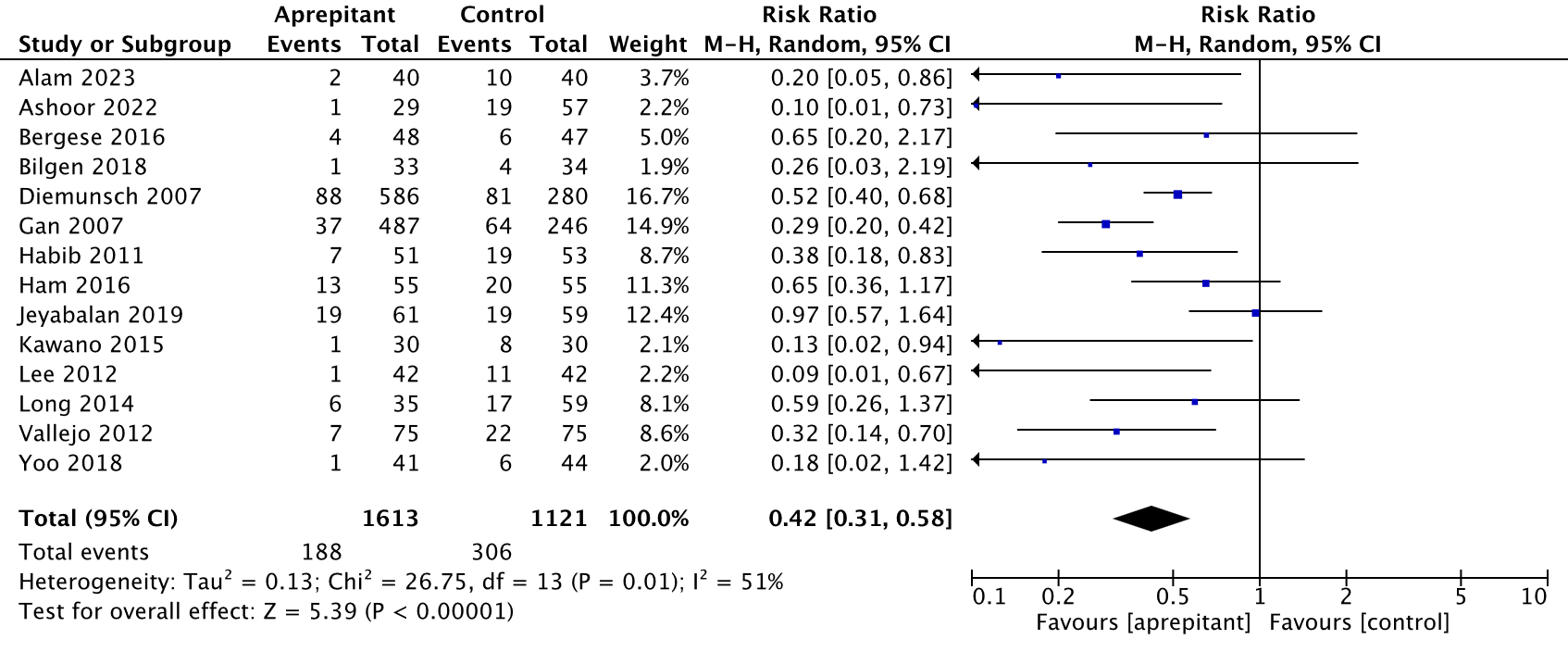


**Figure S12 Subgroup meta-analysis** Forest plot showing pooled risk ratio for the incidence of vomiting/retching between 0-24 hours after surgery. Comparison between aprepitant and control in patients who received inhalational anesthesia; 95%CI, 95% confidence interval, df; degrees of freedom; I^2^, heterogeneity; M-H, random, Mantel-Haenszel random-effects model.


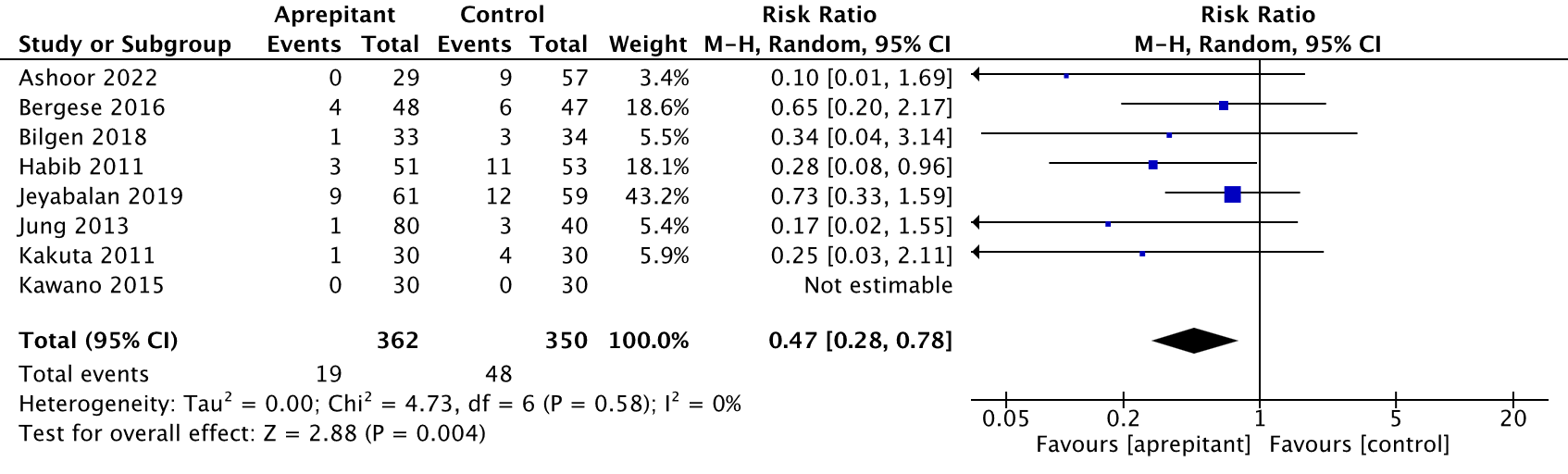


**Figure S13 Subgroup meta-analysis** Forest plot showing pooled risk ratio for the incidence of vomiting/retching between 0-2 hours after surgery. Comparison between aprepitant and control in patients who received inhalational anesthesia; 95%CI, 95% confidence interval, df; degrees of freedom; I^2^, heterogeneity; M-H, random, Mantel-Haenszel random-effects model.


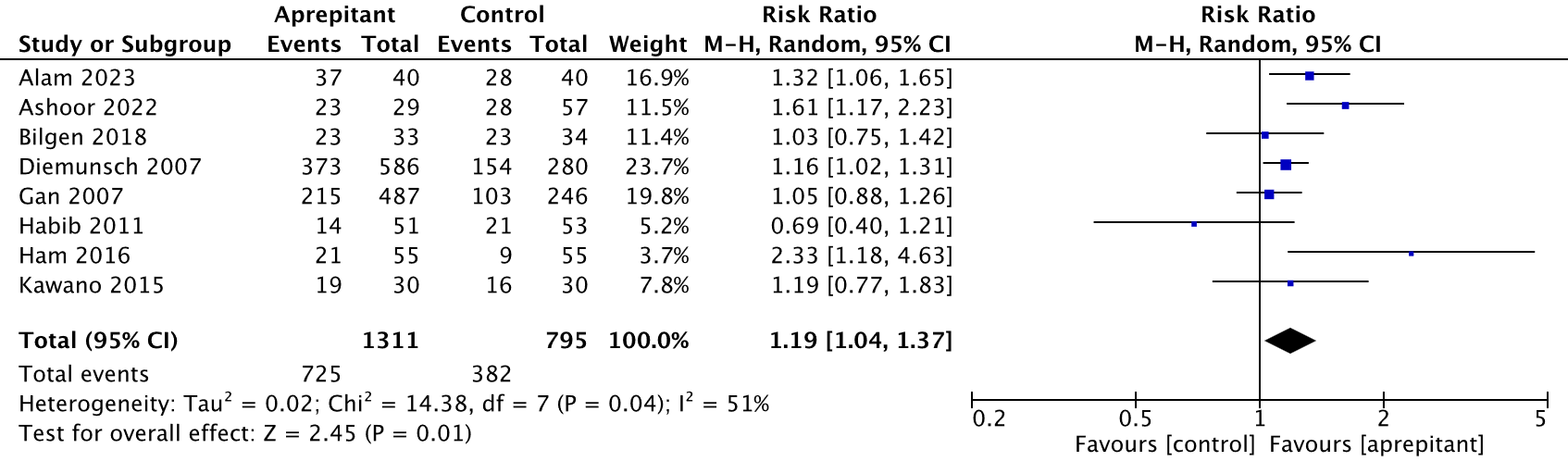


**Figure S14 Subgroup meta-analysis** Forest plot showing pooled risk ratio for the incidence of complete response between 0-24 hours after surgery. Comparison between aprepitant and control in patients who received inhalational anesthesia; 95%CI, 95% confidence interval, df; degrees of freedom; I^2^, heterogeneity; M-H, random, Mantel-Haenszel random-effects model.


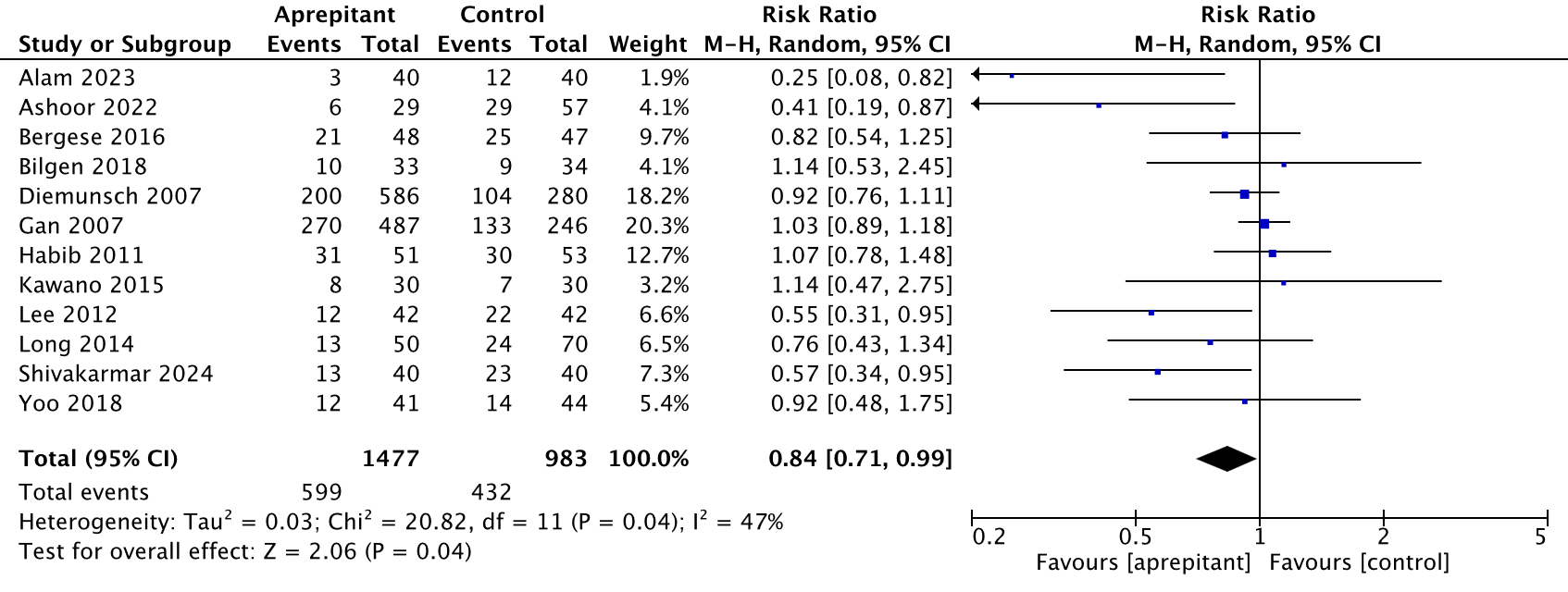


**Figure S15 Subgroup meta-analysis** Forest plot showing pooled risk ratio for the incidence of rescue antiemetic use between 0-24 hours after surgery. Comparison between aprepitant and control in patients who received inhalational anesthesia; 95%CI, 95% confidence interval, df; degrees of freedom; I^2^, heterogeneity; M-H, random, Mantel-Haenszel random-effects model.

- Mono or multitherapy with antiemetics


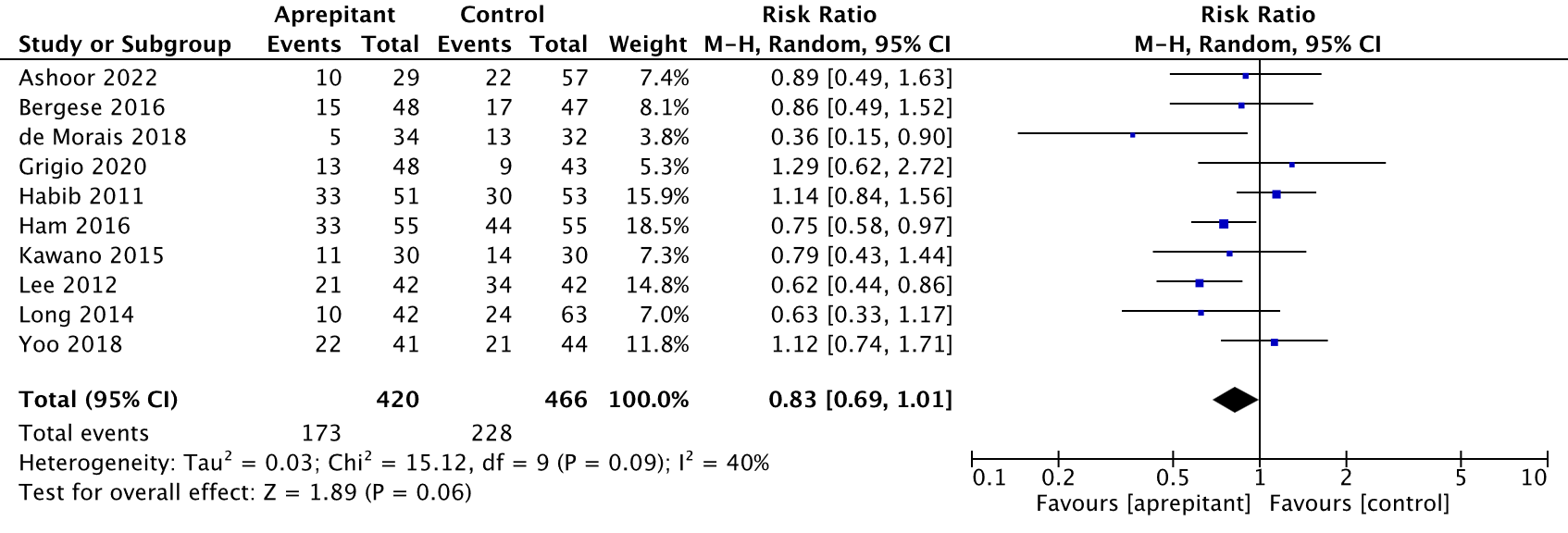


**Figure S16 Subgroup meta-analysis** Forest plot showing pooled risk ratio for the incidence of nausea between 0-24 hours after surgery. Comparison between aprepitant and control in patients who received multiple antiemetics; 95%CI, 95% confidence interval, df; degrees of freedom; I^2^, heterogeneity; M-H, random, Mantel-Haenszel random-effects model.


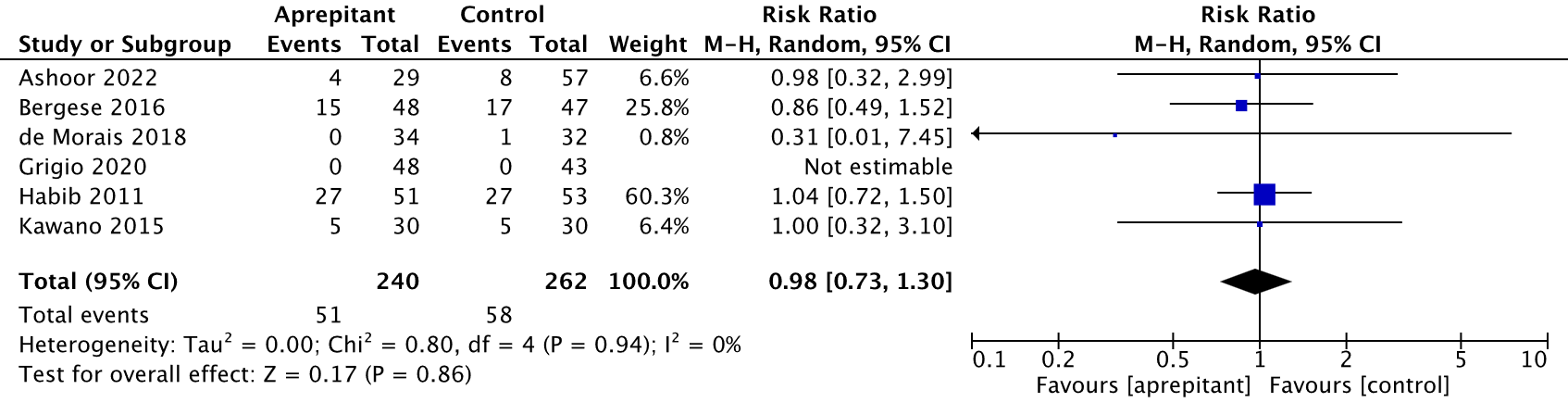


**Figure S17 Subgroup meta-analysis** Forest plot showing pooled risk ratio for the incidence of nausea between 0-2 hours after surgery. Comparison between aprepitant and control in patients who received multiple antiemetics; 95%CI, 95% confidence interval, df; degrees of freedom; I^2^, heterogeneity; M-H, random, Mantel-Haenszel random-effects model.


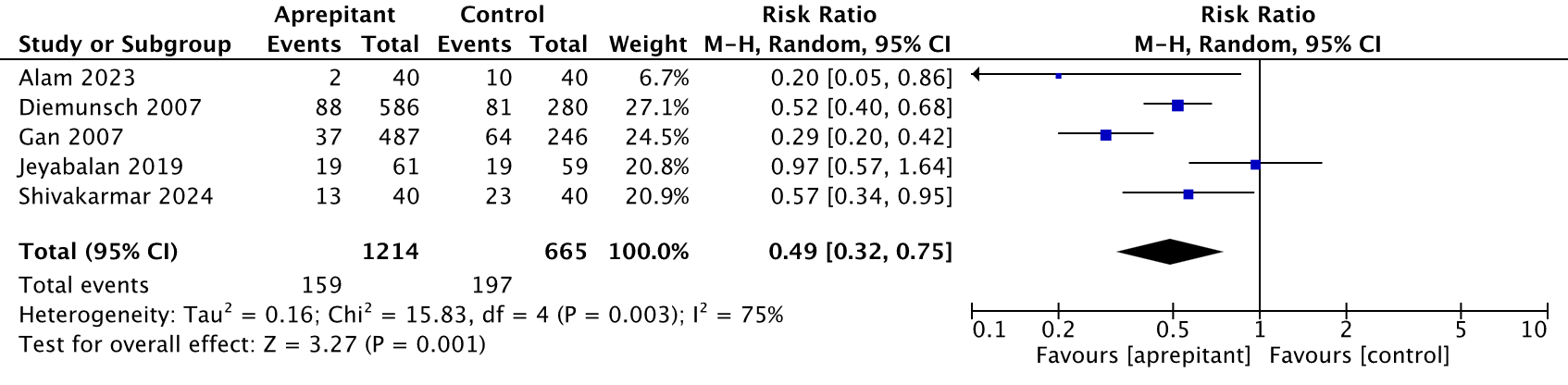


**Figure S18 Subgroup meta-analysis** Forest plot showing pooled risk ratio for the incidence of vomiting/retching between 0-24 hours after surgery. Comparison between aprepitant and control in patients who received only one antiemetic; 95%CI, 95% confidence interval, df; degrees of freedom; I^2^, heterogeneity; M-H, random, Mantel-Haenszel random-effects model.


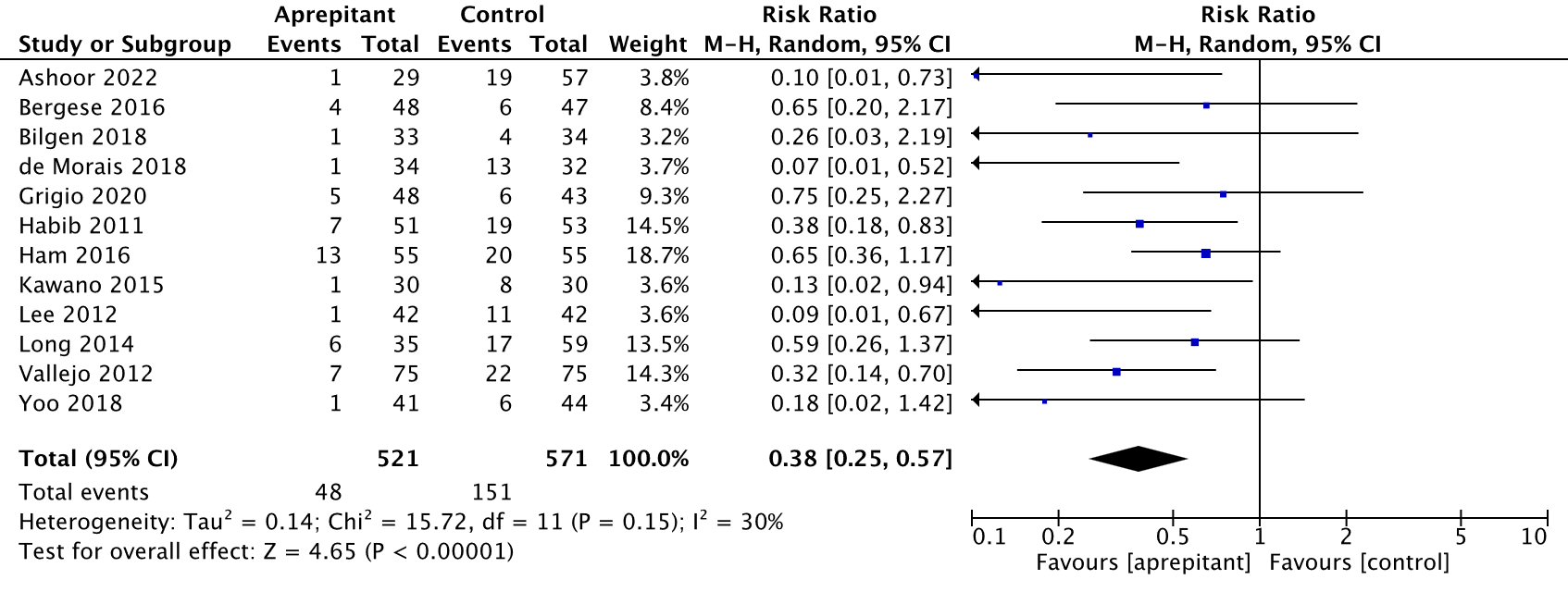


**Figure S19 Subgroup meta-analysis** Forest plot showing pooled risk ratio for the incidence of vomiting/retching between 0-24 hours after surgery. Comparison between aprepitant and control in patients who received multiple antiemetics; 95%CI, 95% confidence interval, df; degrees of freedom; I^2^, heterogeneity; M-H, random, Mantel-Haenszel random-effects model.


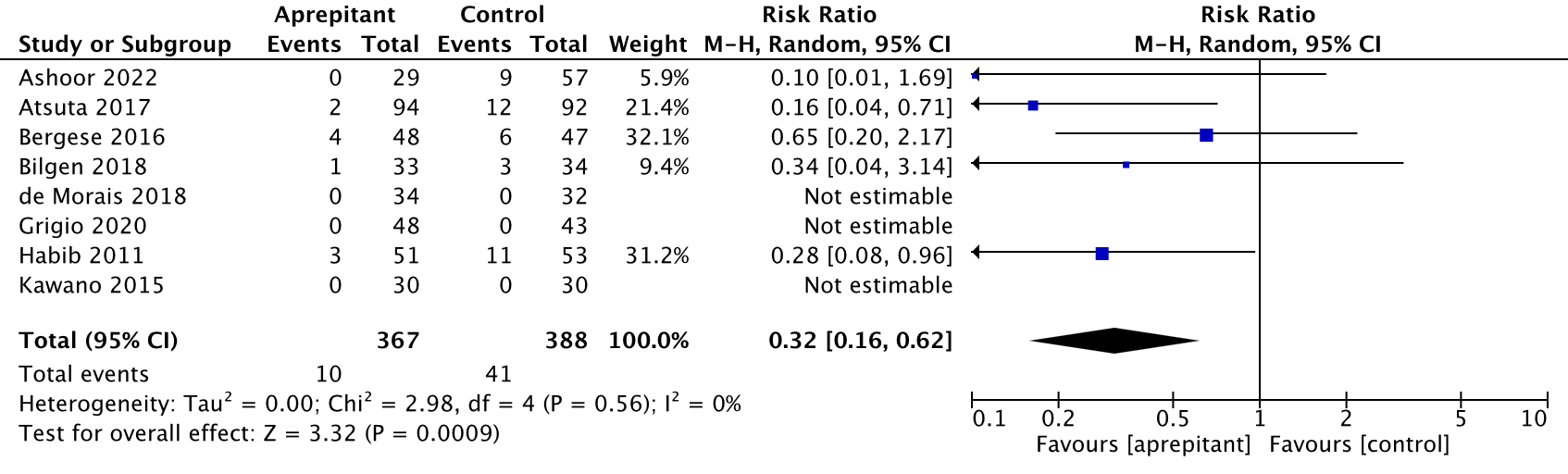


**Figure S20 Subgroup meta-analysis** Forest plot showing pooled risk ratio for the incidence of vomiting/retching between 0-2 hours after surgery. Comparison between aprepitant and control in patients who received multiple antiemetics; 95%CI, 95% confidence interval, df; degrees of freedom; I^2^, heterogeneity; M-H, random, Mantel-Haenszel random-effects model.


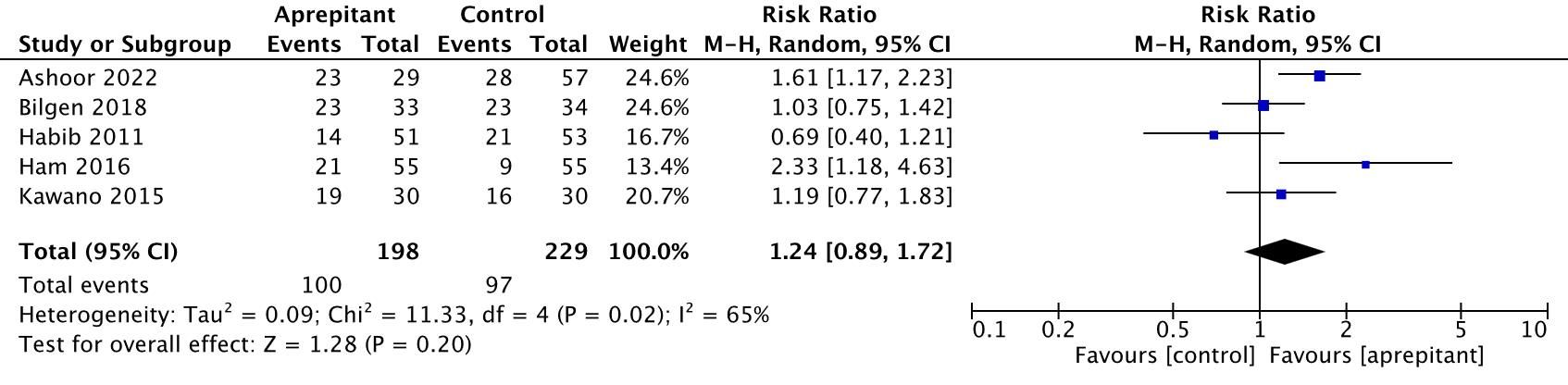


**Figure S21 Subgroup meta-analysis** Forest plot showing pooled risk ratio for the incidence of complete response rate between 0-24 hours after surgery. Comparison between aprepitant and control in patients who received multiple antiemetics; 95%CI, 95% confidence interval, df; degrees of freedom; I^2^, heterogeneity; M-H, random, Mantel-Haenszel random-effects model.

**
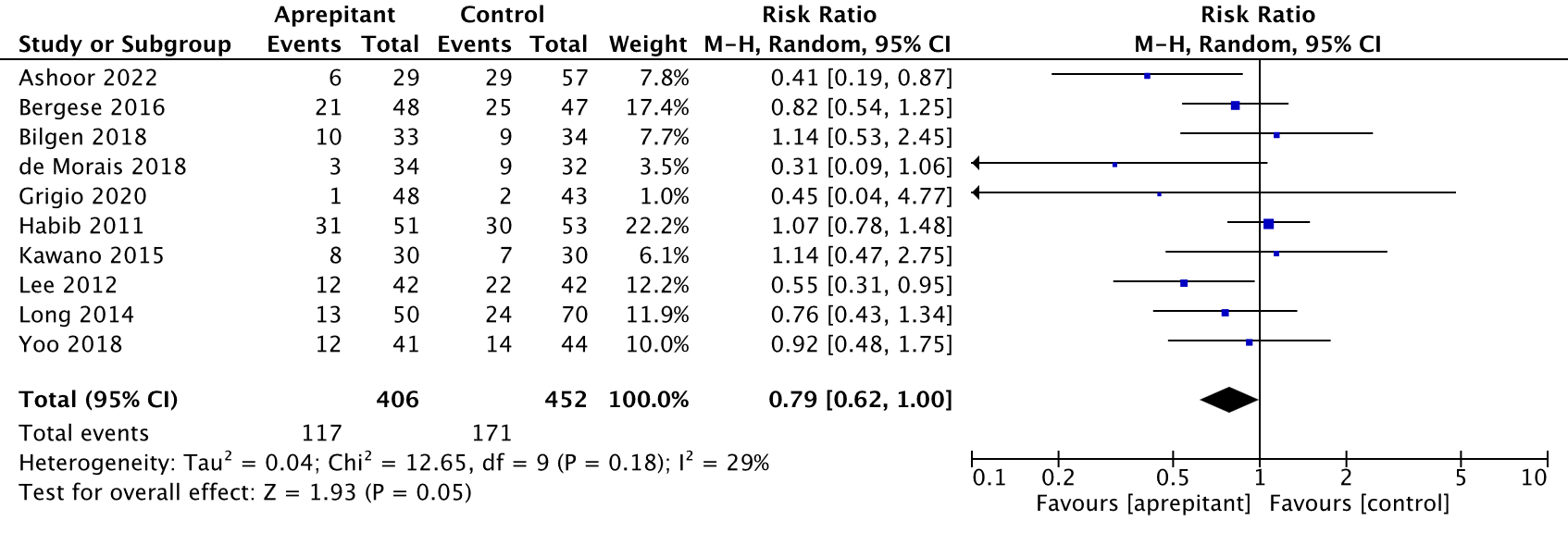
 Figure S22 Subgroup meta-analysis** Forest plot showing pooled risk ratio for the incidence of rescue antiemetic use between 0-24 hours after surgery. Comparison between aprepitant and control in patients who received multiple antiemetics; 95%CI, 95% confidence interval, df; degrees of freedom; I^2^, heterogeneity; M-H, random, Mantel-Haenszel random-effects model.

- Different doses of aprepitant


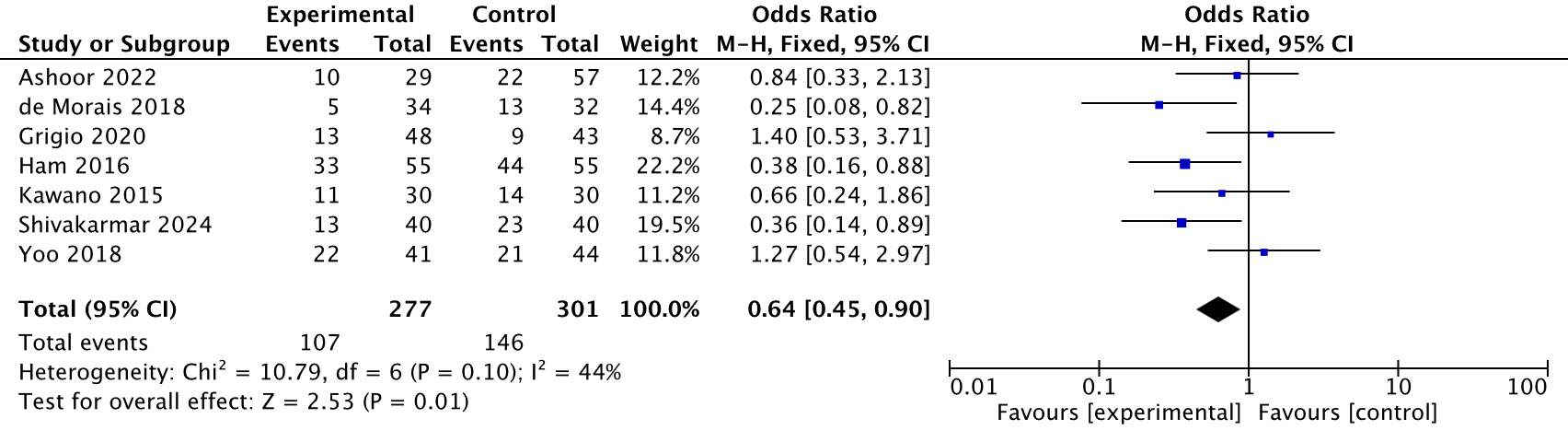


**Figure S23 Subgroup meta-analysis** Forest plot showing pooled risk ratio for the incidence of nausea between 0-24 hours after surgery. Comparison between aprepitant 80mg and control; 95%CI, 95% confidence interval, df; degrees of freedom; I^2^, heterogeneity; M-H, random, Mantel-Haenszel random-effects model.


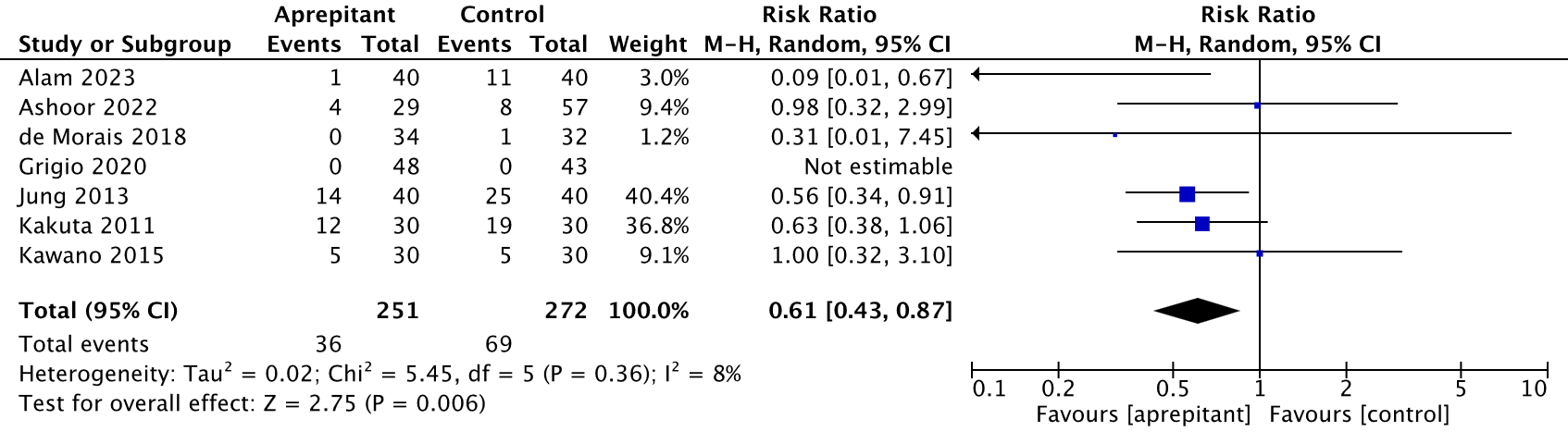


**Figure S24 Subgroup meta-analysis** Forest plot showing pooled risk ratio for the incidence of nausea between 0-2 hours after surgery. Comparison between aprepitant 80mg and control; 95%CI, 95% confidence interval, df; degrees of freedom; I^2^, heterogeneity; M-H, random, Mantel-Haenszel random-effects model.


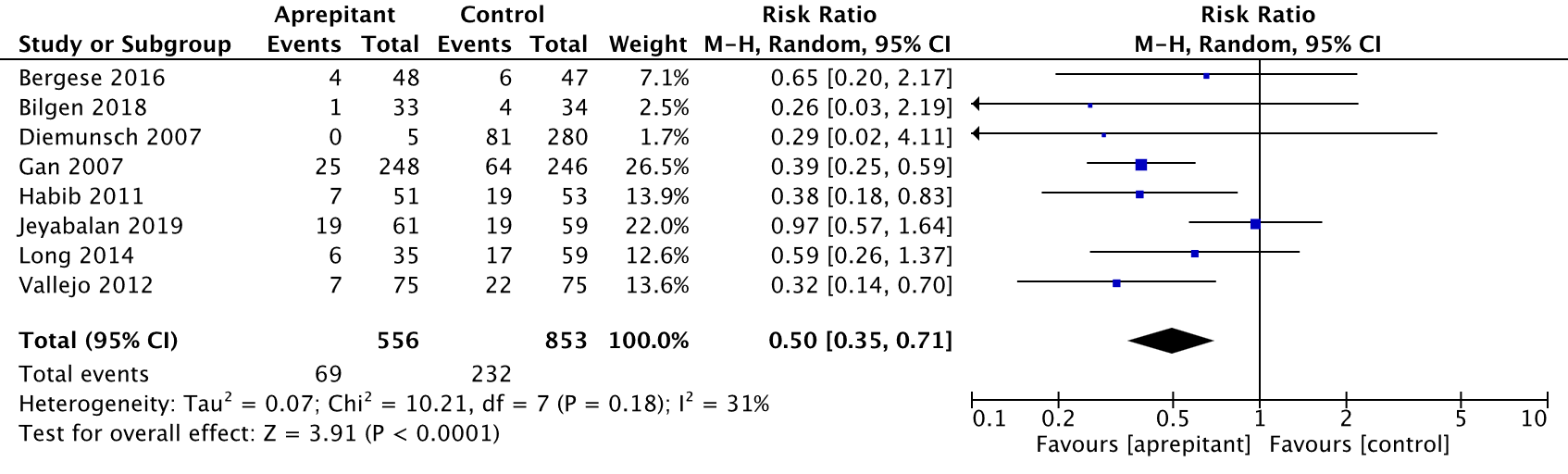


**Figure S25 Subgroup meta-analysis** Forest plot showing pooled risk ratio for the incidence of vomiting/retching between 0-24 hours after surgery. Comparison between aprepitant 40mg and control; 95%CI, 95% confidence interval, df; degrees of freedom; I^2^, heterogeneity; M-H, random, Mantel-Haenszel random-effects model.


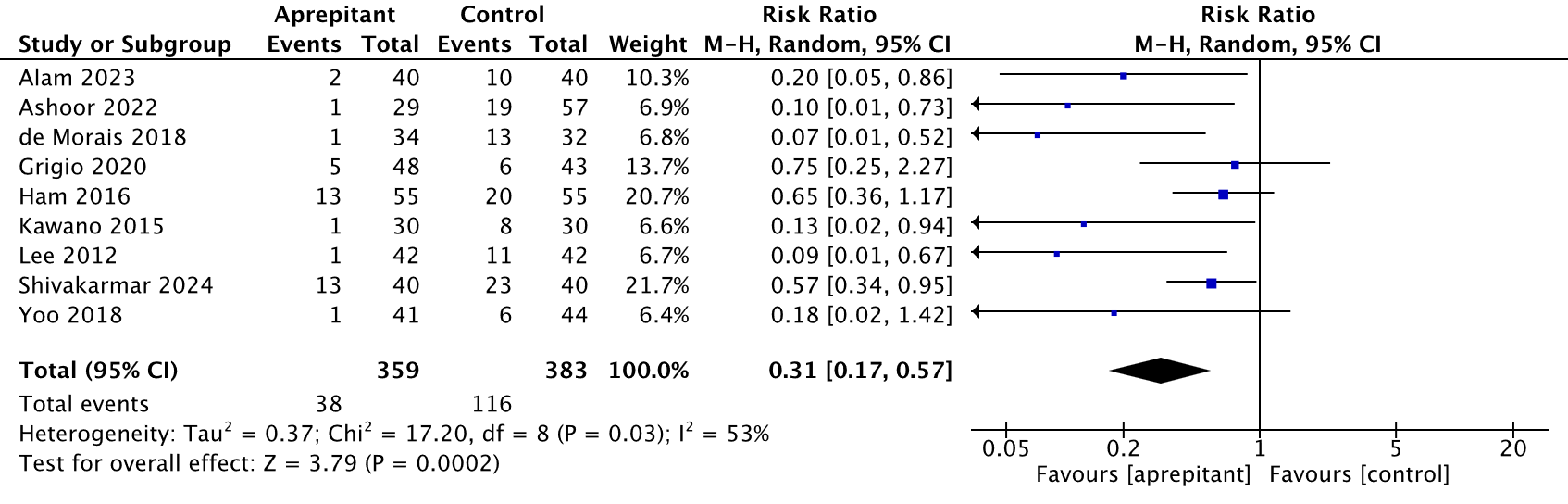


**Figure S26 Subgroup meta-analysis** Forest plot showing pooled risk ratio for the incidence of vomiting/retching between 0-24 hours after surgery. Comparison between aprepitant 80mg and control; 95%CI, 95% confidence interval, df; degrees of freedom; I^2^, heterogeneity; M-H, random, Mantel-Haenszel random-effects model.


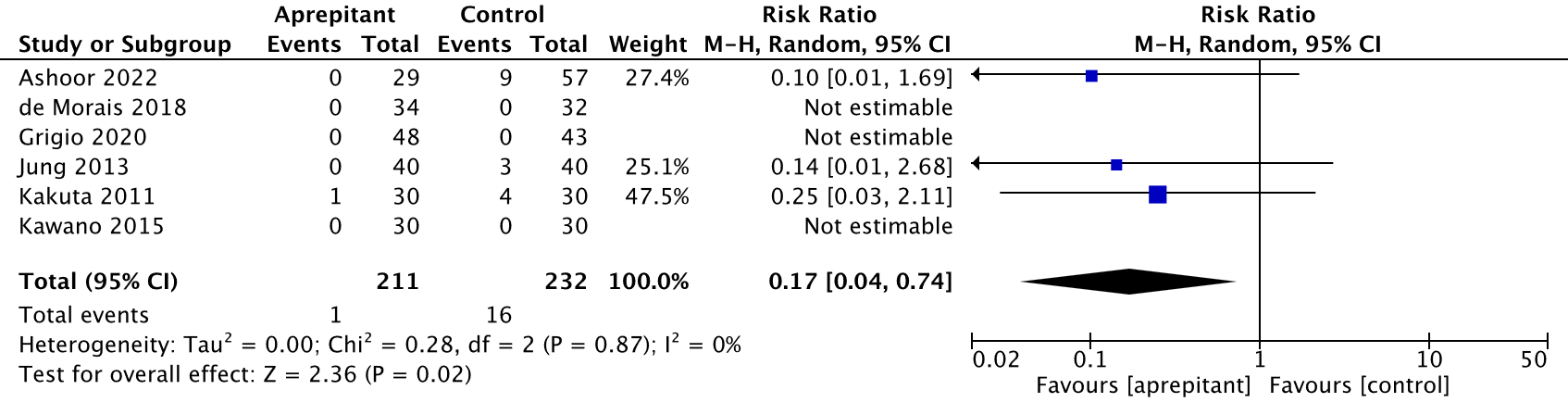


**Figure S27 Subgroup meta-analysis** Forest plot showing pooled risk ratio for the incidence of vomiting/retching between 0-2 hours after surgery. Comparison between aprepitant 80mg and control; 95%CI, 95% confidence interval, df; degrees of freedom; I^2^, heterogeneity; M-H, random, Mantel-Haenszel random-effects model.


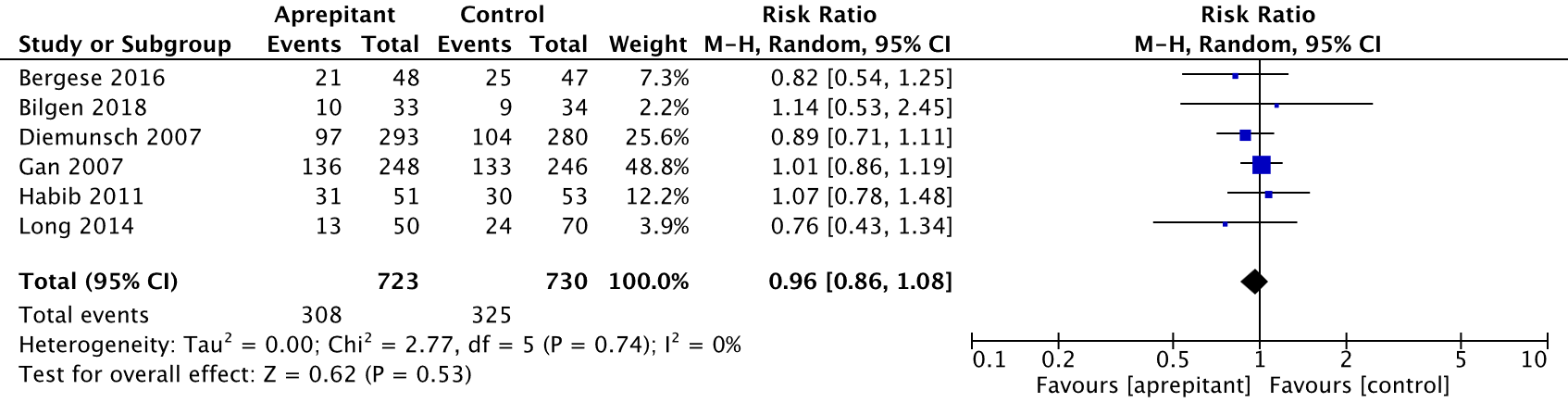


**Figure S28 Subgroup meta-analysis** Forest plot showing pooled risk ratio for the incidence of complete response rate between 0-24 hours after surgery. Comparison between aprepitant 40mg and control; 95%CI, 95% confidence interval, df; degrees of freedom; I^2^, heterogeneity; M-H, random, Mantel-Haenszel random-effects model.


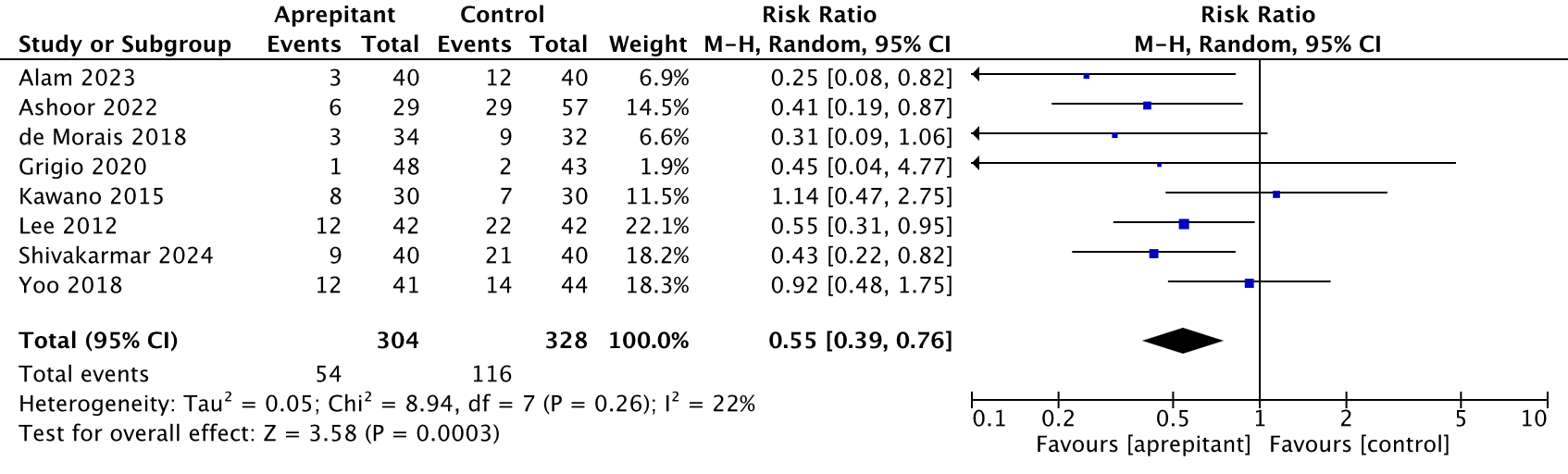


**Figure S29 Subgroup meta-analysis** Forest plot showing pooled risk ratio for the incidence of complete response rate between 0-24 hours after surgery. Comparison between aprepitant 80mg and control; 95%CI, 95% confidence interval, df; degrees of freedom; I^2^, heterogeneity; M-H, random, Mantel-Haenszel random-effects model.

- Pure effect


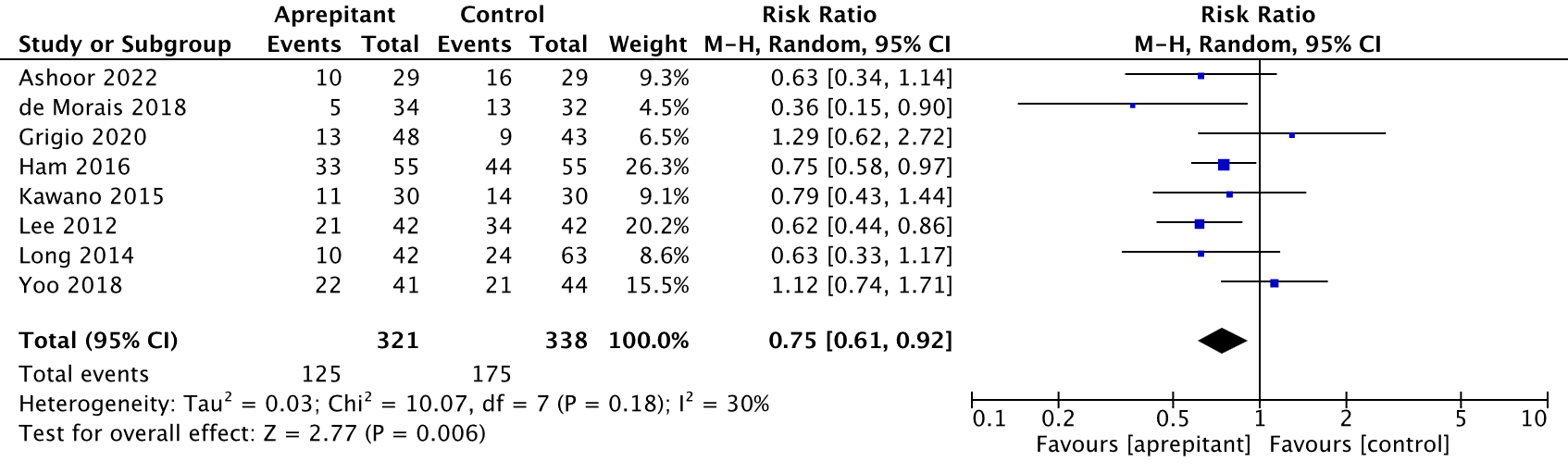


**Figure S30 Subgroup meta-analysis** Forest plot showing pooled risk ratio for the incidence of nausea between 0-24 hours after surgery. Comparison between “pure effect” and control; 95%CI, 95% confidence interval, df; degrees of freedom; I^2^, heterogeneity; M-H, random, Mantel-Haenszel random-effects model.


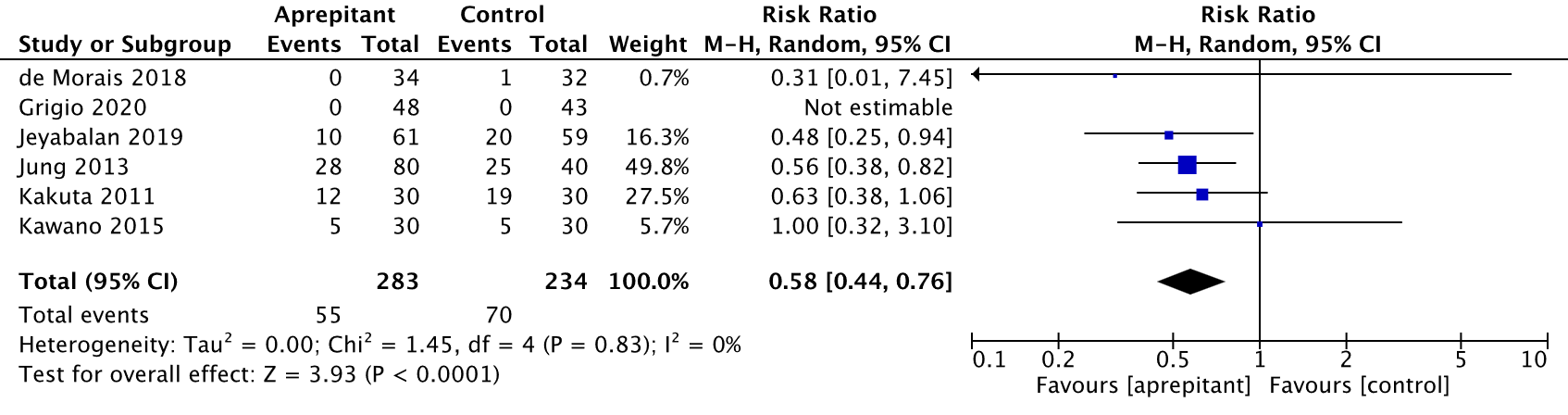


**Figure S31 Subgroup meta-analysis** Forest plot showing pooled risk ratio for the incidence of nausea between 0-2 hours after surgery. Comparison between “pure effect” and control; 95%CI, 95% confidence interval, df; degrees of freedom; I^2^, heterogeneity; M-H, random, Mantel-Haenszel random-effects model.


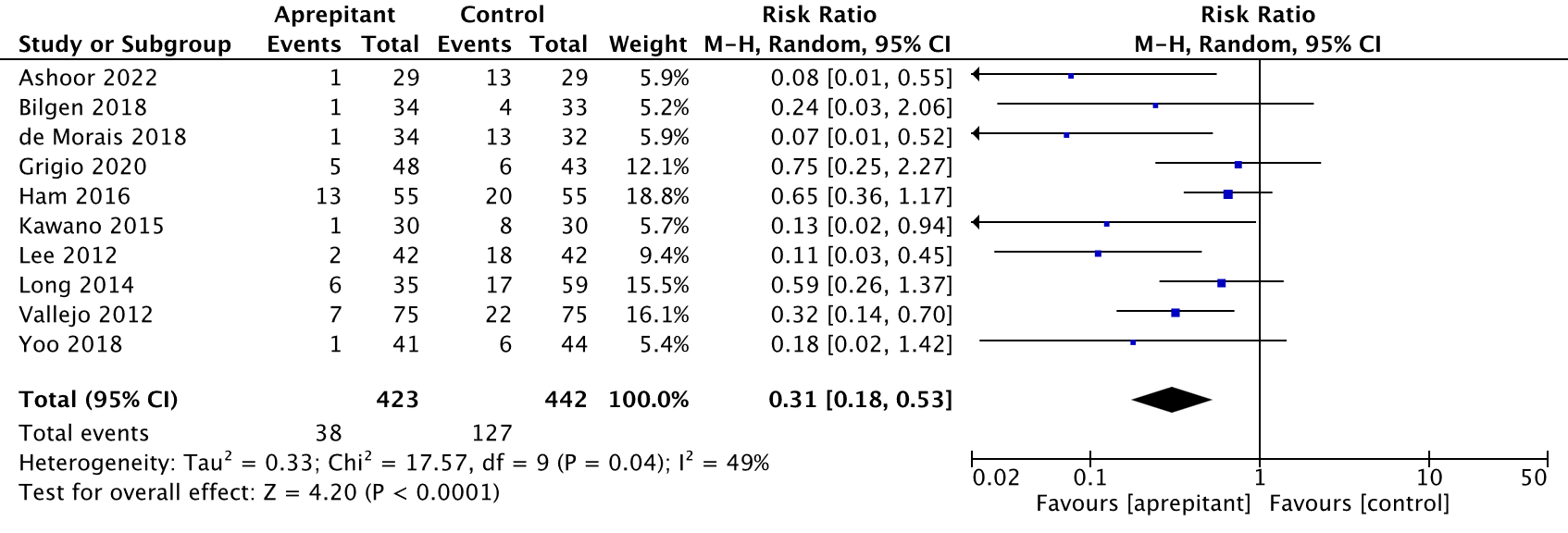


**Figure S32 Subgroup meta-analysis** Forest plot showing pooled risk ratio for the incidence of vomiting/retching between 0-24 hours after surgery. Comparison between “pure effect” and control; 95%CI, 95% confidence interval, df; degrees of freedom; I^2^, heterogeneity; M-H, random, Mantel-Haenszel random-effects model.


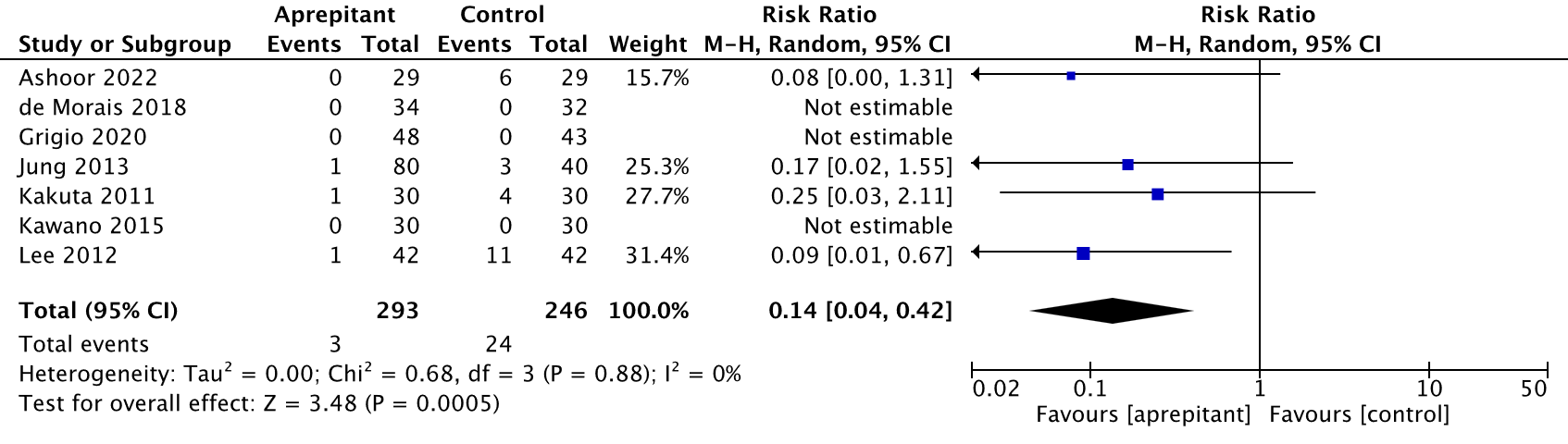


**Figure S33 Subgroup meta-analysis** Forest plot showing pooled risk ratio for the incidence of vomiting/retching between 0-2 hours after surgery. Comparison between “pure effect” and control; 95%CI, 95% confidence interval, df; degrees of freedom; I^2^, heterogeneity; M-H, random, Mantel-Haenszel random-effects model.


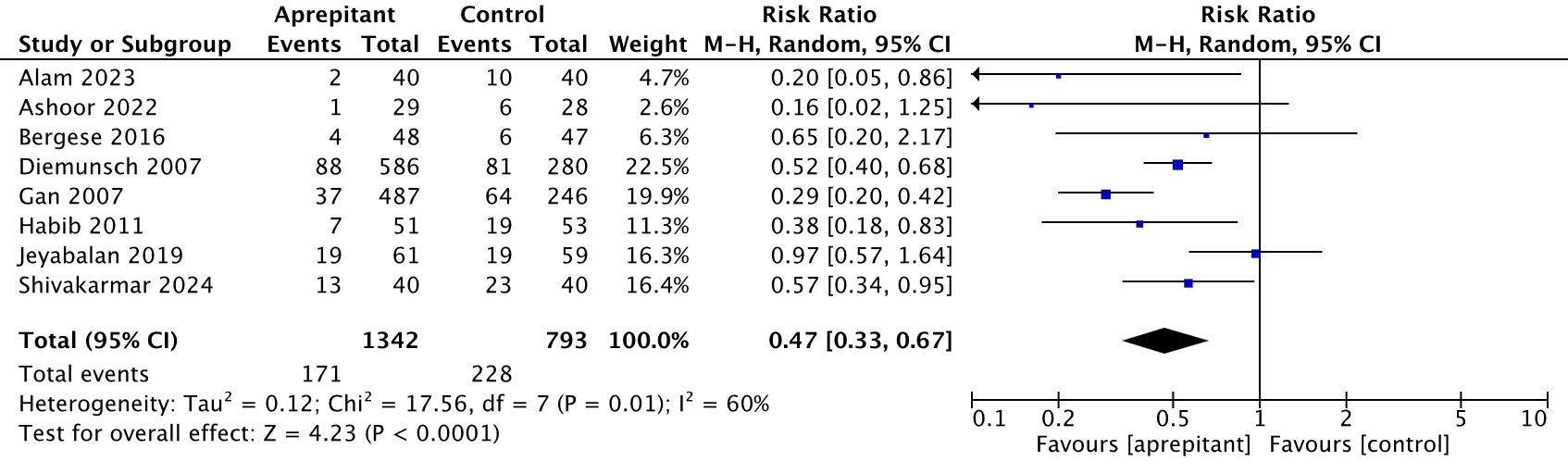


**Figure S34 Subgroup meta-analysis** Forest plot showing pooled risk ratio for the incidence of vomiting/retching between 0-24 hours after surgery. Comparison between aprepitant and other antiemetic; 95%CI, 95% confidence interval, df; degrees of freedom; I^2^, heterogeneity; M-H, random, Mantel-Haenszel random-effects model.


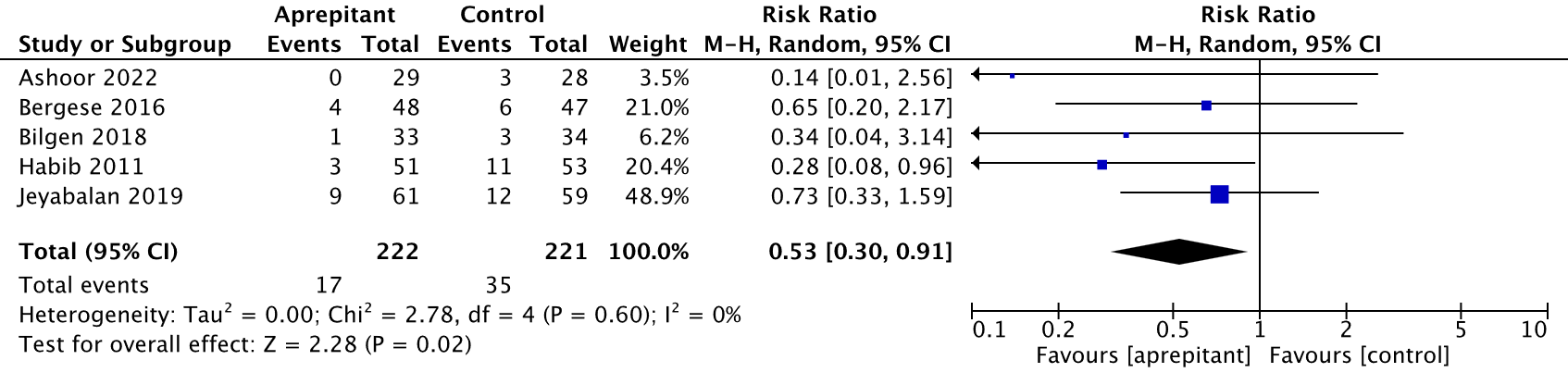


**Figure S35 Subgroup meta-analysis** Forest plot showing pooled risk ratio for the incidence of vomiting/retching between 0-24 hours after surgery. Comparison between aprepitant and other antiemetic; 95%CI, 95% confidence interval, df; degrees of freedom; I^2^, heterogeneity; M-H, random, Mantel-Haenszel random-effects model.


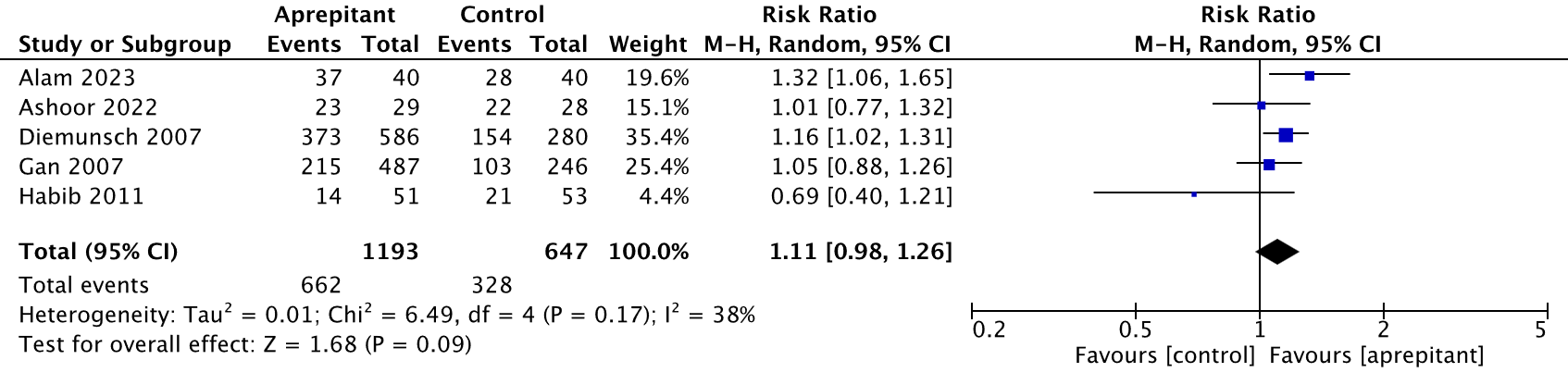


**Figure S36 Subgroup meta-analysis** Forest plot showing pooled risk ratio for the incidence of complete response rate between 0-24 hours after surgery. Comparison between aprepitant and other antiemetic; 95%CI, 95% confidence interval, df; degrees of freedom; I^2^, heterogeneity; M-H, random, Mantel-Haenszel random-effects model.


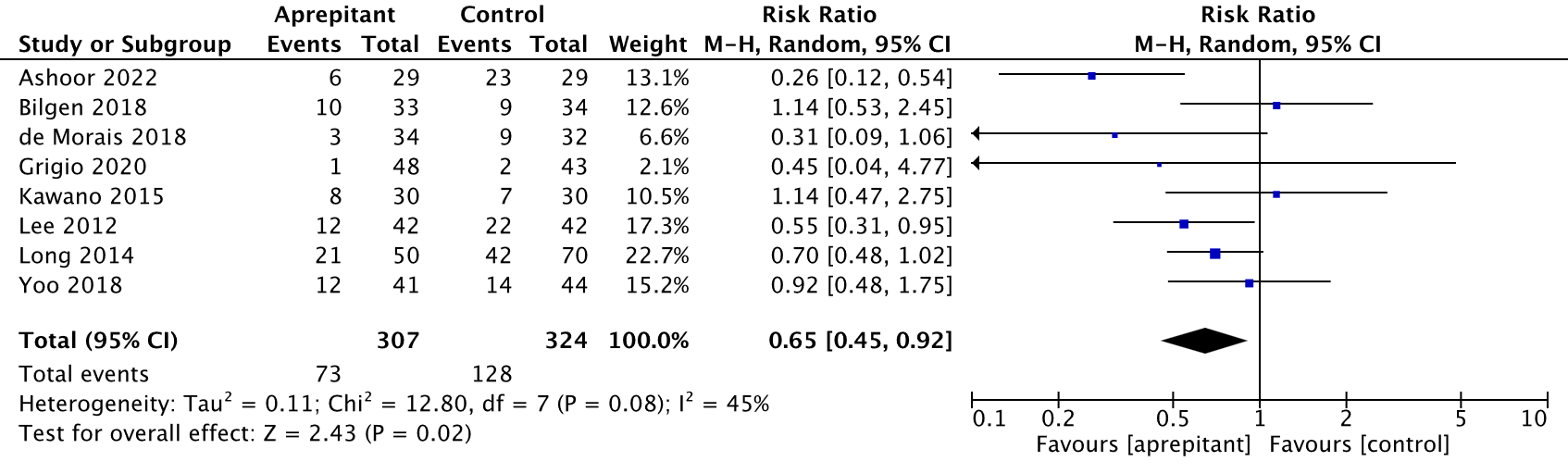


**Figure S37 Subgroup meta-analysis** Forest plot showing pooled risk ratio for the incidence of rescue antiemetic use between 0-24 hours after surgery. Comparison between “pure effect” and control; 95%CI, 95% confidence interval, df; degrees of freedom; I^2^, heterogeneity; M-H, random, Mantel-Haenszel random-effects model.


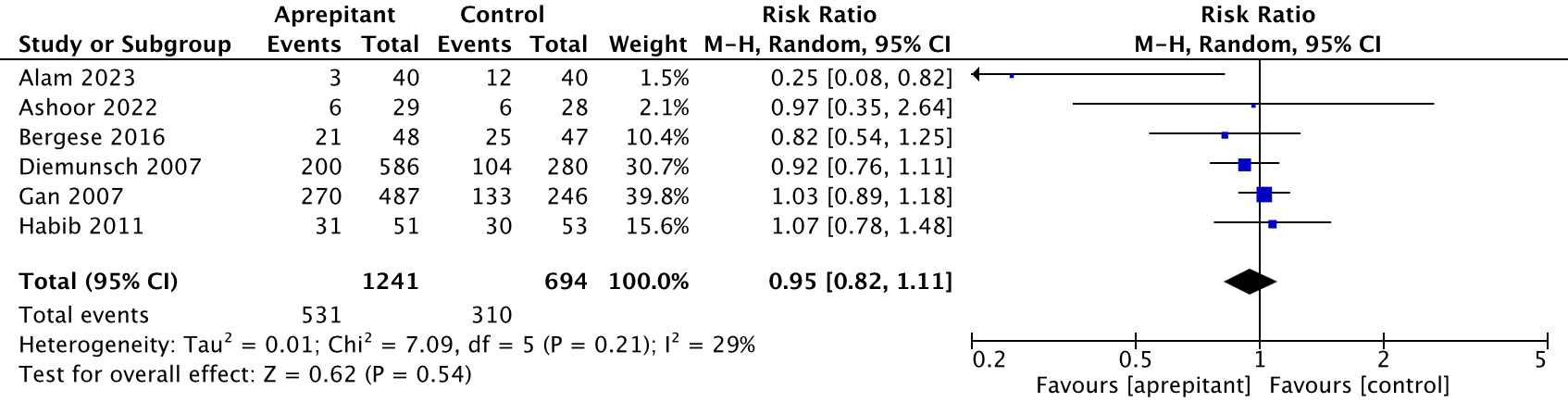


**Figure S38 Subgroup meta-analysis** Forest plot showing pooled risk ratio for the incidence of rescue antiemetic use between 0-24 hours after surgery. Comparison between aprepitant and other antiemetic; 95%CI, 95% confidence interval, df; degrees of freedom; I^2^, heterogeneity; M-H, random, Mantel-Haenszel random-effects model.


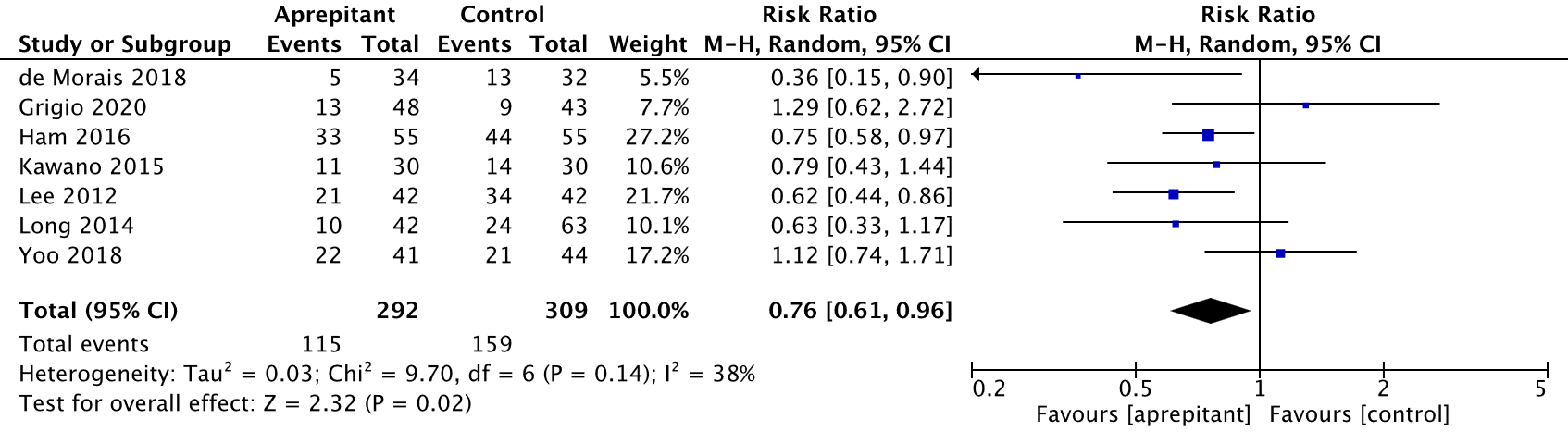


**Figure S39 Subgroup meta-analysis** Forest plot showing pooled risk ratio for the incidence of nausea between 0-24 hours after surgery. Comparison between aprepitant and control in female sex; 95%CI, 95% confidence interval, df; degrees of freedom; I^2^, heterogeneity; M-H, random, Mantel-Haenszel random-effects model.


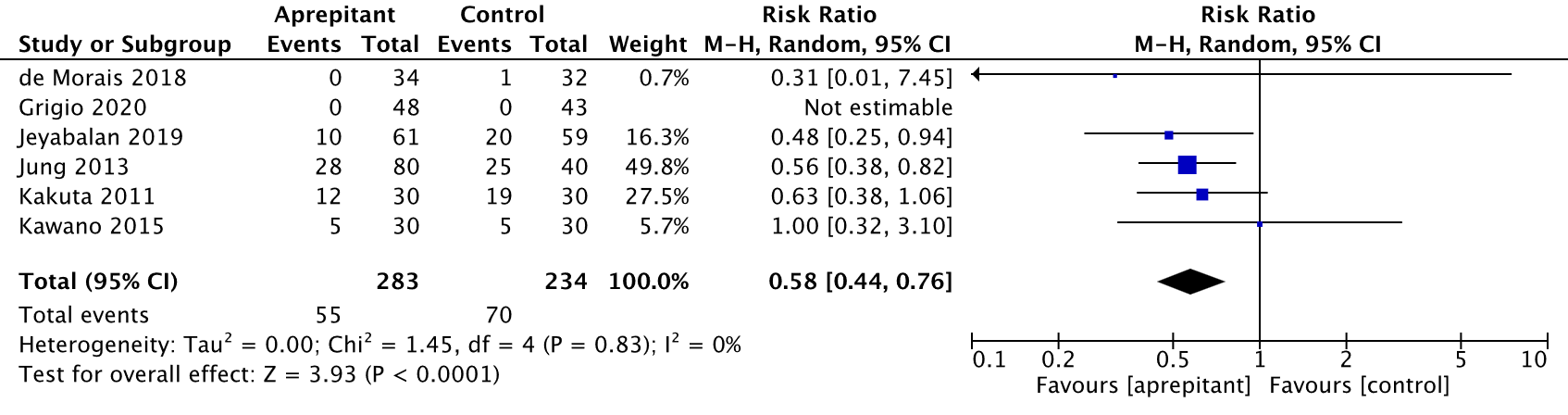


**Figure S40 Subgroup meta-analysis** Forest plot showing pooled risk ratio for the incidence of nausea between 0-2 hours after surgery. Comparison between aprepitant and control in female sex; 95%CI, 95% confidence interval, df; degrees of freedom; I^2^, heterogeneity; M-H, random, Mantel-Haenszel random-effects model.


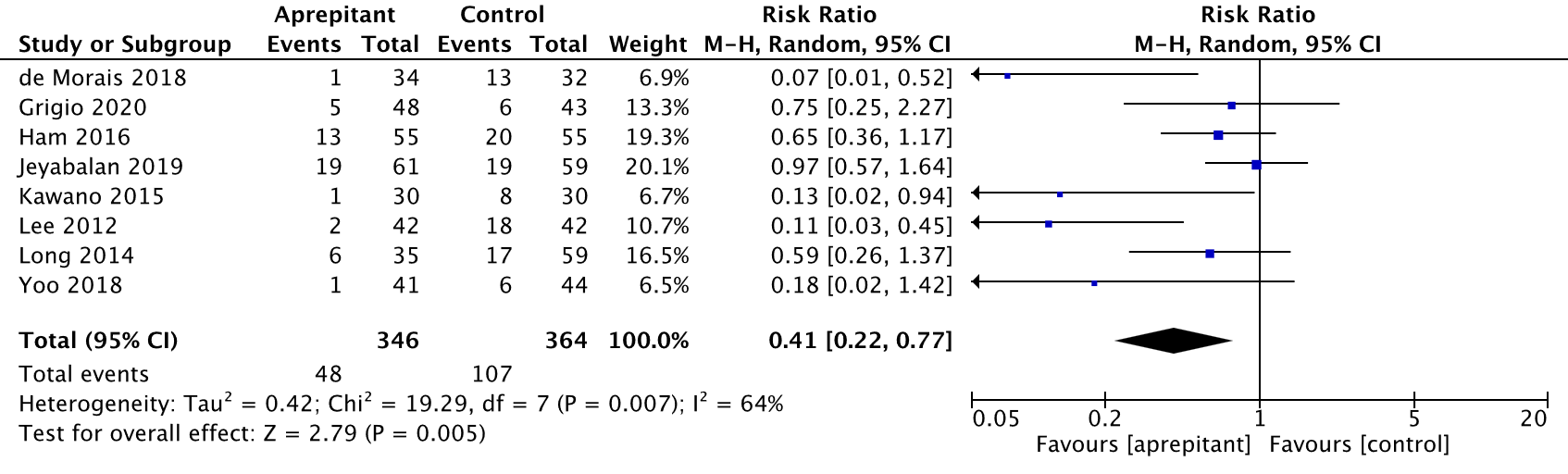


**Figure S41 Subgroup meta-analysis** Forest plot showing pooled risk ratio for the incidence of vomiting between 0-24 hours after surgery. Comparison between aprepitant and control in female sex; 95%CI, 95% confidence interval, df; degrees of freedom; I^2^, heterogeneity; M-H, random, Mantel-Haenszel random-effects model.


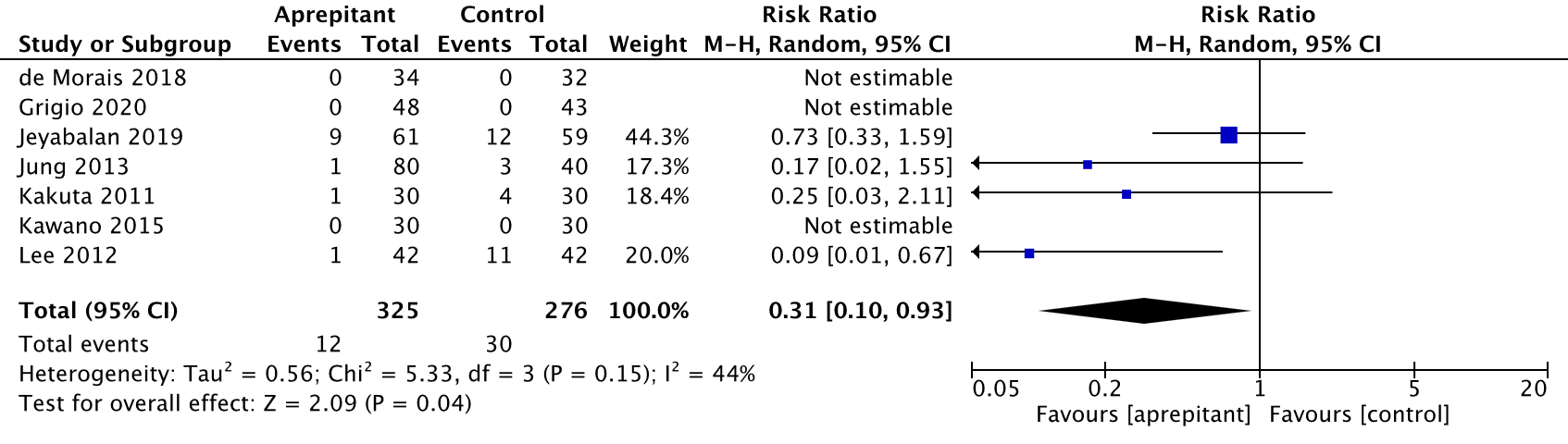


**Figure S42 Subgroup meta-analysis** Forest plot showing pooled risk ratio for the incidence of vomiting between 0-2 hours after surgery. Comparison between aprepitant and control in female sex; 95%CI, 95% confidence interval, df; degrees of freedom; I^2^, heterogeneity; M-H, random, Mantel-Haenszel random-effects model.


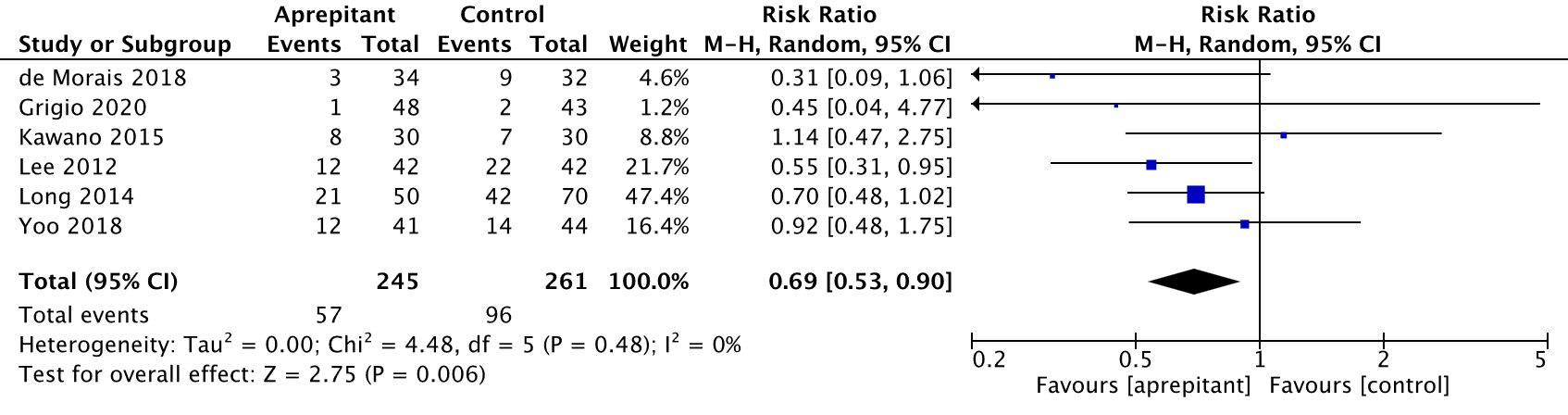


**Figure S43 Subgroup meta-analysis** Forest plot showing pooled risk ratio for the incidence of rescue antiemetic use between 0-24 hours after surgery. Comparison between aprepitant and control in female sex; 95%CI, 95% confidence interval, df; degrees of freedom; I^2^, heterogeneity; M-H, random, Mantel-Haenszel random-effects model.

- Type of surgery (high and low risk surgeries for PONV)


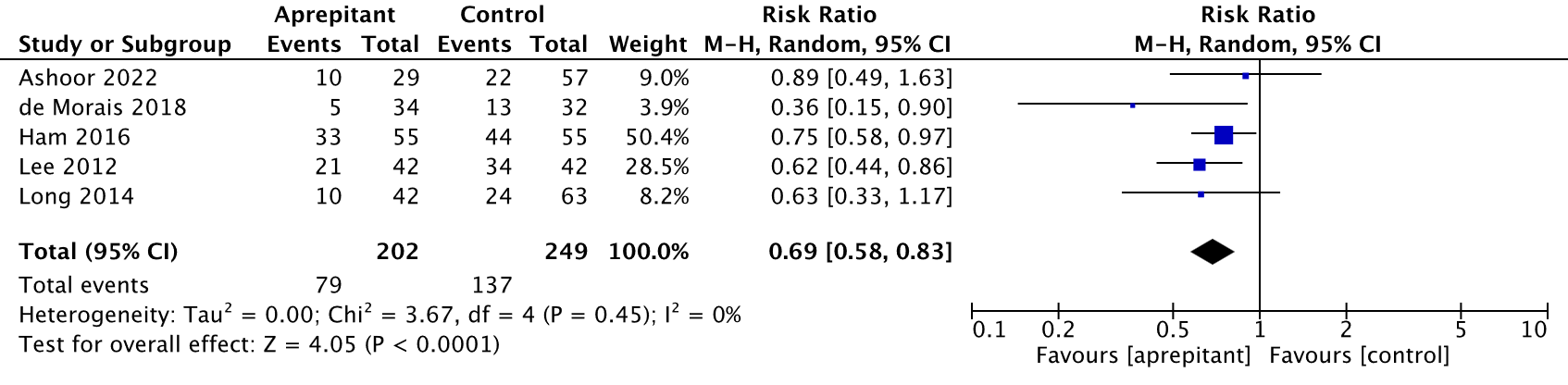


**Figure S44 Subgroup meta-analysis** Forest plot showing pooled risk ratio for the incidence of nausea between 0-24 hours after surgery. Comparison between aprepitant and control in high-risk surgeries (laparoscopic, bariatric, gynecological surgery, and cholecystectomy); 95%CI, 95% confidence interval, df; degrees of freedom; I^2^, heterogeneity; M-H, random, Mantel-Haenszel random-effects model.


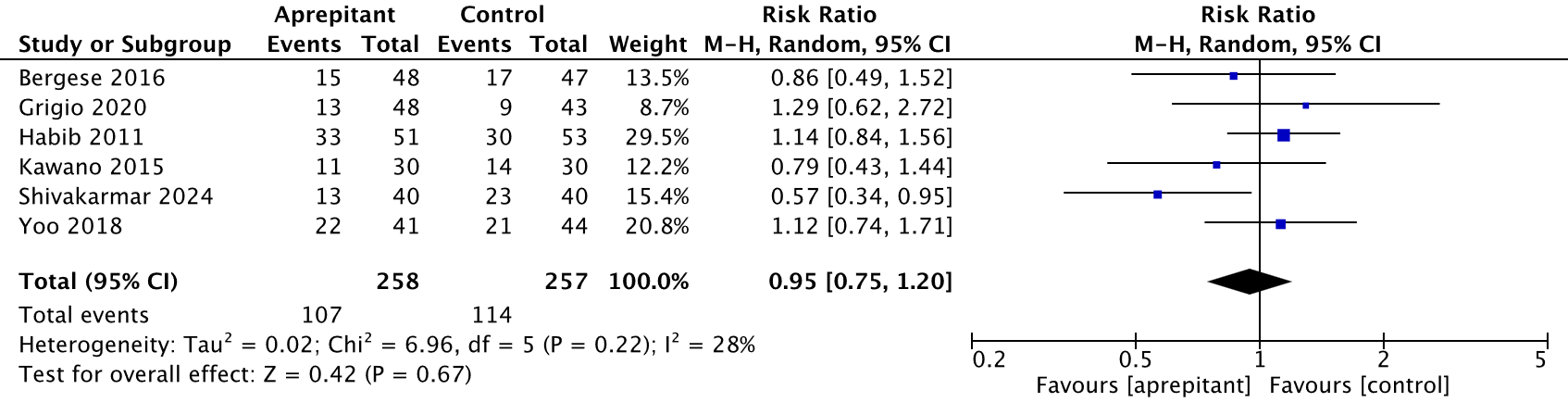


**Figure S45 Subgroup meta-analysis** Forest plot showing pooled risk ratio for the incidence of nausea between 0-24 hours after surgery. Comparison between aprepitant and control in low-risk surgeries (; 95%CI, 95% confidence interval, df; degrees of freedom; I^2^, heterogeneity; M-H, random, Mantel-Haenszel random-effects model.


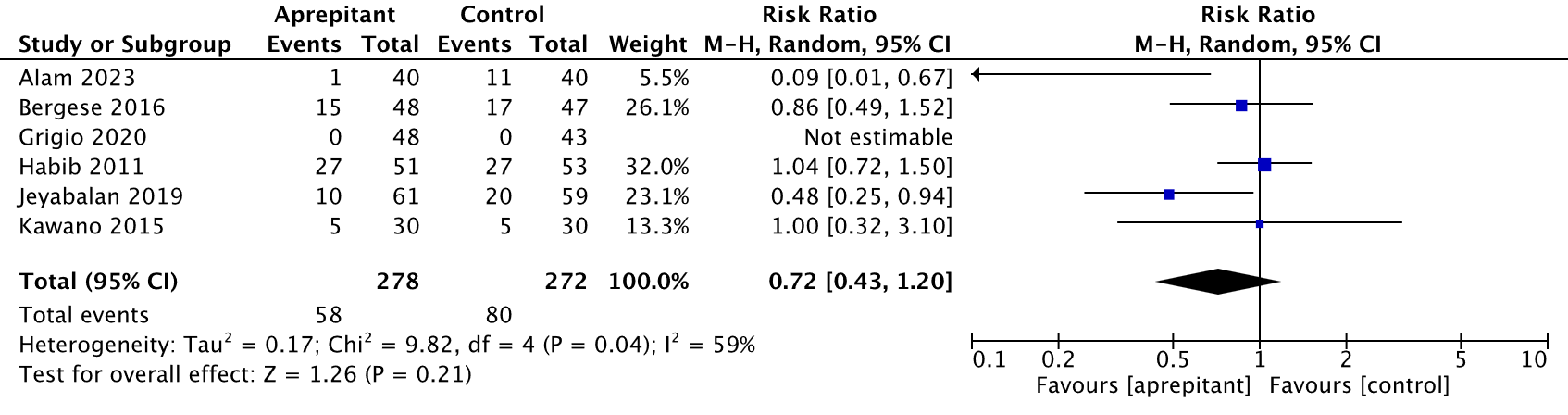


**Figure S46 Subgroup meta-analysis** Forest plot showing pooled risk ratio for the incidence of nausea between 0-2 hours after surgery. Comparison between aprepitant and control in low-risk surgeries; 95%CI, 95% confidence interval, df; degrees of freedom; I^2^, heterogeneity; M-H, random, Mantel-Haenszel random-effects model.


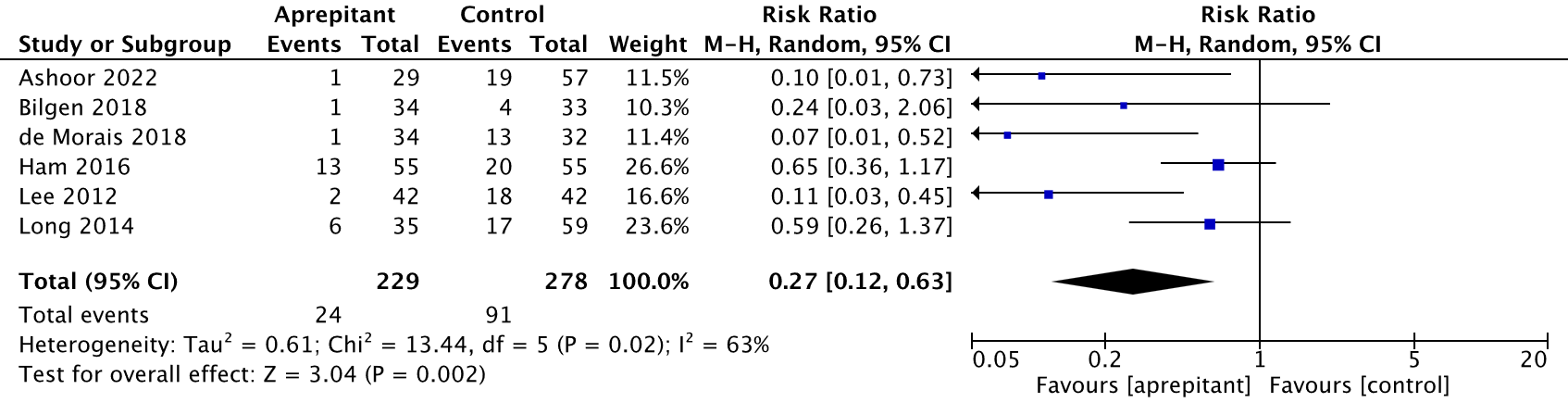


**Figure S47 Subgroup meta-analysis** Forest plot showing pooled risk ratio for the incidence of vomiting between 0-24 hours after surgery. Comparison between aprepitant and control in high-risk surgeries (laparoscopic, bariatric, gynecological surgery, and cholecystectomy); 95%CI, 95% confidence interval, df; degrees of freedom; I^2^, heterogeneity; M-H, random, Mantel-Haenszel random-effects model.


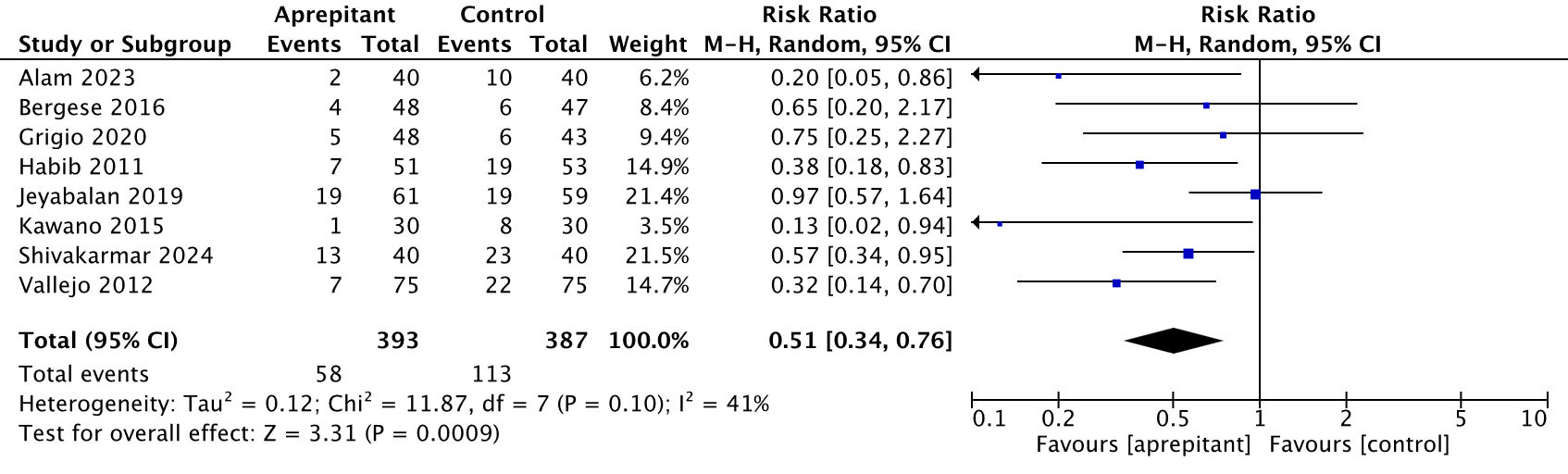


**Figure S48 Subgroup meta-analysis** Forest plot showing pooled risk ratio for the incidence of vomiting between 0-24 hours after surgery. Comparison between aprepitant and control in low-risk surgeries; 95%CI, 95% confidence interval, df; degrees of freedom; I^2^, heterogeneity; M-H, random, Mantel-Haenszel random-effects model.


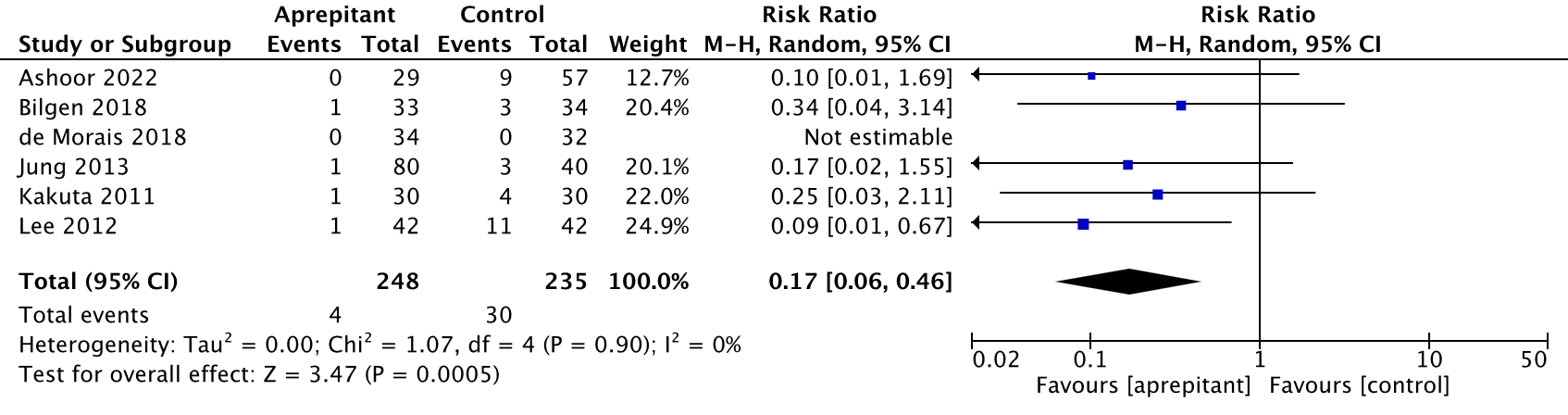


**Figure S49 Subgroup meta-analysis** Forest plot showing pooled risk ratio for the incidence of vomiting between 0-2 hours after surgery. Comparison between aprepitant and control in high-risk surgeries (laparoscopic, bariatric, gynecological surgery, and cholecystectomy); 95%CI, 95% confidence interval, df; degrees of freedom; I^2^, heterogeneity; M-H, random, Mantel-Haenszel random-effects model.


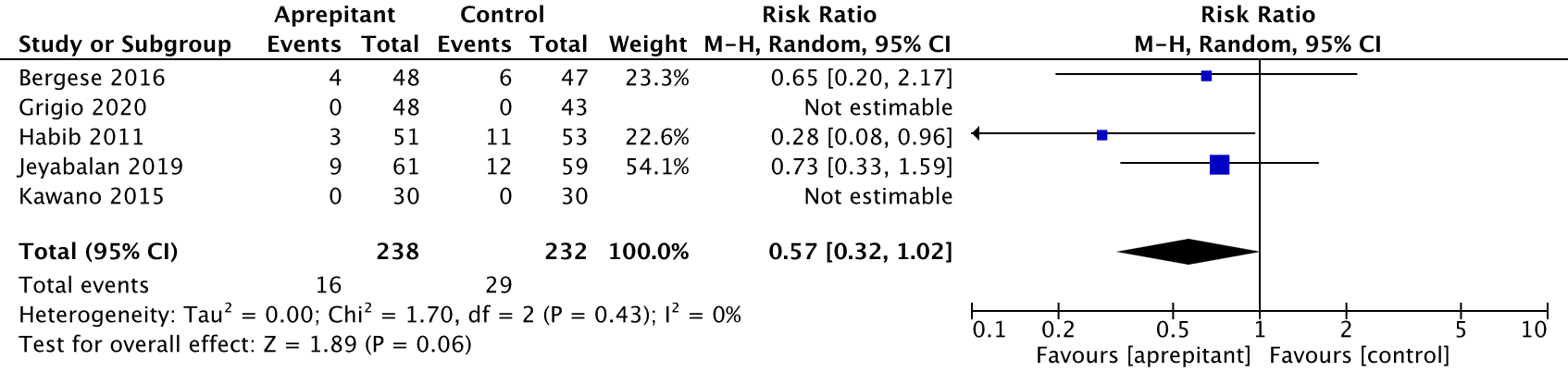


**Figure S50 Subgroup meta-analysis** Forest plot showing pooled risk ratio for the incidence of vomiting between 0-2 hours after surgery. Comparison between aprepitant and control in low-risk surgeries; 95%CI, 95% confidence interval, df; degrees of freedom; I^2^, heterogeneity; M-H, random, Mantel-Haenszel random-effects model.


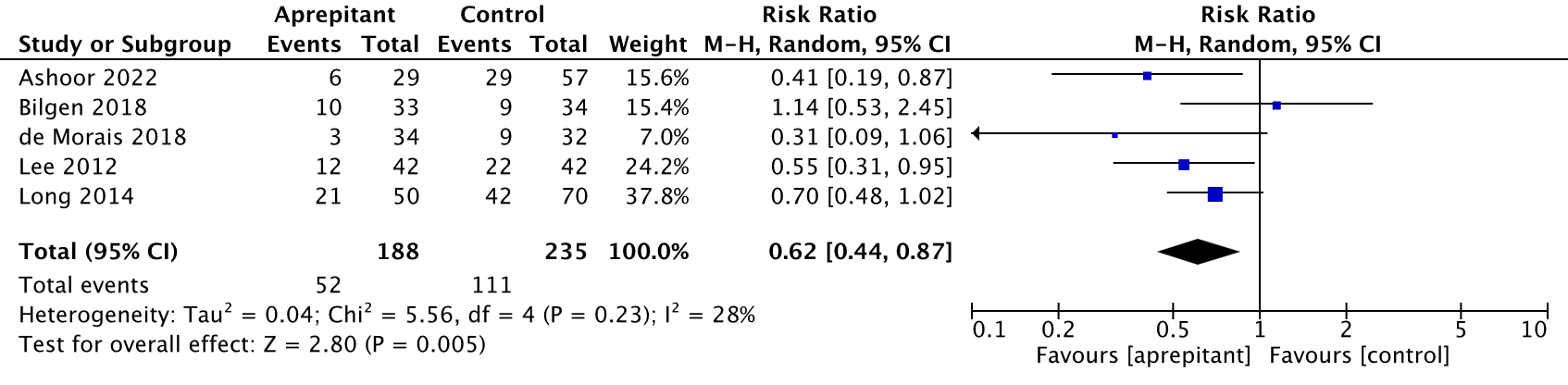


**Figure S51 Subgroup meta-analysis** Forest plot showing pooled risk ratio for the incidence of rescue antiemetic use between 0-24 hours after surgery. Comparison between aprepitant and control in high-risk surgeries (laparoscopic, bariatric, gynecological surgery, and cholecystectomy); 95%CI, 95% confidence interval, df; degrees of freedom; I^2^, heterogeneity; M-H, random, Mantel-Haenszel random-effects model.


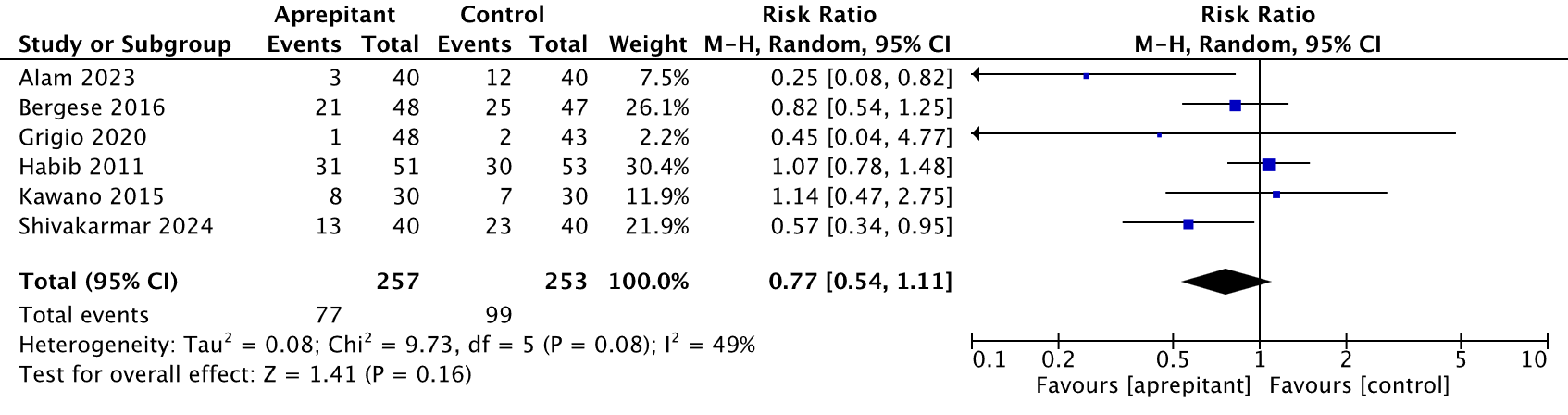


**Figure S52 Subgroup meta-analysis** Forest plot showing pooled risk ratio for the incidence of rescue antiemetic use between 0-24 hours after surgery. Comparison between aprepitant and control in low-risk surgeries; 95%CI, 95% confidence interval, df; degrees of freedom; I^2^, heterogeneity; M-H, random, Mantel-Haenszel random-effects model.

**SENSITIVITY ANALYSIS**

**
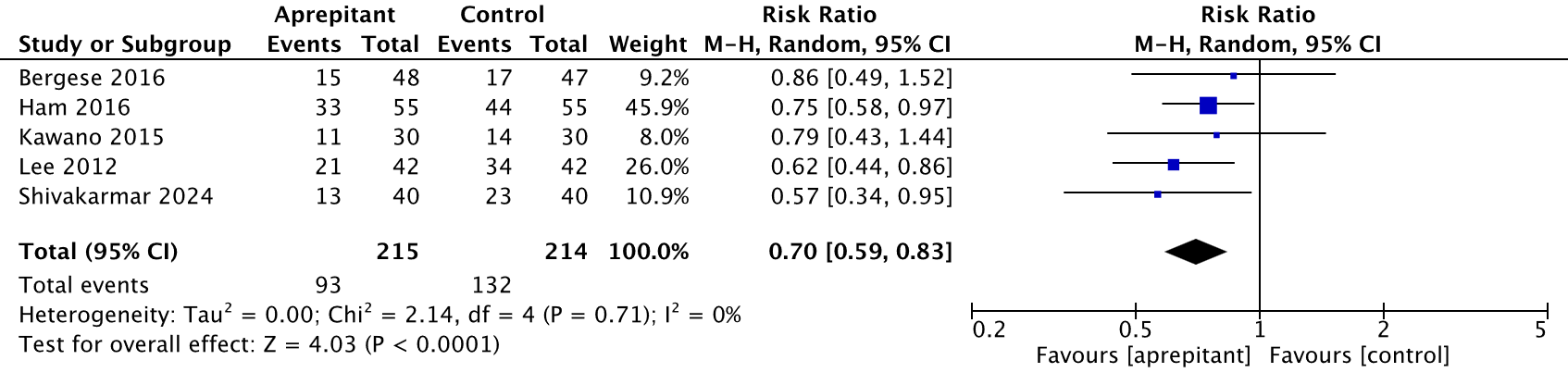
**

**Figure S53** Forest plot with low risk of bias in aprepitant studies showing pooled risk ratio for the incidence of nausea between 0-24h after surgery. 95% CI, 95% confidence interval; df; degrees of freedom; I^2^, heterogeneity; M-H, random, Mantel-Haenszel random-effects model.


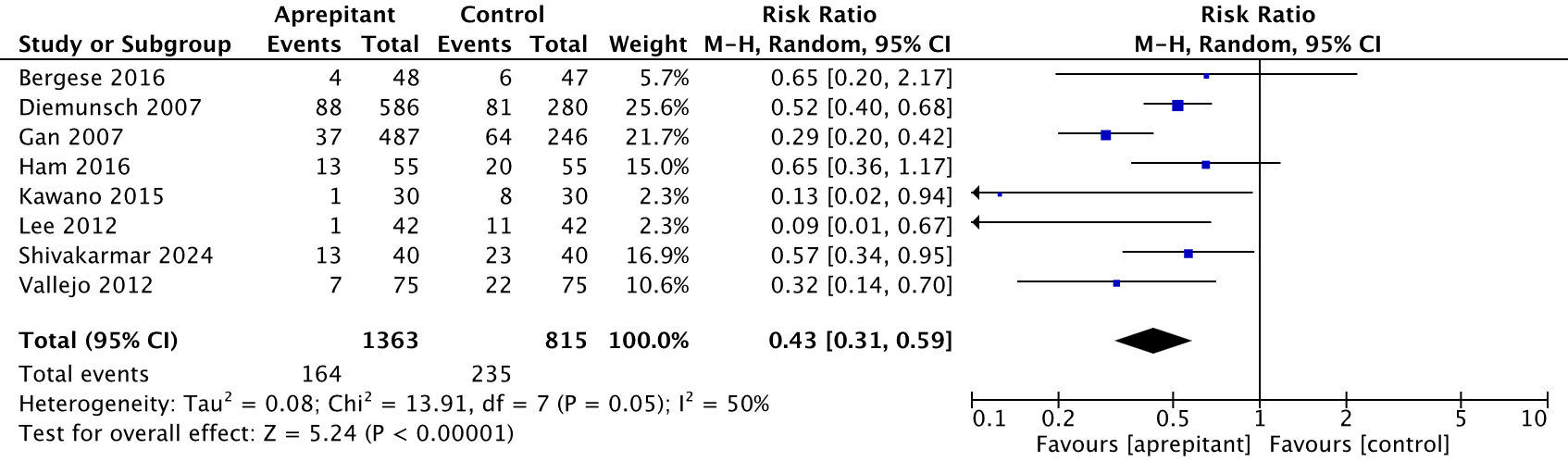


**Figure S54** Forest plot with low risk of bias in aprepitant studies showing pooled risk ratio for the incidence of vomiting between 0-24h after surgery. 95% CI, 95% confidence interval; df; degrees of freedom; I^2^, heterogeneity; M-H, random, Mantel-Haenszel random-effects model.


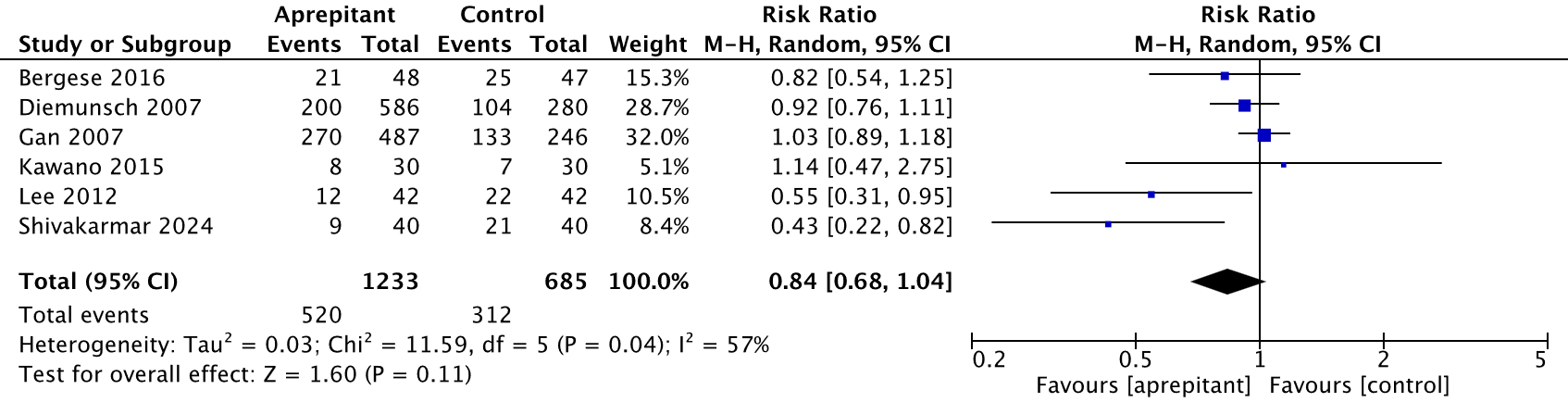


**Figure S57** Forest plot with low risk of bias in aprepitant studies showing pooled risk ratio for use of postoperatively rescue antiemetics between 0-24h after surgery. 95% CI, 95% confidence interval; df; degrees of freedom; I^2^, heterogeneity; M-H, random, Mantel-Haenszel random-effects model.
